# Supplementary material for: Mitochondrial morphology controls fatty acid utilization by changing CPT1 sensitivity to malonyl‐CoA
Source: EMBO J. 2023 Mar 14;42(11):e111901. doi: 10.15252/embj.2022111901 (PMC10233380; doi:10.15252/embj.2022111901)
Supplement: Supplementary file 1 — Expanded View Figures PDF [file EMBJ-42-e111901-s002.pdf]

Expanded View Figures

Figure EV1. Genetic perturbation of mitochondrial morphology has selective effects on mitochondrial substrate oxidation.

A Quantification of phosphorylating ADP and maximal respiration induced by palmitoyl-CoA in permeabilized HepG2 cells. $n = 5$ independent biological replicates for siCtrl and $n = 3$ independent biological replicates with six technical repeats for all other perturbations. Data represent means ± SEM, *t*-test. $^*P < 0.05$.

B Succinate-induced respiration in permeabilized HepG2 cells. $n = 3$ biological replicates with six technical repeats. Data represent means ± SEM, *t*-test. $^*P < 0.05$.

C Representative images of MFN1 KO HepG2 cells and quantification of mitochondrial length. Scale bar: 10 μm. Data represent means ± SEM, $n = 3$, *t*-test. $^*P < 0.05$.

D–F Quantification of CPT1 protein expression in HepG2 cells following expression of DN-DRP1 (D), excess nutrient treatment (E), or mi*MFN2* (F).

 © 2023 The Authors

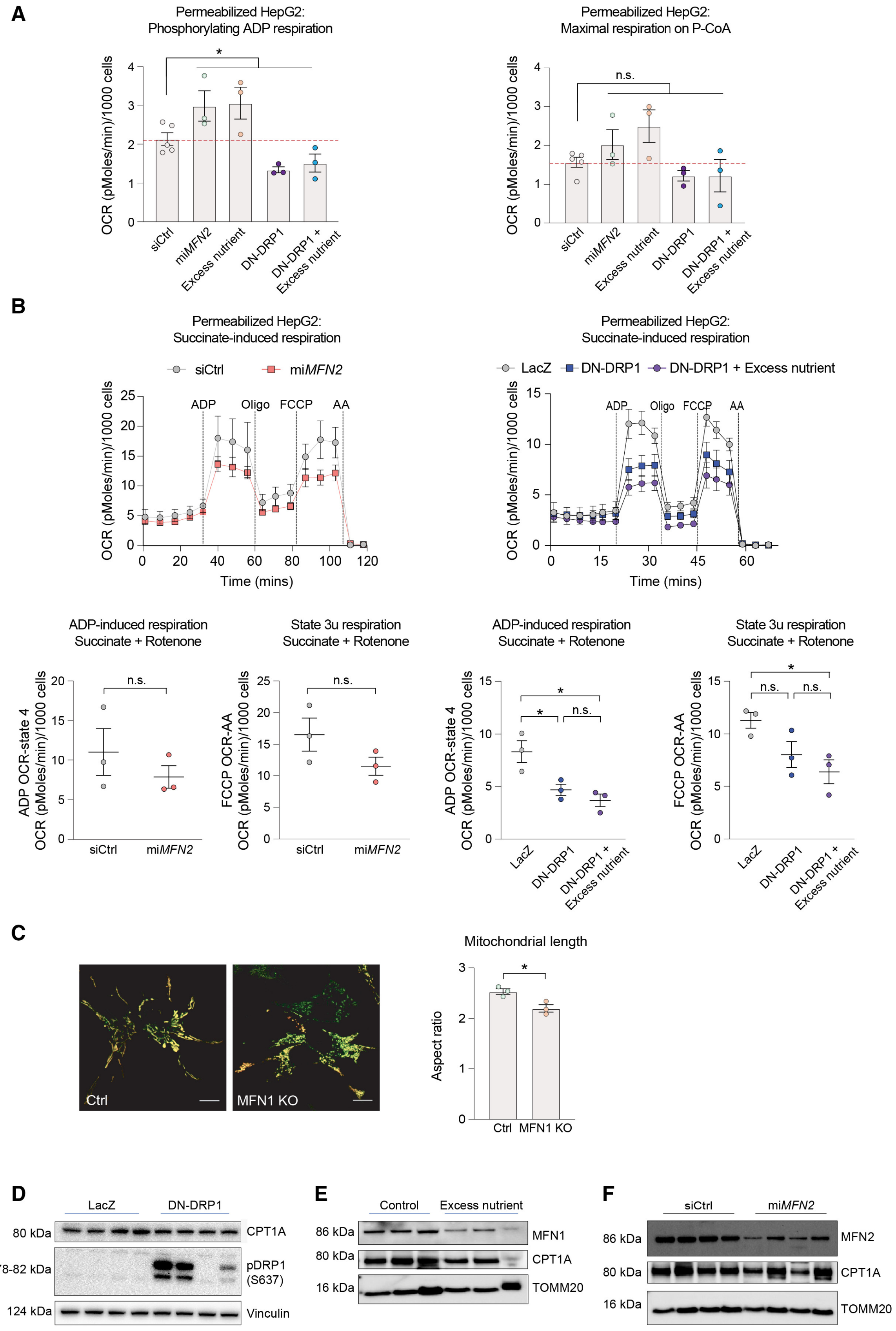
A
Permeabilized HepG2:
Phosphorylating ADP respiration
OCR (pMoles/min)/1000 cells
siCtrl
miMFN2
Excess nutrient
DN-DRP1
DN-DRP1 + Excess nutrient
Permeabilized HepG2:
Maximal respiration on P-CoA
n.s.
B
Permeabilized HepG2:
Succinate-induced respiration
siCtrl
miMFN2
LacZ
DN-DRP1
DN-DRP1 + Excess nutrient
ADP
Oligo
FCCP
AA
Time (mins)
ADP-induced respiration
Succinate + Rotenone
State 3u respiration
Succinate + Rotenone
ADP OCR-state 4
OCR (pMoles/min)/1000 cells
FCCP OCR-AA
OCR (pMoles/min)/1000 cells
n.s.
C
Mitochondrial length
Aspect ratio
Ctrl
MFN1 KO
D
LacZ
DN-DRP1
80 kDa
CPT1A
78-82 kDa
pDRP1
(S637)
124 kDa
Vinculin
E
Control
Excess nutrient
86 kDa
MFN1
80 kDa
CPT1A
16 kDa
TOMM20
F
siCtrl
miMFN2
86 kDa
MFN2
80 kDa
CPT1A
16 kDa
TOMM20

Figure EV1.

Figure EV2. Characterization of MFN2 expression in islets from obese rodent models and generation of β-Mfn2KO mice.

A Representative western blot showing MFN2 protein levels in islets derived from $n = 4$ male C57BL/6J mice fed on CHOW or HFD for 12 weeks. Porin serves as loading control. Immunoblots were quantified by ImageJ. Data represent means ± SEM *t*-test. *$P < 0.05$.

B MFN2 protein levels in islets derived from 8-week-old Zucker Lean (ZL), Zucker Fatty (ZF), Zucker Diabetic Lean (ZDL), and Zucker Diabetic Fatty (ZDF) rats. The ZF rat is a model of human obesity displaying phenotypes of hyperlipidemia and hypertension. The ZDF rat models diabetes, displaying hyperglycemia, hyperlipidemia, and hypertension although the ZDF rat strain are less obese than the ZF, they are more insulin resistant. The ZDL rat model is hyperglycemic but not hyperlipidemic. Islet protein lysates were analyzed from $n = 3$ rats per group and immunoblots were quantified using ImageJ. Data represent means ± SEM *t*-test. **$P < 0.01$. n.s, nonsignificant.

C Validation of *Mfn2* deletion in β-Mfn2KO islets by western blot analysis compared to control LoxP islets with β-actin serving as loading control. Any residual MFN2 protein in islet lysates is attributed to non-β-cell types in β-Mfn2KO islets.

D Representative images of immunohistochemical analyses of pancreatic sections from LoxP control and β-Mfn2KO mice, where β- and α-cells are stained with anti-insulin (green) and anti-glucagon (red) antibodies, respectively. Nuclei are stained with DAPI (blue). A total of 45 sections (LoxP control $n = 21$ and β-Mfn2KO mice $n = 24$) were similarly analyzed and images were subsequently quantified for ratios of β- to α-cells in the two genotypes, *t*-test. n.s, nonsignificant. Scale bar: 75 μm.

E Insulin content in LoxP control and β-Mfn2KO islets derived from $n = 4$ mice per group; technical replicates of six islets from each animal are shown. Data represent means ± SEM, *t*-test. n.s, nonsignificant.

F Quantification of dynamic insulin secretion in islet perifusion assays using LoxP control and β-Mfn2KO islets derived from $n = 4$ mice per group. β-Mfn2KO islets show a slow rising first phase secretion that remains significantly higher during the exposure to high glucose, whereas the normal response of an islet at high glucose is a sharp peak that eventually decreases to basal levels. Subsequent exposure to 40 mM KCl caused a similar excursion in insulin secretion in both genetic groups, although β-Mfn2KO islets continued to secrete at a higher rate, overall confirming the changes in insulin secretory behavior seen in static incubation assays in Fig 2G. Data represent means ± SD, AUC denotes area under the curve.

 © 2023 The Authors

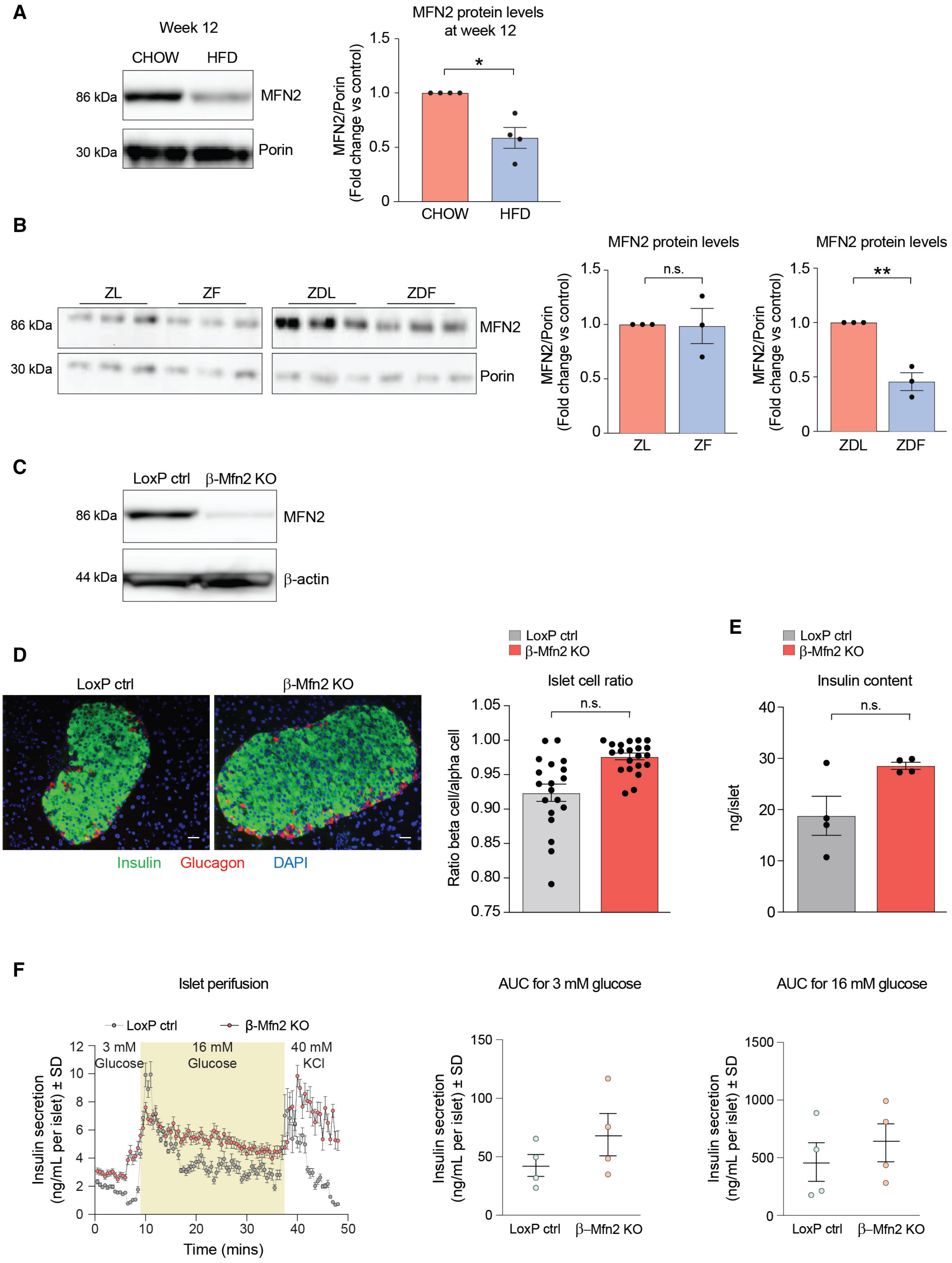
A
Week 12
CHOW HFD
86 kDa MFN2
30 kDa Porin
MFN2 protein levels at week 12
MFN2/Porin (Fold change vs control)
CHOW HFD
B
ZL ZF ZDL ZDF
86 kDa MFN2
30 kDa Porin
MFN2 protein levels
n.s.
ZL ZF
**
ZDL ZDF
C
LoxP ctrl β-Mfn2 KO
86 kDa MFN2
44 kDa β-actin
D
LoxP ctrl β-Mfn2 KO
Insulin Glucagon DAPI
Islet cell ratio
Ratio beta cell/alpha cell
E
Insulin content
ng/islet
F
Islet perifusion
3 mM Glucose
16 mM Glucose
40 mM KCl
Insulin secretion (ng/mL per islet) ± SD
Time (mins)
AUC for 3 mM glucose
AUC for 16 mM glucose
LoxP ctrl β–Mfn2 KO

Figure EV2.

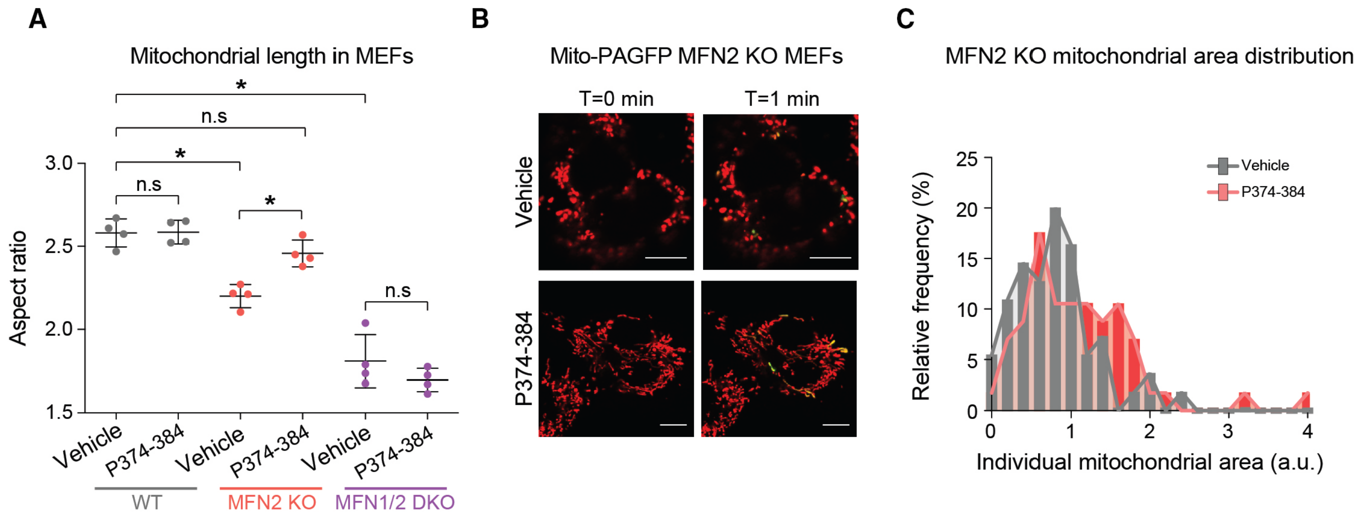

Figure EV3. Validation of the MFN2 agonist peptide MFN2-TAT-P374-384.

A Quantification of mitochondrial length in response to P374-384 treatment in mouse embryonic fibroblasts (MEFs) from the indicated genotypes. Data represent means ± SEM, $n = 4$ independent experiments with 30–50 cells analyzed per experiment, *t*-test. $*P < 0.05$, n.s, nonsignificant.

B Representative confocal images of mitochondria in MFN2 KO MEFs treated with vehicle or P374-384 and labeled with TMRE (red) and mito-PAGFP (green). Enhanced fusion was identified by 2-photon-mediated photoconversion of PAGFP. Scale bar: 10 μm.

C Quantification of mitochondrial area in MFN2 KO MEFs treated with vehicle (gray) or P374-384 (red). Data are from $n = 4$ independent experiments with 30–50 cells analyzed per experiment.

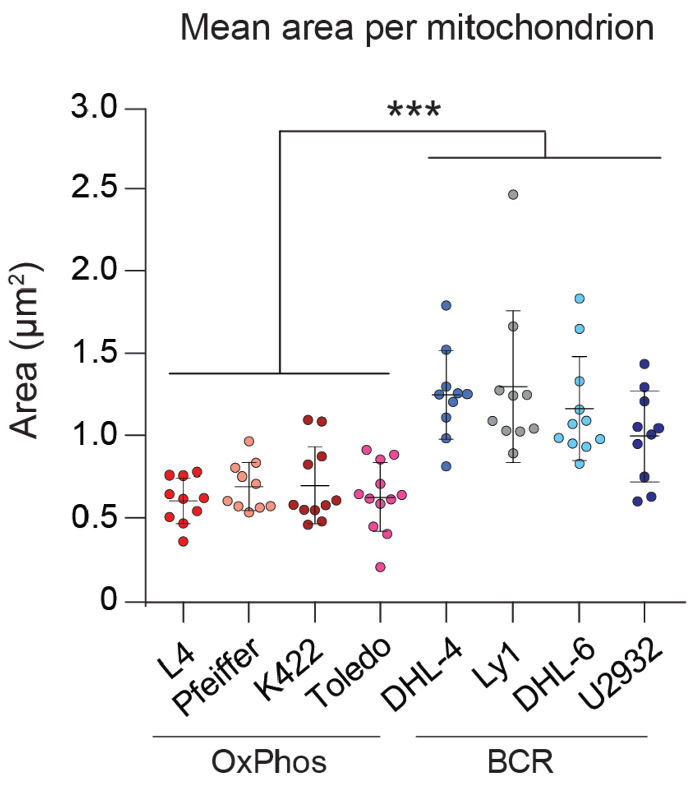

Figure EV4. Net increase in mitochondrial fragmentation in FAO prone OxPhos-DLBCLs compared with BCR-DLBCLs.

Average area of individual mitochondria in an expanded set of OxPhos- (red) and BCR-DLBCL (blue) cell lines labeled as in Fig 3A. Data represent means ± SEM with $n = 10$ cells analyzed per cell line, unpaired *t*-test with Welch's correction. $***P < 0.001$.

 © 2023 The Authors

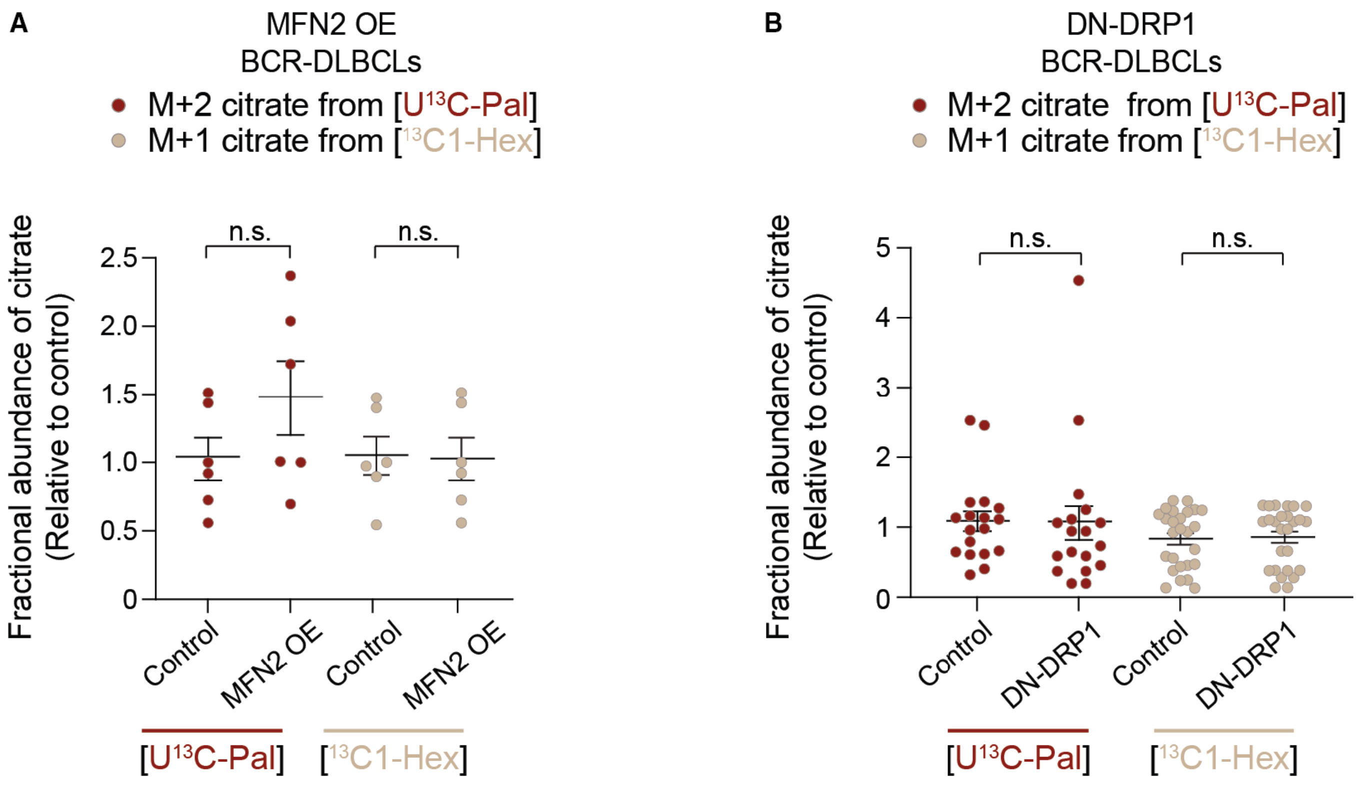

Figure EV5. Forced mitochondrial elongation in BCR-DLBCLs does not alter FAO.

A, B Fractional abundance of m + 2 citrate from [U-$^{13}C_{16}$] palmitate (LCFAO) compared with m + 1 citrate from [$^{13}C_1$] hexanoate (SCFAO) following forced mitochondrial elongation by MFN OE (A) or DN-DRP1 (B) in BCR-DLBCLs. Data represent means ± SEM and are cumulative data points from multiple individual experiments using independent cell lines as follows: n = 3–4 individual experiments in (A) using two independent BCR-DLBCL cell lines, n = 5 individual experiments in (B) using four independent BCR-DLBCL cell lines, t-test. n.s, nonsignificant. The [U-$^{13}C_{16}$] palmitate arm of these experiments is the same as shown in Fig 3F and G.

© 2023 The Authors

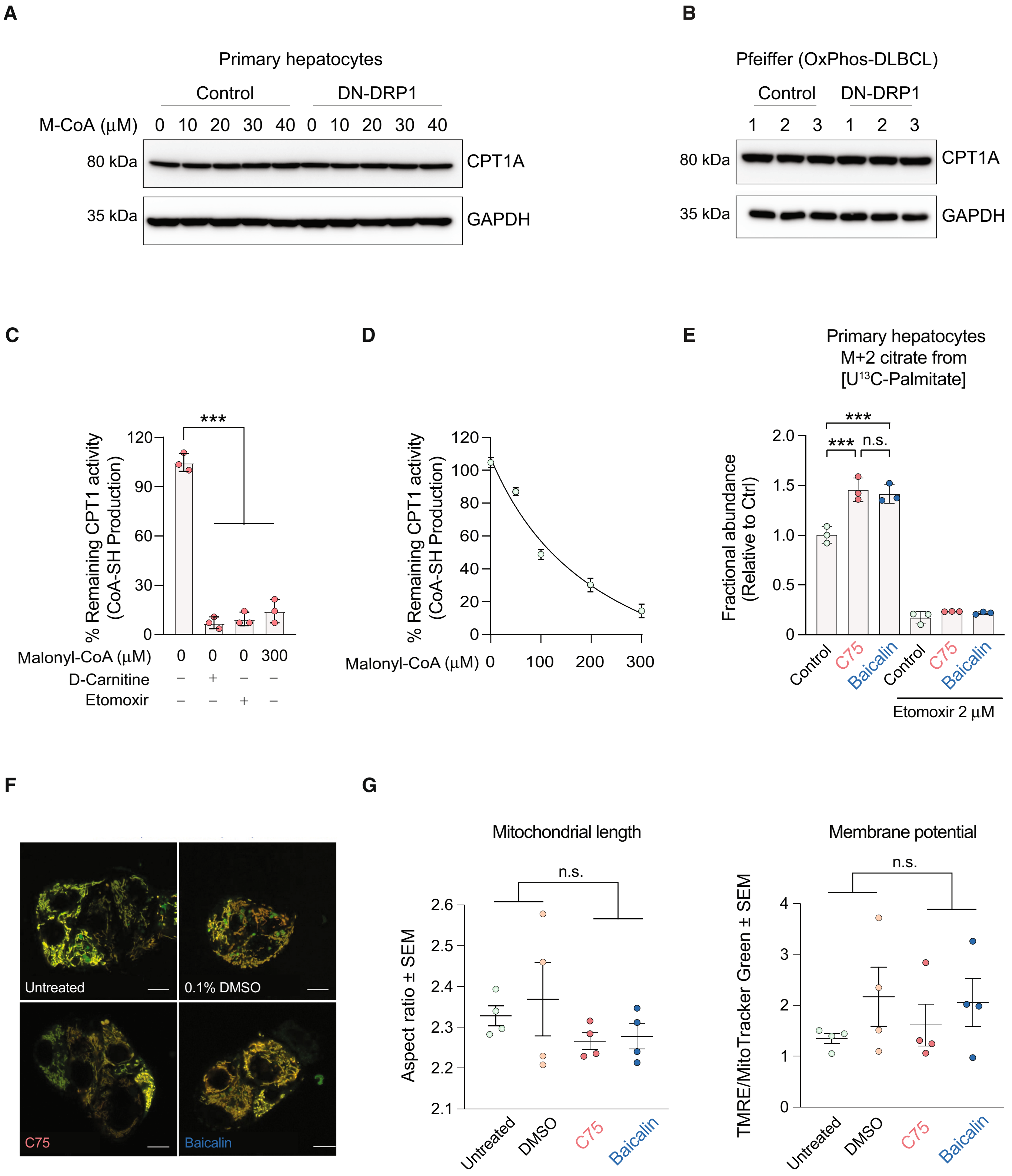

 © 2023 The Authors

Figure EV6. Validation of CPT1 activity and expression.

A, B Quantification of CPT1 protein expression in primary hepatocytes (A) and the OxPhos-DLBCL cell line Pfeiffer (B) following expression of DN-DRP1 and varying concentrations of malonyl-CoA.

C CPT1 activity in the absence or presence of 2 μM etomoxir, 0 μM palmitoyl-CoA, and 0 and 300 μM M-CoA measured in mitochondria-enriched heavy membrane fractions isolated from control primary hepatocytes. Enzyme activity was normalized to CPT1 protein levels in individual experiments. Data represent means ± SD, $n = 3$ individual experiments from primary hepatocytes, *t*-test. ***$P < 0.001$.

D Full titration curve of CPT1 activity in the presence of increasing malonyl-CoA (M-CoA) concentrations measured in mitochondria-enriched heavy membrane fractions isolated from control primary mouse hepatocytes. Enzyme activity was normalized to CPT1 protein levels in individual experiments. Data represent means ± SD, $n = 3$.

E Fractional abundance of m + 2 citrate from [U-$^{13}C_{16}$] palmitate in primary hepatocytes treated with C75 and baicalin in the absence or presence of 2 μM etomoxir. Data represent means ± SD, $n = 3$ individual experiments from primary hepatocytes, *t*-test. ***$P < 0.001$.

F, G Representative confocal images (F) and quantification (G) of mitochondrial morphology and membrane potential of mitochondria in HepG2 cells treated with vehicle, C75, or baicalin and labeled with TMRE (red) and MitoTracker Green (green). Data represent means ± SEM, $n = 4$ independent biological replicates, 20 cells imaged per independent experiment, *t*-test. Scale bar: 10 μm.

© 2023 The Authors
