# Supplementary material for: Mitochondrial morphology controls fatty acid utilization by changing CPT1 sensitivity to malonyl‐CoA
Source: EMBO J. 2023 Mar 14;42(11):e111901. doi: 10.15252/embj.2022111901 (PMC10233380; doi:10.15252/embj.2022111901)
Supplement: Supplementary file 2 — Table EV1 [file EMBJ-42-e111901-s001.pdf]

| Treatment Group | Aspect Ratio; n=3 (Fig 1B) | Etomoxir-Sensitive OCR; n=3 (Fig 1B) | Average Aspect Ratio Values (Fig 1C) | Average Etomoxir-Sensitive OCR Values (Fig 1C) |
|---|---|---|---|---|
| Excess nutrient | 1.896 | 3.583 | 2.183 | 3.056 |
| | 2.345 | 3.333 | | |
| | 2.308 | 2.250 | | |
| mi*MFN2* | 2.382 | 3.758 | 2.371 | 2.983 |
| | 2.390 | 2.666 | | |
| | 2.340 | 2.524 | | |
| siCtrl | 2.743 | 1.786 | 2.656 | 1.871 |
| | 2.654 | 1.978 | | |
| | 2.571 | 1.849 | | |
| DN-DRP1 | 2.816 | 1.110 | 2.906 | 1.516 |
| | 2.955 | 1.910 | | |
| | 2.947 | 1.528 | | |
| DN-DRP1+ Excess nutrient | 2.659 | 1.262 | 2.735 | 1.344 |
| | 2.751 | 1.495 | | |
| | 2.795 | 1.275 | | |

**Expanded View Table 1. Mitochondrial length etomoxir sensitive OCR stimulated by palmitoyl-CoA and carnitine in permeabilized HepG2 cells**

Quantitation of mitochondrial length (aspect ratio, AR) along with correlating FAO. FAO was determined based on etomoxir sensitive OCR stimulated by palmitoyl-CoA and carnitine in permeabilized HepG2 cells in the presence of ADP. Data from columns one and two.
