# Supplementary material for: Mitochondrial morphology controls fatty acid utilization by changing CPT1 sensitivity to malonyl‐CoA
Source: EMBO J. 2023 Mar 14;42(11):e111901. doi: 10.15252/embj.2022111901 (PMC10233380; doi:10.15252/embj.2022111901)
Supplement: Supplementary file 4 — PDF+ [file EMBJ-42-e111901-s004.pdf]

Article

TRANSPARENT PROCESS OPEN ACCESS

THE EMBO JOURNAL

Mitochondrial morphology controls fatty acid utilization by changing CPT1 sensitivity to malonyl-CoA

Jennifer Ngo[1,2,3,4,†], Dong Wook Choi[5,6,†], Illana A Stanley[5], Linsey Stiles[1,2], Anthony J A Molina[7], Pei-Hsuan Chen[5], Ana Lako[5], Isabelle Chiao Han Sung[5,12], Rishov Goswami[5], Min-young Kim[6], Nathanael Miller[1,8], Siyouneh Baghdasarian[1], Doyeon Kim-Vasquez[1], Anthony E Jones[2], Brett Roach[1], Vincent Gutierrez[1], Karel Erion[1], Ajit S Divakaruni[2], Marc Liesa[1,4,2,9], Nika N Danial[5,10,11,*,‡] & Orian S Shirihai[1,2,**,‡]

Abstract

Changes in mitochondrial morphology are associated with nutrient utilization, but the precise causalities and the underlying mechanisms remain unknown. Here, using cellular models representing a wide variety of mitochondrial shapes, we show a strong linear correlation between mitochondrial fragmentation and increased fatty acid oxidation (FAO) rates. Forced mitochondrial elongation following MFN2 over-expression or DRP1 depletion diminishes FAO, while forced fragmentation upon knockdown or knockout of MFN2 augments FAO as evident from respirometry and metabolic tracing. Remarkably, the genetic induction of fragmentation phenocopies distinct cell type-specific biological functions of enhanced FAO. These include stimulation of gluconeogenesis in hepatocytes, induction of insulin secretion in islet β-cells exposed to fatty acids, and survival of FAO-dependent lymphoma subtypes. We find that fragmentation increases long-chain but not short-chain FAO, identifying carnitine O-palmitoyltransferase 1 (CPT1) as the downstream effector of mitochondrial morphology in regulation of FAO. Mechanistically, we determined that fragmentation reduces malonyl-CoA inhibition of CPT1, while elongation increases CPT1 sensitivity to malonyl-CoA inhibition. Overall, these findings underscore a physiologic role for fragmentation as a mechanism whereby cellular fuel preference and FAO capacity are determined.

Keywords CPT1; fatty acid oxidation; fission; fusion; mitochondrial dynamics
Subject Categories Autophagy & Cell Death; Cancer; Metabolism
DOI 10.15252/embj.2022111901 | Received 29 June 2022 | Revised 17 January 2023 | Accepted 2 February 2023 | Published online 14 March 2023
The EMBO Journal (2023) 42: e111901
See also: **EL Seifert & G Hajnóczky** (June 2023)

Introduction

Mitochondria go through continuous cycles of fusion and fission, a process termed mitochondrial dynamics (Sebastián *et al*, 2017; Eisner *et al*, 2018; Sprenger & Langer, 2019; Giacomello *et al*, 2020; Kraus *et al*, 2021). Fusion is mediated by the interaction of the two mitochondrial outer membrane proteins, MFN1 and 2, and by the mitochondrial inner membrane protein OPA1. Fission is mediated by the recruitment and oligomerization of DRP1 on the mitochondrial outer membrane (Frank *et al*, 2001). Fission events produce smaller mitochondria to allow for their removal by mitophagy, acting as a quality control mechanism (Twig *et al*, 2008b). While each

1 Division of Endocrinology, Department of Medicine, David Geffen School of Medicine, Molecular Biology Institute, UCLA, CA, Los Angeles, USA
2 Department of Molecular and Medical Pharmacology, UCLA, CA, Los Angeles, USA
3 Department of Chemistry & Biochemistry, UCLA, CA, Los Angeles, USA
4 Molecular Biology Institute, UCLA, CA, Los Angeles, USA
5 Department of Cancer Biology, Dana-Farber Cancer Institute, Harvard Medical School, MA, Boston, USA
6 Department of Biochemistry, College of Natural Sciences, Chungnam National University, Daejeon, South Korea
7 Division of Geriatrics and Gerontology, UCSD School of Medicine, CA, La Jolla, USA
8 Obesity Research Center, Molecular Medicine, Boston University School of Medicine, MA, Boston, USA
9 Molecular Biology Institute of Barcelona, IBMB-CSIC, Barcelona, Spain
10 Department of Medical Oncology, Dana-Farber Cancer Institute, Harvard Medical School, MA, Boston, USA
11 Department of Medicine, Harvard Medical School, MA, Boston, USA
12 Yale-NUS College, University Town, NUS, Singapore
*Corresponding author. Tel: +1 617 6326436; E-mail: nika_danial@dfci.harvard.edu
**Corresponding author. Tel: +1 617 2308570; E-mail: oshirihai@mednet.ucla.edu
[†]These authors contributed equally to this work
[‡]These authors contributed equally to this work

© 2023 The Authors. Published under the terms of the CC BY 4.0 license.

mitochondrion within a cell goes through fusion and fission events, periodically, a cellular signal can mandate a global reduction in fusion, fission, or both, causing a cellular change in mitochondrial architecture. An overall shortening of mitochondria due to increased fission, reduced fusion, or both is termed fragmentation. Fragmentation has been observed in numerous pathologic and stress conditions, where damaged mitochondria may be more readily removed following fragmentation (Liesa & Shirihai, 2013). However, fragmentation does not always result in increased mitophagy leaving open a potential for mitochondrial fragmentation to have an adaptive role. Multiple studies have examined the potential role of mitochondrial dynamics in metabolic regulation in a variety of cell types and tissues (Mahdaviani *et al*, 2017; Nagdas *et al*, 2019; Labbé *et al*, 2021; Valera-Alberni *et al*, 2021). However, whether metabolic consequences resulting from alterations in mitochondrial architecture are due to pleiotropic changes in mitochondrial bioenergetics or stem from selective changes in fuel patterns is unclear. The observation that the balance of energy supply and demand correlates with changes in mitochondrial architecture raises the possibility that specific alterations in mitochondrial architecture may be adaptive to fuel preference and availability (Liesa & Shirihai, 2013). In particular, obesity has been shown to increase fission and reduce mitochondrial fusion, leading to mitochondrial fragmentation, while mitophagy is not increased but is rather decreased (Frank *et al*, 2001; Bach *et al*, 2005; Molina *et al*, 2009; Hernández-Alvarez *et al*, 2010; Galloway *et al*, 2014). Nutrient-induced mitochondrial fragmentation could be reproduced in culture models by exposure to excess lipids in β-cells, muscle, liver, and endothelial cells (Molina *et al*, 2009; Shenouda *et al*, 2011; Gao *et al*, 2014a; Gasier *et al*, 2020). Altered mitochondrial architecture under these conditions is associated with changes in the expression of mitochondrial dynamics proteins, including downregulation of MFN2 and the induction as well as posttranslational modification of DRP1 (Liesa *et al*, 2009). Mitochondrial fragmentation under conditions of metabolic stress has been previously thought to represent a maladaptive destruction of normal mitochondrial function (Ngo *et al*, 2021). However, physiological fragmentation during substantial increases in fatty acid utilization, such as brown adipose tissue in response to adrenergic stimulation, suggests fragmentation may have an adaptive role in the regulation of fuel utilization and preference (Liesa & Shirihai, 2013; Mahdaviani *et al*, 2017).

The above observations link lipid utilization to mitochondrial fragmentation, yet whether the degree of fragmentation is strongly associated with the extent of lipid utilization in a causal way, as well as the mechanism underlying the influence of fragmentation on lipid utilization have not been elucidated. In this study, we examined if and how mitochondrial architecture controls lipid utilization. We quantified the relationship between the extent of mitochondrial elongation or fragmentation with mitochondrial capacity to consume fatty acids in multiple independent cellular models. We show that fragmentation increases both the lipid oxidation capacity and preference for lipids as a fuel. We identify CPT1 as the main target by which fragmentation regulates lipid utilization. We further demonstrate that the effect of fragmentation on CPT1 sensitivity to its allosteric inhibitor, malonyl-CoA, is a mechanism whereby mitochondrial architecture regulates fatty acid oxidation. The results of this study indicate that mitochondrial architecture can have an adaptive role in metabolism. The capacity of mitochondrial dynamics to regulate cellular fuel preference or fuel decisions is also intriguing given the changes in fuel utilization patterns that accompany several pathologies beyond cardiometabolic disease, including inflammation and cancer.

Results

Mitochondrial fatty acid oxidation capacity correlates with mitochondrial architecture

To initially assess the relationship between mitochondrial architecture and the capacity to utilize fatty acids, we determined the correlation coefficient between mitochondrial aspect ratio as a measure of mitochondrial morphology and FAO in HepG2 hepatocarcinoma cells under multiple conditions (Fig 1A). These included genetic manipulations that force mitochondrial fragmentation, as in microRNA-mediated *MFN2* knockdown (mi*MFN2*) (Sebastián *et al*, 2012), or elongation, as in overexpression of a dominant negative DRP1 K38A mutant (DN-DRP1). To further substantiate this correlation, we also tested nutrient combinations previously shown to increase FAO, as in excess glucose and fat, mimicking glucolipotoxicity in cardiometabolic diseases (25 mM glucose + 250 μM palmitate), hereafter referred to as "excess nutrient" (Molina *et al*, 2009). FAO was quantified as the etomoxir-sensitive component of oxygen consumption rate (OCR) in respirometry assays. More specifically, etomoxir-sensitive OCR was quantified as the difference between

Figure 1. Mitochondrial fragmentation drives FAO and gluconeogenesis in hepatocytes.

A Experimental design to assess the relationship between FAO and mitochondrial morphology in HepG2 cells.

B Quantification of correlation between mitochondrial length (aspect ratio, AR) and FAO. FAO was determined based on etomoxir-sensitive OCR stimulated by palmitoyl-CoA and carnitine in permeabilized HepG2 cells in the presence of ADP. $n = 3$ biological replicates with 6 technical replicates. AR data are from $n = 3$ independent biological replicates. For each condition, the mean from 20 to 50 cells analyzed per experiment are indicated. Representative confocal TMRE images of mitochondria and OCR traces are shown for cells cultured in excess nutrient conditions in the absence (Bi) or presence of DN-DRP1 (Bii). Vehicle denotes an absence of substrates. Scale bar in (Bi, Bii): 10 μm. Data represent means ± SEM.

C Mean values of mitochondrial AR and FAO capacity from independent biological replicates and aggregated values across the indicated experimental conditions measured as in (B). Data represent means ± SEM, $n = 3$, *t*-test.

D Schematic of stable isotope tracing analysis to measure FAO using [U-$^{13}C_{16}$] palmitate.

E–I Fractional abundance of m + 2 citrate from [U-$^{13}C_{16}$] palmitate following forced mitochondrial fragmentation in mi*MFN2* (E) and *MFN1* KO HepG2 cells (F) or following forced mitochondrial elongation via MFN1 OE (G), si*DRP1* (H) or DN-DRP1 (I) in primary hepatocytes. Data represent means ± SEM, $n = 3$ independent biological replicates, *t*-test. $*P < 0.05$, $**P < 0.01$.

J–M Gluconeogenesis assessed by glucose production in response to lactate and pyruvate in primary mouse hepatocytes following *DRP1* knockdown (J), expression of DN-DRP1 (K), MFN1 OE (L), or MFN2 OE (M). Data represent means ± SD, $n = 3$ independent biological replicates, *t*-test. $**P < 0.01$, $***P < 0.001$.

© 2023 The Authors

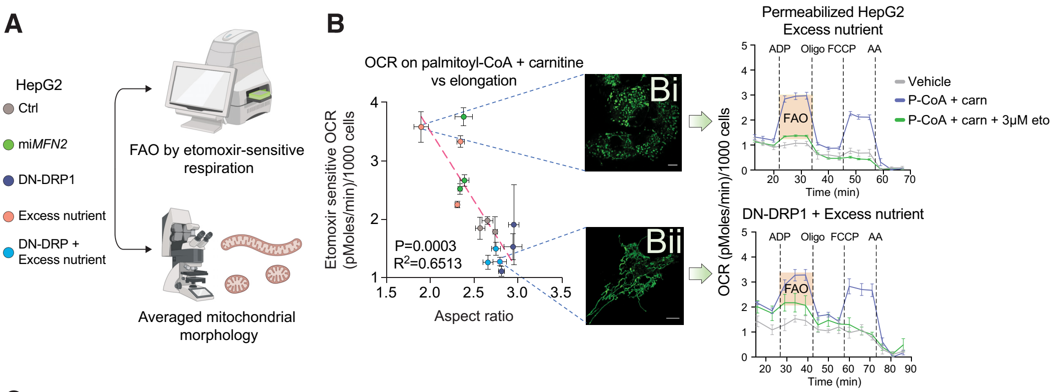

C

Treatment group	Aspect Ratio ± SEM	FAO (pmoles/min)± SEM
Ctrl	2.656 ± 0.049	1.871 ± 0.05654
mi*MFN2*	2.371 ± 0.015	2.983 ± 0.3896
DN-DRP1	2.906 ± 0.045	1.516 ± 0.2309
Excess nutrient	2.183 ± 0.144	3.056 ± 0.409
DN-DRP1 + Excess nutrient	2.735 ± 0.040	1.344 ± 0.07569

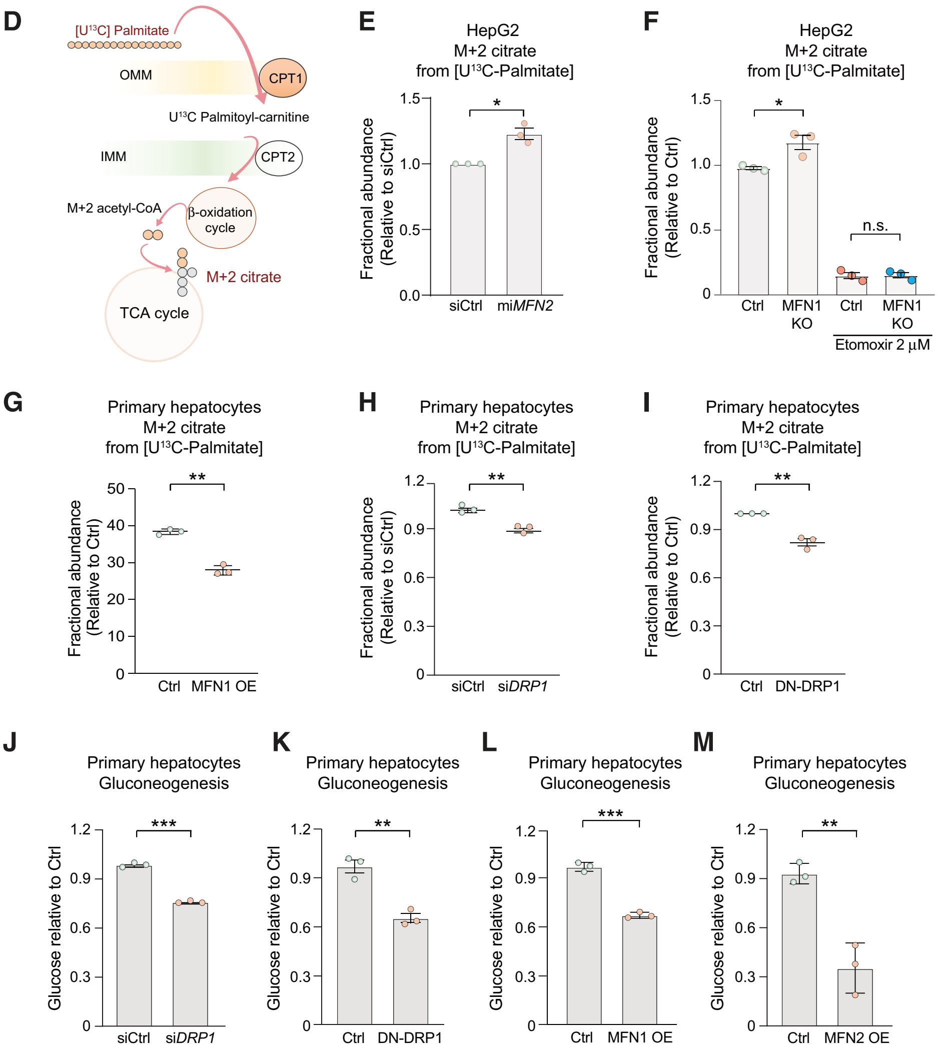

Figure 1.

respiration induced by palmitoyl-CoA + Carnitine + ADP and that induced by palmitoyl-CoA + Carnitine + Etomoxir + ADP, whereby "vehicle" is void of saturating levels of fuel and consists only of endogenous substrates. This allows for measuring the capacity to only oxidize palmitoyl-CoA. Importantly, etomoxir was used at 3 μM, a concentration that blocks CPT1 without off-target effects on the adenine nucleotide translocase or respiratory complex I (Fig 1B; Divakaruni *et al*, 2018; Raud *et al*, 2018). To quantify mitochondrial fragmentation and elongation, each experimental group was also analyzed for Aspect Ratio (AR) by confocal imaging of mitochondria stained with TMRE. AR is defined as the measurement of the long axis over the short axis, where an AR value of 2 would indicate a mitochondrion that is two times longer than it is wide. As such, a higher AR value would indicate a more elongated mitochondrion, while a smaller AR value is indicative of mitochondrial fragmentation (Molina *et al*, 2009). As expected, AR was reduced upon forced fragmentation in mi*MFN2* cells compared to cells transduced with control viruses, while AR was increased in cells expressing DN-DRP1, consistent with hyper-elongated mitochondrial morphology (Fig 1B). Remarkably, FAO was increased in mi*MFN2* cells but reduced in cells expressing DN-DRP1 compared to controls, suggesting a positive association between mitochondrial fragmentation and FAO capacity (Fig 1B). Importantly, these findings indicate that acute manipulation of mitochondrial fragmentation and elongation changes FAO, pointing to FAO as a downstream effector of mitochondrial morphology. In parallel studies, we found that increased FAO in HepG2 cultured under excess nutrient was accompanied by reduced AR values (increased fragmentation) compared to control cells (Fig 1Bi). Remarkably, inhibition of fragmentation or forced elongation under excess nutrient conditions blunted FAO (Fig 1Bii, DN-DRP1 + excess nutrient). We then integrated all the AR and FAO data points obtained from cells with fragmented mitochondria and those with elongated mitochondria across all tested conditions above and found that the correlation between mitochondrial architecture and the capacity to utilize fatty acids spans across a large spectrum of architectural changes ($R^2 = 0.6513$, Figs 1B and C, and EV1A, Table EV1). FAO can be controlled at several levels, including fatty acid import and metabolism, as well as oxidative phosphorylation. If changes to mitochondrial architecture limit FAO by reducing oxidative phosphorylation, we would expect that respiration fueled by succinate will be reduced to similar levels as respiration fueled by FAO. However, in all conditions tested, we find that the OCR values under succinate are at least twofold higher than under palmitate, indicating that oxidative phosphorylation is not limiting FAO at any of the mitochondrial architectures tested (Fig EV1B).

To further corroborate the above findings, we undertook a second independent approach to quantify FAO, namely metabolic tracing using [U-$^{13}C_{16}$] palmitate in both HepG2 and primary hepatocytes (Fig 1D). FAO was assessed by quantifying ^{13}C enrichment from palmitate-derived acetyl-CoA into citrate (m + 2 citrate, Fig 1D). The abundance of m + 2 citrate was higher in mi*MFN2* and *MFN1* KO cells compared to controls, consistent with higher FAO capacity upon increased mitochondrial fragmentation (Figs 1E and F, and EV1C). Conversely, forced mitochondrial elongation via overexpression of MFN1 (MFN1OE), siRNA-mediated knockdown of DRP1 (si*DRP1*), or expression of DN-DRP1 resulted in diminished m + 2 citrate, consistent with lower FAO capacity (Fig 1G–I). Beyond corroborating the respirometry data in Fig 1B and C, these results also confirm that the influence of mitochondrial fragmentation and elongation on FAO extends beyond HepG2 cells and is preserved in primary hepatocytes (Fig 1G–I). In addition, the observation that MFN1 OE and si*DRP1* phenocopy the effects of DN-DRP1 on FAO, further confirms that prevention of mitochondrial fragmentation by inhibiting fission blunts FAO. Of note, changes in FAO following the above alterations in mitochondrial morphology occur in the absence of changes in expression levels of FAO enzymes such as carnitine O-palmitoyltransferase 1 (CPT1) (Fig EV1D–F). Collectively, these data indicate that mitochondrial fragmentation drives FAO, potentially as a cause-and-effect relationship.

Mitochondrial fragmentation stimulates gluconeogenesis in primary hepatocytes

FAO plays an important role in adaptive responses to fasting, where the liver produces and secretes glucose as a fuel source for other tissues (hepatic glucose production). Specifically, FAO stimulates gluconeogenesis during fasting via provision of carbon substrates, reducing equivalents and ATP (McCune *et al*, 1981; Baron *et al*, 1989; Lam *et al*, 2003a,b; Rui, 2014; Satapati *et al*, 2015). Accordingly, we predicted that mitochondrial fragmentation may be normally required for glucose secretion in primary hepatocytes exposed to gluconeogenic substrates such as lactate and pyruvate. Forced mitochondrial elongation in primary hepatocytes via si*DRP1* or DN-DRP1 expression resulted in 30–40% reduction of gluconeogenesis in response to lactate and pyruvate compared to control hepatocytes (Fig 1J and K). To independently corroborate these findings and further rule out the possibility that the effect on gluconeogenesis is limited only to reduced fission, we also assessed the outcome of mitochondrial elongation in similar assays. Primary hepatocytes subjected to MFN1 or 2 overexpression showed a significant diminution of the gluconeogenic response to lactate and pyruvate (Fig 1L and M). This is consistent with diminished FAO under conditions where mitochondrial architecture is hyperelongated (Fig 1G–I and Table EV2).

Increased mitochondrial fragmentation in pancreatic β-cells stimulates FAO and alters their insulin secretory response to glucose and fatty acids

The above findings motivated further examination of the interplay between mitochondrial fragmentation and cellular response to lipids in additional contexts. To this end, we chose pancreatic islet β-cells as a cellular system, where mitochondrial fragmentation has been well documented to be the result of nutrient overload in prediabetic, obese, and in diabetic models both *in vitro* and *in vivo* (Molina *et al*, 2009; Herńandez-Alvarez *et al*, 2010; Supale *et al*, 2012; Masini *et al*, 2017). β-cells secrete insulin in response to stimulatory glucose concentrations (> 10 mM), thereby maintaining systemic euglycemia. However, under chronic nutrient overload as in obesity and metabolic syndrome, β-cells secrete insulin in response to fatty acids, even at low glucose concentrations, contributing to elevated insulin secretion at nonstimulatory conditions that is characteristic of the obese prediabetic state (Nolan *et al*, 2006a; Merrins *et al*, 2022). The development of high insulin secretion at nonstimulatory glucose levels, hereafter referred to as "hypersecretion," is associated with an increase in β-cell fatty acid utilization (Nolan *et al*, 2006a; Fex *et al*, 2007). However, the mechanism underlying

 © 2023 The Authors

increased fatty acid utilization and the role of mitochondrial fragmentation in increased fatty acid utilization as well as in the development of insulin hypersecretion is not clear.

To test if fragmentation alone can induce hypersecretion, we first determined the molecular mechanism underlying diet-induced mitochondrial fragmentation in the β-cell. Previous studies by Molina *et al* (2009) indicated that excess nutrient environment directly affects mitochondrial fusion, suggesting the expression levels of mitochondrial fusion proteins and/or function may be altered. Reduction in MFN2 levels has been demonstrated in muscle and liver of diet-induced obesity models (Gan *et al*, 2013; Gasier *et al*, 2020), prompting us to examine β-cell MFN2 levels in islets isolated from two obesity models: high-fat diet (HFD)-fed mice and Zucker rats. In both models, we observed reduction in MFN2 compared to control animals (Fig EV2A and B). Insulin hypersecretion in these models is linked to lipid excess in obesity (Erion *et al*, 2015; Taddeo *et al*, 2020). β-cells from obese animals also have increased FAO and secrete insulin in response to fatty acids (Nolan *et al*, 2006a). Additionally, diabetes and obesity induce mitochondrial fragmentation in β-cells (Supale *et al*, 2012; Gao *et al*, 2014b; Masini *et al*, 2017). However, whether these diet-driven changes in β-cells, namely mitochondrial fragmentation, insulin hypersecretion, increased FAO, and insulin release in response to fatty acids are mechanistically and functionally interrelated is not fully understood.

We have previously modeled the effect of obesity on mitochondrial architecture in β-cells by chronic exposure of a clonal β-cell line (INS-1 cells) to high glucose and palmitate *in vitro*, and shown that it involves inhibition of mitochondrial fusion and induction of fragmentation (Molina *et al*, 2009; Stiles & Shirihai, 2012). This is also evident from significant diminution of AR (Fig 2A and B) under these conditions and is accompanied by increased FAO, as revealed by etomoxir-sensitive respiration (Fig 2C), as well as diminished MFN2 protein levels (Fig 2D). To determine if insulin hypersecretion and the increase in FAO under excess nutrient conditions are a direct consequence of mitochondrial fragmentation in primary β-cells, we generated a β-cell-specific *Mfn2* knockout mouse model (β-Mfn2KO) (Fig EV2C). *Mfn2* deletion is sufficient to cause a fragmented mitochondrial network in β-cells as assessed by confocal microscopy of TMRE-stained β-Mfn2KO islets that were additionally engineered to express mito-PAGFP under the control of an insulin promotor for specific identification of β-cells within islets (Fig 2E). We also found that FAO in β-Mfn2KO islets is significantly higher compared to control islets from LoxP mice, as revealed by $^{14}CO_2$ production from ^{14}C-labeled palmitate (Fig 2F). Remarkably, *Mfn2* deletion in β-cells also leads to increased insulin secretion in islets cultured at nonstimulatory glucose concentrations (3 mM) but did not alter insulin secretion in response to stimulatory glucose concentrations (15 mM; Fig 2G). When cultured in the presence of palmitate at nonstimulatory glucose concentrations, β-Mfn2KO islets also mounted a larger insulin secretion response compared to LoxP control islets (Fig 2H). These changes in β-cell secretory function were not associated with alterations in islet architecture and composition as evident from comparable total insulin content and the ratio of β- to α-cells in β-Mfn2KO and LoxP control islets (Fig EV2D and E). Moreover, a similar increase in basal insulin secretion at 3 mM glucose was also observed in islet perifusion studies of β-Mfn2KO islet (Fig EV2F).

The finding that *Mfn2* deletion in β-cells is sufficient to recapitulate features that have been previously thought unique to islets from obese animals (Nolan *et al*, 2006b; Fex *et al*, 2007), namely increased FAO and insulin secretion in response to palmitate even at nonstimulatory glucose concentrations, is both remarkable and unexpected. Interestingly, these alterations occur in islets derived from β-Mfn2KO mice on normal chow diet, suggesting that reduced mitochondrial fusion or increased fragmentation itself could be an underlying mechanism for increased β-cell sensitivity to fatty acids and insulin hypersecretion. A critical test of this hypothesis is

Figure 2. Mitochondrial fragmentation upon *Mfn2* deletion increases β-cell sensitivity to fatty acid.

- A Representative confocal images of mitochondria in INS-1 cells cultured under control or excess nutrient conditions (20 mM glucose and 0.4 mM palmitate) and subsequently stained with MitoTracker Green (green). Scale bar: 10 μm.
- B Quantification of mitochondrial length by aspect ratio in INS-1 cells under control and excess nutrient conditions as in (A). Data represent means ± SEM, $n = 219$ cells from control and $n = 148$ cells from excess nutrient condition were analyzed, *t*-test. ***$P < 0.001$.
- C FAO assessed by OCR stimulated by palmitoyl-CoA and carnitine in permeabilized INS-1 in the presence of ADP. Data represent means ± SEM, $n = 4$ independent biological replicates (six technical replicates each *n*), *t*-test. ***$P < 0.001$.
- D MFN2 protein levels in INS-1 cells cultured under control and excess nutrient conditions as in (A) assessed by western blotting. Vinculin serves as loading control. Protein lysates from $n = 3$ experiments were analyzed and quantified by ImageJ, data represent means ± SEM, *t*-test. ***$P < 0.001$.
- E Representative confocal images of β-cells (GFP positive) and non-β-cells (GFP negative) in dispersed islets from LoxP control and β-Mfn2KO mice. Mitochondria are labeled with TMRE (red) and mito-PAGFP (green) driven by the insulin promoter. Mitochondrial fragmentation is only evident in the β-Mfn2KO samples. Scale bar: 10 μm.
- F FAO (β-oxidation) in LoxP control and β-Mfn2KO islets assessed by ^{14}C-CO_2 production from ^{14}C-U-labeled palmitate. Data represent means ± SEM, $n = 4$ mice per genotype with 100 islets analyzed per condition in each experiment. Data represent means ± SEM, *t*-test. **$P < 0.01$.
- G Averaged insulin secretion values at nonstimulatory (3 mM) and stimulatory (15 mM) glucose concentrations in LoxP control and β-Mfn2KO islets derived from $n = 4$ and $n = 5$ mice, respectively. Data represent means ± SEM, *t*-test. *$P < 0.05$. n.s, nonsignificant.
- H Fatty acid-stimulated insulin secretion in LoxP control and β-Mfn2KO islets derived from $n = 4$ and $n = 5$ mice and stimulated with 0.4 mM palmitate and 3 mM glucose. Data represent means ± SEM, *t*-test. *$P < 0.05$.
- I Confocal images of mitochondria in dispersed islets from CHOW- or HFD-fed mice with or without the MFN2 agonist peptide MFN2-TAT-P374-384 (P374-384) to increase mitochondrial fusion (connectivity). Mitochondria are stained with GPR75 and connectivity and length are presented as maximum projections. Scale bar: 10 μm.
- J Quantification and distribution of mitochondrial length in response to vehicle or P374-384 treatment of all islets derived from $n = 4$ CHOW- or HFD-fed mice. Vehicle data are from $n = 39$ CHOW islets and $n = 55$ HFD islets. P374-384 data are from $n = 55$ CHOW islets and $n = 31$ HFD islets. Data in the panel on the left represent means ± SEM, *t*-test. ***$P < 0.001$.
- K Insulin secretion at nonstimulatory glucose concentrations in islets derived from $n = 4$ CHOW- or HFD-fed wild-type mice and subsequently treated with P374-384. Data represent means ± SEM with 2–3 islets analyzed per mouse in each treatment group, *t*-test. *$P < 0.05$.

▶

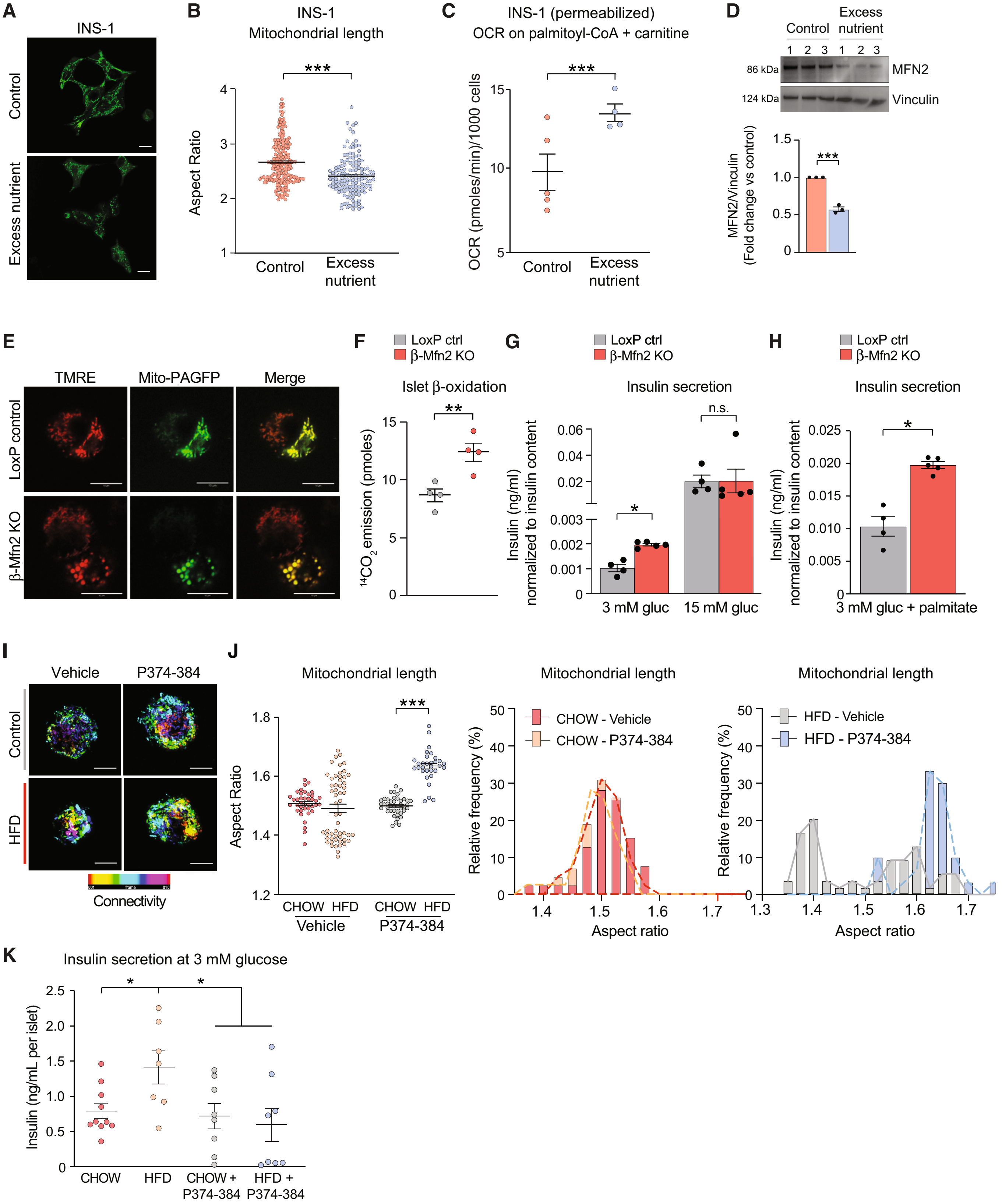

Figure 2.

 © 2023 The Authors

whether rescue of MFN2 function is sufficient to restore the normal secretory behavior of islets under HFD, where MFN2 protein levels are normally diminished (Fig EV2A). To this end, we utilized a cell-permeable peptide agonist of MFN2 (MFN2-TAT P374-384). This agonist acts on MFN2 Ser374 phosphorylation site to promote MFN1:MFN2 or MFN2:MFN2 dimer–dimer interactions, thereby producing mitochondrial tethering and evoking fusion (Rocha *et al*, 2018). As predicted, MFN2-TAT P374-384 increased mitochondrial connectivity and AR values in dispersed islet cells from HFD-fed mice consistent with increased mitochondrial fusion (Figs 2I and J). To further control for peptide specificity and on-target activity, we tested its effect in *Mfn2* knockout (MFN2 KO) and *Mfn1/2* double-knockout cells (MFN1/2 DKO). Treatment with MFN2-TAT P374-384 only increased mitochondrial length in MFN2 KO cells and not in WT or MFN1/2 DKO cells as measured by increased mitochondrial AR values (Fig EV3A). To verify enhanced mitochondrial length is due to stimulated fusion, we assessed for luminal continuity of the mitochondrial matrix via photo-activation of GFP in MFN2 KO cells with and without MFN2-TAT P374-384 (Fig EV3B). We found treatment with the peptide agonist leads to a right shift in mitochondrial area (Fig EV3C). This suggests that MFN2-TAT P374-384 requires an endogenous MFN1 or MFN2 and acts on MFN2 phosphorylation sites to promote mitochondrial fusion (Rocha *et al*, 2018). Importantly, the MFN2 agonist peptide reversed insulin hypersecretion at 3 mM glucose in islets from HFD-fed mice (Fig 2K; HFD + P374-384 versus HFD).

Taken together, our complementary approaches to loss- and gain-of-function modulation of MFN2 using genetic and peptide agonist strategies link mitochondrial fragmentation in primary β-cells to their insulin secretory function and their response to fatty acids. These findings also provide yet another example where increased FA utilization induced by fragmentation influences cell physiology.

Mitochondrial architecture determines fuel patterns in metabolic subtypes of diffuse large B-Cell lymphomas (DLBCLs)

Numerous studies point to complex metabolic circuitries in tumors beyond aerobic glycolysis, including capacities to utilize a variety of fuel sources in tumor type- and context-specific manner. These fuel patterns can impact tumor growth, invasiveness, as well as response and/or resistance to therapy (García-Ruiz *et al*, 2015; Vander Heiden & DeBerardinis, 2017; Faubert *et al*, 2020; Broadfield *et al*, 2021). The finding that fragmentation drives FAO raises the question whether this connection is relevant to metabolic specialization and fuel decisions in cancer cells, especially those prone to FAO. Within this context, we chose to focus on metabolic subtypes of diffuse large B-cell lymphomas (DLBCLs) with distinct fuel utilization patterns, which we have previously characterized (Caro *et al*, 2012; Norberg *et al*, 2017). Specifically, we showed that OxPhos-DLBCLs display elevated FAO, while non-OxPhos B-cell receptor-dependent DLBCLs (BCR-DLBCLs) have greater glycolytic flux (typical of Warburg-type tumors) and are sensitive to inhibition of glycolysis. In comparison, OxPhos-DLBCLs derive pro-proliferative and pro-survival benefits from fatty acids and are resistant to inhibition of BCR signaling or glycolysis (Caro *et al*, 2012; Chen *et al*, 2013; Norberg *et al*, 2017).

Analysis of multiple independent patient-derived OxPhos- and BCR-DLBCL indicated that, compared to BCR-DLBCLs, OxPhos-DLBCLs have fragmented mitochondria, as revealed by the cross-sectional area of individual mitochondria in cells stained with MitoTracker Green, and quantification of mitochondria with a form factor (FF) < 2 (Figs 3A–C and EV4). Mitochondrial FF is a readout for mitochondrial branching, where reduced FF would suggest the mitochondrion is more spherical, while greater FF values would suggest highly interconnected mitochondria (Cribbs & Strack, 2009; Sisalli *et al*, 2020). Mitochondria in DLBCL cells were classified into short (FF $\leq$ 2), intermediate ($2 \leq$ FF ≤ 4), and long (FF > 4) morphologies. Based on these criteria, we found that OxPhos-DLBCLs have significantly higher frequency of fragmented mitochondria compared to BCR-DLBCLs, where elongated mitochondria are more frequent (Fig 3C and D).

The differences in mitochondrial morphology in metabolic subtypes of DLBCLs match their FAO capacity with higher fragmentation in FAO prone OxPhos-DLBCLs (Figs 3A–E and EV4). Importantly, mitochondrial fragmentation facilitates FAO in OxPhos-DLBCLs as evident from metabolic tracing showing that forced mitochondrial elongation in OxPhos-DLBCLs via MFN2 OE or DN-DRP1 significantly diminishes m + 2 citrate from [U-$^{13}C_{16}$] palmitate relative to OxPhos-

Figure 3. Mitochondrial fragmentation is necessary and sufficient to enhance FAO in DLBCLs.

A Representative 3D reconstructed images of OxPhos-DLBCL (Toldeo and Pfeiffer) and BCR-DLBCL (DHL-6 and DHL-4) cell lines labeled with MitoTracker Green. Scale bar: 10 μm.

B Average area of individual mitochondria in OxPhos- (red) and BCR-DLBCL (blue) cell lines labeled as in (A). Data represent means ± SEM with $n = 10$–12 cells analyzed per cell line, unpaired *t*-test with Welch's correction. ***$P < 0.001$.

C Frequency of fragmented mitochondria as defined by a form factor (FF) of < 2 in cells from the same experiments as in (A) and (B). Data represent means ± SEM with $n = 10$–12 cells analyzed per cell line, unpaired *t*-test with Welch's correction. ***$P < 0.001$.

D Frequency of elongated mitochondria as defined by a form factor (FF) of > 4 in cells from the same experiments as above (A) and (B). Data represent means ± SEM with $n = 10$–12 cells analyzed per cell line, unpaired *t*-test with Welch's correction. ***$P < 0.001$.

E Palmitate-induced OCR in DLBCL cell lines. Individual data points are from $n = 5$ and $n = 3$ independent cell lines per OxPhos- and BCR-DLBCL subtypes, respectively. Data represent means ± SEM, *t*-test. *$P < 0.05$.

F–H Fractional abundance of m + 2 citrate from [U-$^{13}C_{16}$] palmitate in OxPhos- and BCDR-DLBCL subtypes following forced mitochondrial elongation in response to MFN2 OE (F) or DN-DRP1 (G) and following forced mitochondrial fragmentation by *MFN2* knockdown (H). Data represent means ± SEM and are cumulative data points from multiple individual experiments using independent cell lines for each DLBCL subtype as follows: $n = 6$ independent biological replicates in (F) using two independent cell lines per each DLBCL subtype, $n = 21$ independent biological replicates in (G) using five independent OxPhos- and four independent BCR-DLBCL cell lines, $n = 3$–6 independent biological replicates in (H) using three independent OxPhos- and two independent BCR-DLBCL cell lines, *t*-test. *$P < 0.05$, **$P < 0.01$, n.s, nonsignificant.

I, J Cell viability in OxPhos- and BCR-DLBCLs expressing DN-DRP1 (I) or MFN2 (J). Data represent means ± SEM from four independent OxPhos-DLBCL cell lines in triplicate and 4–5 independent BCR-DLBCL cell lines in duplicate (I) or triplicate (J), *t*-test. ***$P < 0.001$, n.s, nonsignificant.

▶

© 2023 The Authors

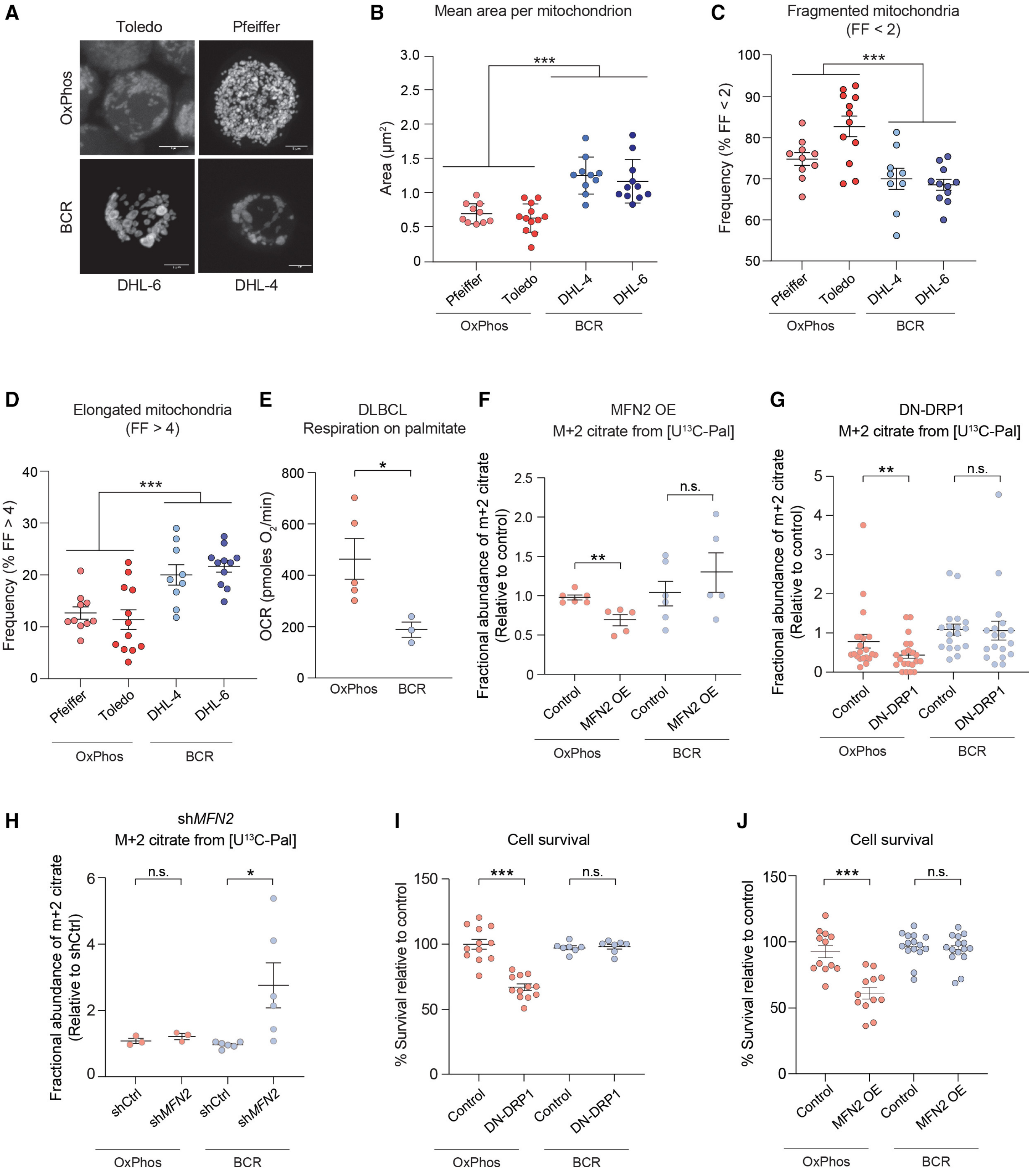

Figure 3.

DLBCLs transduced with control viruses (Fig 3F and G and Table EV2). In contrast, no differences in m + 2 citrate were observed in BCR-DLBCLs treated with control, MFN2 or DN-DRP1 viruses (Fig 3F and G). These observations indicate a specific requirement for fragmentation to support a key metabolic feature of OxPhos-DLBCLs, namely FAO. Conversely, forced mitochondrial fragmentation in BCR-DLBCLs upon shRNA-mediated *MFN2* knockdown (sh*MFN2*) stimulates enrichment of m + 2 citrate from [U-$^{13}C_{16}$] palmitate

 © 2023 The Authors

relative to control-infected BCR-DLBCLs (Fig 3H). Thus, inducing mitochondrial fragmentation is remarkably sufficient in rendering BCR-DLBCLs prone to FAO. We further tested the functional connection between mitochondrial fragmentation and FAO using cell survival as an independent readout based on published observations that FAO is selectively required for the survival of OxPhos-DLBCLs (Caro *et al*, 2012). Accordingly, we predicted that inhibition of mitochondrial fission over time would selectively reduce OxPhos-DLBCL survival as it blunts FAO. Indeed, longer term expression of DN-DRP1 or MFN2 selectively compromises the survival of OxPhos- but not BCR-DLBCLs (Fig 3I and J). Overall, the finding that FAO can be inhibited or triggered in metabolic subtypes of DLBCLs by acute manipulation of mitochondrial fusion and fission provides novel insights into mitochondrial architecture as an underlying mechanism for fuel utilization in these cells.

The effect of mitochondrial architecture on FA utilization is selective for long-chain fatty acids and involves regulation of CPT1 activity

Changes in the FAO pathway in response to mitochondrial morphologic alterations can result from alterations in one or more steps. These can include FA activation upon conversion to fatty acyl-CoAs, transesterification to fatty acyl-carnitines by CPT1 for transport into the mitochondria, or successive removal of two-carbon units as acetyl-CoA to fuel the TCA cycle. Unlike long-chain FAs (LCFAs), short-chain FAs (SCFAs) cross the mitochondrial membranes independent of CPT1 and are directly activated in the matrix to undergo β-oxidation. To dissect which of the aforementioned steps in FAO are affected by mitochondrial fragmentation, we compared the incorporation of carbons derived from the LCFA palmitate and the SCFA hexanoate into citrate as a readout of LCFAO and SCFAO (Fig 4A). We reasoned that this comparison could initially help rule in or out steps that are common in oxidation of LCFAs and SCFAs. While forced mitochondrial elongation in OxPhos-DLBCLs via MFN2 OE or DN-DRP1 reduced palmitate oxidation relative to cells infected with control viruses, it did not alter hexanoate oxidation (Fig 4B and C). In BCR-DLBCLs, neither LCFAO nor SCFAO was affected by forced mitochondrial elongation (Fig EV5A and B). The selective effect of mitochondrial architecture on LCFAO versus SCFAO was also observed in primary hepatocytes, where forced elongation via DN-DRP1 or *siDRP1* led to diminution of LCFAO without altering SCFAO (Fig 4D and E and Table EV2). The finding that mitochondrial architecture selectively controls LCFAO but not SCFAO points to CPT1 as a potential biochemical mechanism connecting mitochondrial morphology to FAO since it is a key step distinguishing mitochondrial handling of LCFAs from that of SCFAs (McGarry & Brown, 1997).

CPT1 activity is sensitive to allosteric inhibition by malonyl-CoA, which lowers the affinity of the enzyme for its substrate carnitine (López-Viñas *et al*, 2007). In both primary hepatocytes and OxPhos-DLBCLs, mitochondrial hyper-elongation via DN-DRP1 rendered CPT1 more sensitive to malonyl-CoA inhibition (Fig 5A and B), which is consistent with reduced FAO in the same setting (Figs 1I and 3G). Of note, expression levels of CPT1 protein were not altered in response to DN-DRP1 or increasing malonyl-CoA concentrations, ruling out changes in CPT1 abundance as an explanation for altered CPT1 activity in these studies (Fig EV6A and B and Table EV2). Moreover, CoA produced in the presence of D-carnitine alone was utilized for background subtraction in these assays, thus, the above effects cannot be attributed to CoA produced independent of CPT1 (Fig EV6C and D). Conversely, reduced sensitivity to malonyl-CoA inhibition in the context of higher frequency of mitochondrial fragmentation would be consistent with increased CPT1 activity and FAO (Figs 1E and F, and 3H). Similarly, the connection between mitochondrial fragmentation and reduced CPT1 malonyl-CoA sensitivity is evident in INS-1 cells under excess nutrient conditions, where stimulation of OCR by palmitoyl-CoA and carnitine was less sensitive to malonyl-CoA inhibition (Fig 5C).

To further interrogate the hypothesis that the effect of mitochondrial architecture on CPT1 involves changes in CPT1 sensitivity to malonyl-CoA, we determined if pharmacological agents that prevent malonyl-CoA binding would reduce CPT1 sensitivity to mitochondrial architecture. To this end, we took advantage of C75 and baicalin, two CPT1 activators with different mechanisms of action (Fig 5D). C75 activates CPT1 by competing with malonyl-CoA for the same binding sites, thereby reversing malonyl-CoA inhibition (Thupari *et al*, 2002; Nicot *et al*, 2004). However, baicalin activates CPT1 by binding to the catalytic site (Dai *et al*, 2018). As such, baicalin cannot rescue CPT1 activity if malonyl-CoA is bound to the enzyme (Dai *et al*, 2018). Based on these distinct mechanisms of action, we predicted that C75 but not baicalin would reverse the inhibitory effect of mitochondrial hyperelongation on CPT1 activity and FAO. To this end, we first evaluated the capacity of C75 and baicalin to enhance FAO using [U-$^{13}C_{16}$] palmitate tracing assays and showed that these effects were abrogated in the presence of etomoxir, confirming the specificity of these reagents for CPT1 as a target (Fig EV6E). We also performed imaging studies to ensure mitochondrial morphology or membrane potential was not altered by C75 and baicalin (Fig EV6F and G). Consistent with the above prediction, treatment of primary hepatocytes overexpressing MFN2 with C75 rescued FAO in [U-$^{13}C_{16}$] palmitate tracing studies, whereas treatment with baicalin had no effect (Fig 5E and Table EV2). Similar results were also obtained in the context of DN-DRP1 (Fig 5F and Table EV2). Collectively, the combination of

Figure 4. Mitochondrial architecture specifically controls long-chain FAO.

A Schematic representation of an isotope tracing strategy to distinguish long-chain versus short-chain FAO [U-$^{13}C_{16}$] palmitate and [$^{13}C_1$] hexanoate, respectively.

B, C Fractional abundance of m + 2 citrate from [U-$^{13}C_{16}$] palmitate (LCFAO) compared with m + 1 citrate from [$^{13}C_1$] hexanoate (SCFAO) following forced mitochondrial elongation by MFN OE (B) or DN-DRP1 (C) in OxPhos-DLBCLs. Data represent means ± SEM and are cumulative data points from multiple individual experiments using independent cell lines as follows: n = 5–6 independent biological replicates in (B) using two independent OxPhos-DLBCL cell lines, n = 17–21 independent biological replicates in (C) using five independent OxPhos-DLBCL cell lines, t-test. $^{**}P < 0.01$, n.s, nonsignificant. The [U-$^{13}C_{16}$] palmitate arm of these experiments is the same as shown in Fig 3F and G.

D, E Fractional abundance of m + 2 citrate from [U-$^{13}C_{16}$] palmitate (LCFAO) compared with m + 1 citrate from [$^{13}C_1$] hexanoate (SCFAO) following forced mitochondrial elongation by DN-DRP1 (D) or *DRP1* knockdown (E) in primary hepatocytes. Data represent means ± SEM, n = 3 mice, t-test. $^{**}P < 0.01$, n.s, nonsignificant.

© 2023 The Authors

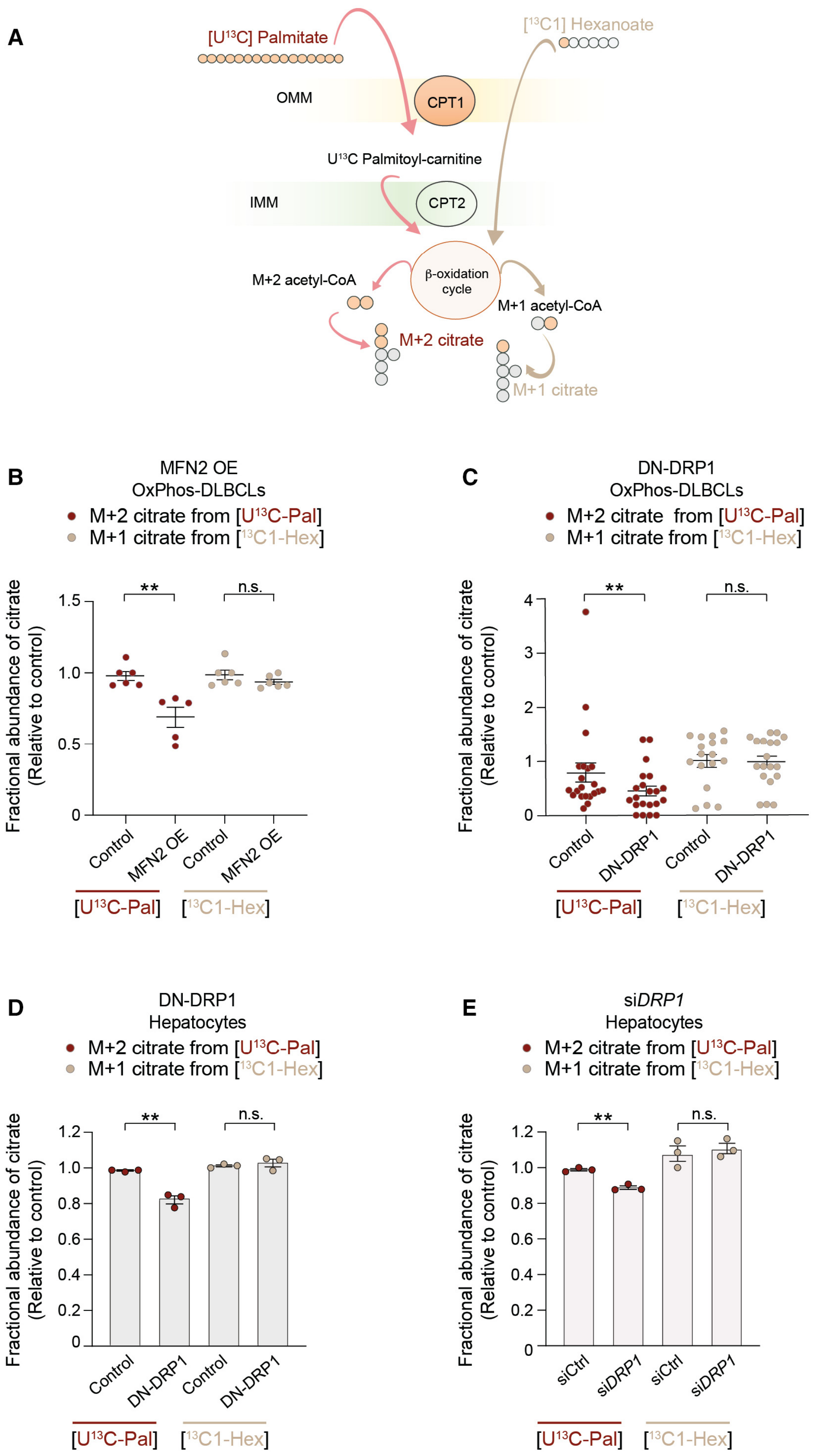

Figure 4.

© 2023 The Authors

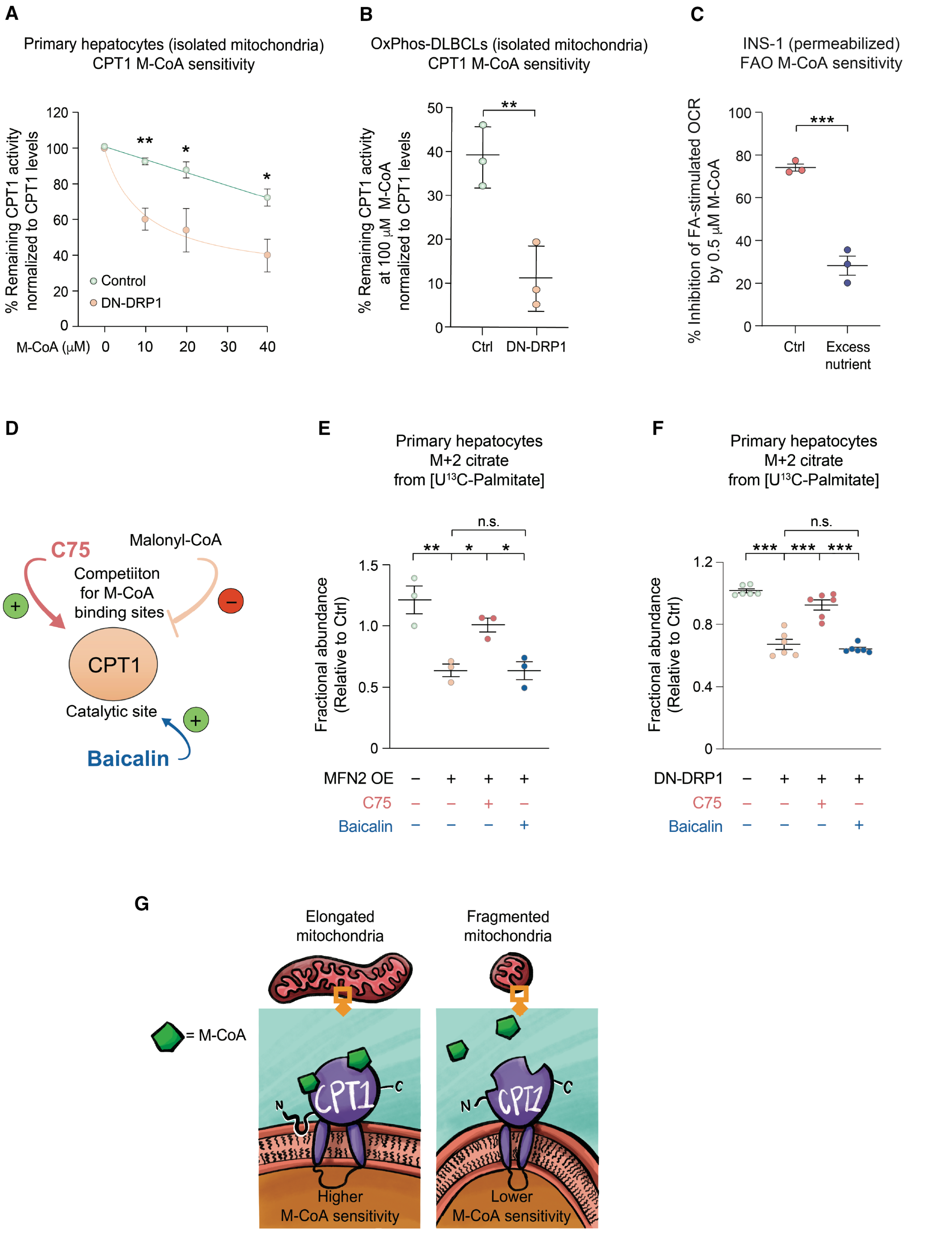
A
Primary hepatocytes (isolated mitochondria)
CPT1 M-CoA sensitivity
% Remaining CPT1 activity normalized to CPT1 levels
Control
DN-DRP1
M-CoA (μM)
B
OxPhos-DLBCLs (isolated mitochondria)
CPT1 M-CoA sensitivity
% Remaining CPT1 activity at 100 μM M-CoA normalized to CPT1 levels
Ctrl
DN-DRP1
C
INS-1 (permeabilized)
FAO M-CoA sensitivity
% Inhibition of FA-stimulated OCR by 0.5 μM M-CoA
Ctrl
Excess nutrient
D
C75
Malonyl-CoA
Competiiton for M-CoA binding sites
CPT1
Catalytic site
Baicalin
E
Primary hepatocytes
M+2 citrate
from [U13C-Palmitate]
Fractional abundance (Relative to Ctrl)
n.s.
MFN2 OE
C75
Baicalin
F
Primary hepatocytes
M+2 citrate
from [U13C-Palmitate]
Fractional abundance (Relative to Ctrl)
n.s.
DN-DRP1
C75
Baicalin
G
Elongated mitochondria
Fragmented mitochondria
= M-CoA
Higher M-CoA sensitivity
Lower M-CoA sensitivity

Figure 5.

Figure 5. Mitochondrial architecture controls LCFAO by regulating CPT1 sensitivity to Malonyl-CoA.

A CPT1 activity in the presence of increased malonyl-CoA (M-CoA) measured in mitochondria-enriched heavy membrane fractions isolated from control and DN-DRP1-expressing primary mouse hepatocytes. Enzyme activity was normalized to CPT1 protein levels in individual experiments. Data represent means ± SD, $n = 3$ independent biological replicates, *t*-test. $^*P < 0.05$, $^{**}P < 0.01$.

B CPT1 activity in the presence of 100 μM M-CoA measured in mitochondria-enriched heavy membrane fractions isolated from control and DN-DRP1-expressing OxPhos-DLBCLs. Enzyme activity was normalized to CPT1 protein levels in individual experiments. Data represent means ± SD, $n = 3$ individual experiments from one OxPhos-DLBCL cell line, *t*-test. $^{**}P < 0.01$.

C Inhibition of FA-dependent OCR in the presence of ADP and 0.5 μM M-CoA in permeabilized INS-1 cells cultured under control and excess nutrient conditions as in Fig 2A. Data represent means ± SEM, $n = 3$, *t*-test. $^{***}P < 0.001$.

D Schematic depicting two independent pharmacologic mechanisms of CPT1 activation; competition for M-CoA binding site by C75 and targeting the catalytic site by baicalin.

E, F Differential capacity of C75 and baicalin to rescue FAO in primary hepatocytes following forced mitochondrial elongation by MFN2 OE (E) or DN-DRP1 (F). The effect of these CPT1 activators on fractional abundance of m + 2 citrate was assessed in [U-$^{13}C_{16}$] palmitate tracing studies. Data represent means ± SEM, $n = 3$ independent biological replicates, one-way ANOVA. $^*P < 0.05$, $^{**}P < 0.01$, $^{***}P < 0.001$, n.s, nonsignificant.

G Schematic representation of proposed mechanism, whereby mitochondrial morphology regulates FAO through modulating CPT1 sensitivity to malonyl-CoA.

genetic manipulations, pharmacologic rescue studies, and FA tracing studies provides evidence for a previously unappreciated regulatory role of mitochondrial architecture in fuel metabolism that selectively converges on LCFAO via allosteric regulation of CPT1 (Fig 5G).

Discussion

Mitochondrial dynamics has been associated with bioenergetic adaptation to energy demand and supply (Liesa & Shirihai, 2013). In particular, mitochondrial fragmentation was reported in a variety of cellular models as a common response to exposure to excess lipids. However, whether fragmentation is an adaptive mechanism to lipid exposure, or a mere maladaptive dysfunction of mitochondrial dynamics has remained an open question. Here, we show in several cellular models that mitochondrial fragmentation favors FAO while mitochondrial elongation reduces FAO. Fragmentation-induced FAO is not only seen in response to excess nutrients and lipids but also upon acute genetic manipulation of mitochondrial fission and fusion proteins. Remarkably, these acute alterations in mitochondrial architecture only alter incorporation of LCFA-derived carbons into the TCA cycle without changing SCFA utilization, leading us to assess CPT1 activity as a downstream effector of mitochondrial morphology. We show CPT1 sensitivity to malonyl-CoA is diminished upon mitochondrial fragmentation, leading to increased LCFAO. In contrast, malonyl-CoA inhibition of CPT1 is enhanced following mitochondrial elongation, resulting in decreased LCFAO in a manner that can be selectively reversed by the CPT1 activator C75 but not baicalin. Taken together, these findings expand the role of mitochondrial dynamics beyond mitochondrial quality control by showing its functional relevance as a mechanism that regulates mitochondrial fuel preference for fatty acids, as well as adaptation to metabolic demand.

Quantitative assessment of the relationship between mitochondrial architecture and FAO

While specific perturbations in mitochondrial dynamics genes were shown to affect lipid utilization (Mahdaviani *et al*, 2017), the extent and range of morphologic states at which mitochondrial architecture correlates with the degree of lipid utilization has not been previously quantified. We initiated this study by quantifying mitochondrial FAO in a range of mitochondrial morphologies induced by different culture models, including lipid exposure as well as genetic perturbations of mitochondrial dynamic proteins. To directly evaluate the mitochondrial FAO capacity in respirometry assays, we permeabilized cells to control the levels of FA available to mitochondria. We find a remarkably linear association between FAO capacity and the extent of mitochondrial fragmentation. This relationship is maintained in conditions of extreme fragmentation as well as extreme elongation, supporting the conclusion that this is not limited to modulation of a single mitochondrial dynamic protein but rather it is driven by the architecture itself. The use of etomoxir as control in these assays enabled us to specifically focus on mitochondrial FAO and rule out contributions from peroxisomes, which also plays an important role in FAO (Reddy & Hashimoto, 2001; Violante *et al*, 2019). In addition, isotope tracing studies measuring incorporation of fatty acid-derived carbons into the TCA metabolite citrate in cells with fragmented versus elongated mitochondria further corroborate the conclusion that mitochondrial architecture influences mitochondrial FA utilization.

Regulation of FAO by mitochondrial architecture is relevant for cell type-specific biological functions

Our studies show that the relationship between mitochondrial architecture and FAO is not only conserved in multiple cell types from diverse tissue origins (hepatocytes, β-cells and DLBCLs) but is also functionally required for specific FAO-driven biologic responses relevant to each cell type. Such biologic responses include gluconeogenesis in hepatocytes, adaptive increases in β-cell insulin secretion in response to obesity and lipid excess, as well as survival of FAO-dependent cancer cells such as OxPhos-DLBCLs. The finding that these diverse cellular phenotypes are altered as a consequence of the effect of mitochondrial morphology on fatty acid utilization supports a central role for mitochondrial morphology as a basic biologic mechanism controlling FAO that was not fully appreciated.

We find that mitochondrial architecture specifically controls LCFAO without altering SCFAO in the above cell types. Thus, the metabolic phenotype associated with acute alterations of mitochondrial dynamics in these systems occurs in the absence of pleiotropic changes in mitochondrial fuel handling and respiratory chain function. As such, these experimental models provided a more tractable

 © 2023 The Authors

system to dissect the specific metabolic consequences of acute changes in mitochondrial dynamics. In comparison, this has been more challenging to tease apart in *in vivo* studies due to secondary phenotypes perhaps resulting from chronic imbalances in mitochondrial fusion/fission and large spectrum alternations in mitochondrial function (Sebastián *et al*, 2012; Wang *et al*, 2015; Kulkarni *et al*, 2016). For example, hepatic deletion of DRP1 in mice produces a complex phenotype that includes what appears to be secondary increases in FGF21 production (Wang *et al*, 2015). This is reminiscent of FGF21 induction upon activation of the integrated stress response in the liver (Xu *et al*, 2018). Moreover, liver-specific MFN2 KO mice show exaggerated hepatic glucose production (HGP) from pyruvate (Sebastián *et al*, 2012). This is consistent with our observations that mitochondrial fragmentation is required for proper regulation of gluconeogenesis in primary hepatocytes. However, hepatic loss of MFN2 also leads to mitochondrial dysfunctions, including reduced respiratory chain activity, diminished CoQ levels, as well as increased ROS and ER stress (Sebastián *et al*, 2012, 2017). It is also possible that changes in ER calcium homeostasis in the absence of MFN2 may, in part, account for some of these observations. For these reasons, the precise cause of altered HGP in these mice could not be definitively deduced. In comparison, liver-specific MFN1 KO mice did not show evidence of hepatic ER stress and had diminished whole body respiratory exchange ratio and reduced acyl-carnitines, signifying preferential usage of lipids as an energy source (Kulkarni *et al*, 2016). This is consistent with our data in multiple different cell types that increased fragmentation/reduced fusion enhances FAO.

We and others have shown that mitochondria fragment in response to excess fat and nutrient overload, including HFD treatment (Kuznetsov *et al*, 2004; Molina *et al*, 2009). Using the β-cell as a model, we now provide multiple lines of evidence that mitochondrial fragmentation is functionally required for adaptive responses to HFD such as increased FAO and stimulation of insulin secretion in response to fatty acids. Our data also suggest that diminished MFN2 expression in response to HFD is a mechanism linking dietary changes to mitochondrial fragmentation. This provides another example, where physiological mitochondrial fragmentation plays a specific adaptive role by facilitating cellular responses to lipid accumulation.

Our findings further point to a surprisingly specific requirement for mitochondrial fragmentation in the survival of certain cancer cells and metabolic specialization distinct from Warburg-type glycolytic metabolism. Changes in mitochondrial architecture have been linked to tumor progression, metastasis, and therapeutic resistance in different cancer types (Trotta & Chipuk, 2017; Boulton & Caino, 2022). Indeed, oncogenic signaling pathways can alter the expression and/or posttranslational modifications of mitochondrial dynamics proteins (Serasinghe *et al*, 2015; Malhotra *et al*, 2016; Nagdas *et al*, 2019; Chauhan *et al*, 2020). However, the specific mechanistic roles of altered mitochondrial architecture in tumor processes remain enigmatic and are likely to be context specific. In some studies fragmentation was associated with a shift to glycolysis, while in others, fragmentation was found to stimulate mitochondrial FA utilization, but underlying mechanisms that directly and causally explain these phenotypes were unclear (Nagdas *et al*, 2019; Liang *et al*, 2020; Song *et al*, 2021). Notably, several mitochondrial dynamics proteins have cellular roles beyond, or independent of, regulation of mitochondrial architecture (Itoh *et al*, 2019; Casellas-Díaz *et al*, 2021). As such, whether mitochondrial architecture *per se* regulates tumor processes and metabolic profiles requires investigating the outcome of loss- and gain-of-function studies of both fission and fusion proteins coupled with detailed investigation of specific fuel patterns. For this reason, we used independent approaches to examine the relevance of mitochondrial morphology by manipulating DRP1 or MFN2 in DLBCL subtypes. Furthermore, our data not only show that fragmentation dictates preference for fatty acids in OxPhos-DLBCLs, but that forced fragmentation in glycolytic BCR-DLBCLs is sufficient to endow these cells with FAO capacity. The latter observation is especially intriguing because FAO provides survival benefits to DLBCLs, independent of BCR (Caro *et al*, 2012). As such, mitochondrial fragmentation and FAO may be a potential mechanism of resistance to the inhibition of the BCR pathway in BCR-DLBCL. If true, this will have translational implications given that small molecule inhibitors of the BCR signaling axis are currently under clinical evaluation for DLBCL therapy. Regardless, our data link mitochondrial morphology not only to fuel patterns but also to fuel flexibility in DLBCL subtypes.

The above observations do not exclude scenarios where mitochondria hyperfuse in response to cellular stress or cellular energy deficit, and loss of fusion results in mitochondrial dysfunction (Chen *et al*, 2005; Tondera *et al*, 2009; Gomes *et al*, 2011). Although at first glance these results appear contradictory to ours, they rather reflect mitochondrial morphologic flexibility and its participation in cellular stress responses. For example, Tondera *et al* (2009) reported that stress-induced mitochondrial hyperfusion promotes cell survival. However, this can be explained by the need to maintain the long form of the inner mitochondrial membrane fusion GTPase OPA1 (L-OPA1) in this setting with concomitant reduction in the activity of the OMA1 protease, which can cleave OPA1 and subsequently trigger the mitochondrial integrated stress response (ISR). Another study examined the effect of mitochondrial hyperfusion and ISR hyperactivation in the context of nonalcoholic steatohepatitis (NASH) and showed that steatosis worsens upon mitochondrial elongation (Steffen *et al*, 2022). This was linked to impaired complex I activity necessary for FAO and accumulation of nonesterified fatty acids (Steffen *et al*, 2022). The studies by Tondera *et al* and Steffen *et al* are but two examples in further support of our findings that the dynamic adaptation of mitochondrial morphology, whether hyperfusion or forced fragmentation, play an integral role in metabolic flexibility and cell survival in a context-specific manner.

Mitochondrial architecture controls cellular fuel preference for LCFAs by modulating CPT1 malonyl-CoA sensitivity

Our data indicate that acute manipulation of mitochondrial dynamics proteins selectively alters LCFAO capacity that can be mechanistically explained by changes in the sensitivity of CPT1 to malonyl-CoA. Malonyl-CoA and carnitine bind to structurally distinct sites in CPT1 with an inverse relationship between the IC50 for malonyl-CoA inhibition and the binding affinity for carnitine (López-Viñas *et al*, 2007). How might mitochondrial membrane architecture modulate the effect of malonyl-CoA on CPT1? Previous NMR analyses of the malonyl-CoA-sensitive N-terminal domain (NTD) of CPT1A reconstituted in micellar membranes showed that this domain transitions between two conformations depending on the micelle curvature (Rao *et al*, 2011). The conformation supported by increased micellar

© 2023 The Authors

curvature is less sensitive to malonyl-CoA and the ratio of the two NTD conformers determines the overall malonyl-CoA sensitivity of the enzyme (Rao *et al*, 2011). Consistent with this model, molecular dynamics simulations suggested that a curved lipid bilayer supports the interaction between the NTD and the C-terminal domain of CPT1 by changing the orientation of CPT1's first transmembrane domain, ultimately resulting in enzyme activation (Frigini *et al*, 2020). In contrast, in a planar (less curved) lipid bilayer, NTD adopts a different conformation and contacts the outer membrane instead, leading to enzyme inactivation (Frigini *et al*, 2020). Such a scenario would be consistent with the published effect of DRP1 oligomerization in curving and restricting artificial membrane tubules (Ugarte-Uribe *et al*, 2018). Additional studies are required to assess how this model extends to CPT1 regulation in native mitochondrial membrane within cells. This model is especially attractive because it links the capacity of CPT1 to sense malonyl-CoA to the membrane lipid composition/fluidity and the membrane microenvironment, which are known to respond to metabolic and dietary states (Zammit *et al*, 1998; Faye *et al*, 2005; Aoun *et al*, 2012).

It is important to note that the above model does not rule out additional mechanisms linking CPT1 activity to membrane morphology. These include mitochondrial contact sites with other organelles such as ER and lipid droplets. ER-mitochondrial contact sites have been shown to mark the site of DRP1 recruitment and fission while providing a microdomain rich in signaling (Friedman *et al*, 2011; Herrera-Cruz & Simmen, 2017). Future studies are required to determine if and how CPT1 regulation by mitochondrial architecture is coordinately influenced by ER contact sites. In addition, the broader role of mitochondrial dynamics in lipid metabolism such as lipid utilization and lipid storage, including lipid droplet homeostasis, awaits further investigation (Mahdaviani *et al*, 2017; Song *et al*, 2021). Within this context, we have previously shown differential fuel selectivity in mitochondria associated with lipid droplets (peridroplet mitochondria) versus cytoplasmic mitochondria in brown adipose tissue (Benador *et al*, 2018). Specifically, peridroplet mitochondria tend to be more elongated with superior capacity to oxidize pyruvate but reduced capacity for FAO. Peridroplet mitochondria also synthesize triglycerides, leading to expansion of lipid droplets. In contrast, cytoplasmic mitochondria are more fragmented and have enhanced FAO capacity. In light of the findings in this study, it is possible that differential CPT1 regulation in the two types of mitochondria underlies the differences in fuel handling between cytoplasmic and peridroplet mitochondria.

In summary, our studies provide evidence that mitochondrial architecture can selectively affect mitochondrial fuel utilization and metabolic processes associated with LCFAO through modulation of CPT1 sensitivity to malonyl-CoA. These findings highlight a hitherto unappreciated role for mitochondrial fragmentation in basic metabolic regulation beyond its well-known relevance in clearance of damaged mitochondria through mitophagy.

Materials and Methods

Animals

The generation of Mfn2-loxP mice has been previously described (Chen *et al*, 2007). B6. Cg-Tg (Ins2-cre) 25 Mgn/J mice (RIP-Cre) were obtained from Jackson Laboratory (Bar Harbor, ME) and crossed to homozygous Mfn2-loxP to generate F1, double heterozygous mice. F1 mice were then crossed to the original Mfn2-loxP mice to generate the β-Mfn2KO mice.

For high-fat diet studies, 9-week-old C57BL/6J male mice were fed a custom diet containing 40% fat, 20% fructose, and 2% cholesterol (Research Diets [D09100310]) or a control chow diet (PicoLab Rodent Diet 205053) for 14–19 weeks. All animal care was in accordance with National Institutes of Health guidelines, University of California, Los Angeles (UCLA), and Dana-Farber Cancer Institute Institutional Animal Care and Use Committee.

Cell culture

Primary mouse hepatocytes were isolated and cultured as we have previously reported (Giménez-Cassina *et al*, 2014; Lane *et al*, 2019). Primary mouse islets were isolated from 11 to 16-week-old male C57BL/6J mice (Jackson Labs, Bar Harbor, ME) and cultured as previously published (Wikstrom *et al*, 2012; Taddeo *et al*, 2018).

Clonal HepG2 cells were cultured in low glucose (5 mM) Dulbecco's Modified Eagle Medium (Ref#31600-026) supplemented with 10% FBS, 50 U/ml penicillin, and 5 mM HEPES buffer. For excess nutrient conditions, cells were incubated for 24 h in high glucose (25 mM) Dulbecco's Modified Eagle Medium (Ref#12100-038) supplemented with 2% FBS, 50 U/ml penicillin, 5 mM HEPES buffer, and 250 μM 4:1 palmitate: BSA. MFN1 KO HepG2 cells were a generous gift from Dr. György Hajnóczky.

Clonal INS-1 cells were cultured in RPMI-1640 media as described previously (Molina *et al*, 2009; Wikstrom *et al*, 2012). For excess nutrient conditions, cells were incubated for 24 h in 20 mM glucose RPMI-1640 media supplemented with 300 μM 4:1 palmitate: BSA as described previously (Molina *et al*, 2009; Taddeo *et al*, 2020).

DLBCL cell lines (Pfeiffer, Toledo, Ly4, K422, DHL2, DHL-4, DHL-6, Ly1, U2932 and HBL-1) and their genetic classifications as OxPhos- or BCR-DLBCLs have been described previously and were cultured as we have previously reported (Caro *et al*, 2012; Norberg *et al*, 2017).

Wild-type, Mfn1 null (CRL-2992), Mfn2 null (CRL-2993), and Mfn1/Mfn2 double knockout MEFs (CRL-2994) were a generous gift from Dr. Gerald Dorn W II (Chen *et al*, 2003; Franco *et al*, 2016). MEFs were maintained in DMEM containing 4.5 g/l glucose supplemented with 10% fetal bovine serum, 1× nonessential amino acids, 2 mM L-glutamine, 100 U/ml penicillin and 100μg/ml streptomycin and cultured as previously reported (Franco *et al*, 2016).

Viral- and siRNA-mediated genetic manipulations

Viruses and siRNAs used for genetic modifications include mi*MFN2* (Ad-miR2 encoding for 5 miRNAs against MFN2; Sebastián *et al*, 2012), DN-DRP1 (Adenovirus expressing DRP1 K38A; Welgen; gift from the Van Der Bliek lab; Twig *et al*, 2008a), human DRP1 siRNA (Ambion; AM51331; ID#19561; lot#ASO28I4C), mouse Drp1 siRNA (Dharmacon; J-043277-09-0002), and MFN2 OE (Virovek; lot#17-606). Lentiviral vectors for MFN2 knockdown (shMFN2-GFP-pLKO.1), MFN2 OE (MFN2-CFP), and DN-DRP1 (pECFP-C1-DRP1-K38E and Lego iG2 CFP-DRP1-K38E) were a generous gift from Dr. Heidi McBride. Lentiviral particles for genetic manipulation of

 © 2023 The Authors

DLBCLs were generated as described previously (Norberg *et al*, 2017).

HepG2 cells were infected with a MOI of 500 (Ad-miR2 control and Ad-miR2-Mfn2; 5.3×10^{11} particles/ml) or a MOI of 900 (Adenovirus expressing DRP1 K38A; 10^{12} particles/ml) for 24 h in complete culture media. Respirometry, metabolomics, and gene expression were measured on day 3 after transduction (Sebastián *et al*, 2012). Ad-miR2 control, Ad-miR2-Mfn2, and Ad-DN-DRP1 were commercially generated using the service of Welgen (Worcester, MA).

For genetic manipulation of primary hepatocytes, 7×10^5 cells were transfected with 25 nM of Drp1 siRNA using lipofectamine RNAi max (ThermoFisher Scientific, 13778150), or infected with adenoviral particles expressing DN-DRP1 and MFN2 with a MOI of 25 for 24 h, followed by subsequent analysis.

For genetic manipulation of DLBCL cell lines, 5×10^5 cells were infected with lentiviral supernatants for 2 h at 460 *g* as previously described (Caro *et al*, 2012; Norberg *et al*, 2017). The effect of the viral infection on FAO and cell viability was assessed 3 and 5 days after infection, respectively.

Respirometry assays

Respirometry in intact cells

Respirometry of whole murine islets and isolated INS-1 cell mitochondria were performed using the Seahorse Bioscience XF96 platform (Agilent Technologies, Santa Clara, CA) as previously described (Ferrick *et al*, 2008; Wikstrom *et al*, 2012; Taddeo *et al*, 2018). Wild-type islets, β-Mfn2 KO islets, and islets isolated from chow-fed or HFD-fed islets were allowed to recover overnight in culture after isolation prior to respirometry assays. Islets (1–6 per well) were seeded in 1 μl/well Matrigel in a poly-d-lysine–coated XF96 plate and size matched between conditions as previously described (Taddeo *et al*, 2018). INS-1 cells were seeded at 1×10^5 cell per microplate (Seahorse Bioscience) the day prior to experiment as described previously (Wikstrom *et al*, 2012).

Seahorse assays in DLBCL cell lines were performed as previously described (Caro *et al*, 2012).

Respirometry in permeabilized cells

HepG2 and INS-1 cells were permeabilized using 4-8 nM XF PMP reagent (Agilent Technologies, Santa Clara, CA). Respirometry assay was performed in MAS buffer and adapted from a previously reported permeabilization protocol (Divakaruni *et al*, 2014; Yang *et al*, 2021). The following substrates were used: 40 μM palmitoyl-CoA, 0.5 mM carnitine, 40 μM palmitoyl-carnitine, 4 mM ADP, 0.5 μM malonyl-CoA, and 3 μM etomoxir (Sigma-Aldrich, St. Louis, MO). The following compounds were injected: 6 μM oligomycin (Calbiochem, San Diego, CA), 3 μM Carbonyl cyanide 4-(trifluoromethoxy) phenylhydrazone (FCCP; Sigma-Aldrich, St. Louis, MO), 4 μM antimycin A (Sigma-Aldrich, St. Louis, MO). The capacity to oxidize fatty acids was measured as the maximal oxygen consumption fueled by 40 μM palmitoyl-CoA + 5 mM carnitine induced by ADP. All the OCR values were subtracted from the lowest antimycin OCR. Cells were fixed with 4% paraformaldehyde (Thermo Fisher Scientific, Roskilde, Denmark) and normalized by cell count. Cells were stained with 1 μg/ml Hoechst 33342 (Thermo Fisher Scientific, Roskilde, Denmark) then imaged using an Operetta high-throughput imaging device (PerkinElmer, Waltham, MA) with 350/461 nm (EX/EM) under 2× or 10× objectives.

Etomoxir-sensitive respiration was measured by the following equation:

$$\begin{aligned}&\text{Phosphorylating respiration on Palmitoyl}-\text{CoA}\\&\quad=(\text{Palmitoyl}-\text{CoA}+\text{Carnitine}+\text{ADP OCR})\\&\qquad-(\text{Palmitoyl}-\text{CoA}+\text{Carnitine}+\text{Etomoxir}+\text{ADP OCR}).\end{aligned}$$

Maximal respiration stimulated by a given substrate was measured by the following equation as referenced (Yang *et al*, 2021).

$$\begin{aligned}&\text{Maximal OCR on Palmitoyl}-\text{CoA}\\&\quad=\text{MAX OCR (FCCP)}-\text{Antimycin A OCR}.\end{aligned}$$

Confocal microscopy

Confocal microscopy was performed on live cells in glass bottom dishes (MatTek, Ashland, MA) using an inverted Leica TCS SP2 confocal microscope or a Zeiss LSM 710 DUO with a plan apochromat 100× (NA = 1.4) oil immersion objectives. Super-resolution live-cell imaging was performed on a Zeiss LSM880 using a 63× Plan-Apochromat oil-immersion lens and AiryScan super-resolution detector with humidified 5% CO_2 chamber on a temperature-controlled stage (37°C). Mitochondria were stained with 15 nM Tetramethylrhodamine Ethyl Ester Perchlorate (TMRE) (Invitrogen, Eugene, OR.) or with 200 nM MitoTracker green (MTG) (Thermo Fisher Scientific, Roskilde, Denmark) for 45 min prior to imaging. Insulin promoter-mediated paGFP expression in the mitochondrial matrix was induced by lentiviral transduction. The generation of these constructs was previously described in detail (Molina & Shirihai, 2009). MTG was excited with 488 nm laser and TMRE was excited with a 561-nm laser.

Mitochondria of DLBCLs were stained with 100 nM MitoTracker Red CMXRos for 15 min at 37°C. After a PBS wash, cells were seeded on poly-l-lysine–coated coverslips for 30 min at 37°C and fixed with 4% PFA for 30 min at room temperature. Confocal images were collected with the Zeiss LSM 710 Confocal microscope. Images were taken using the 100× oil object and 3.0× zoom. 36 slices at an interval of 0.25 microns were obtained to generate a 3D reconstruction of mitochondrial structures.

Pa-GFP activation and imaging of mitochondria

Photoconversion of PA-GFPmt to its active (fluorescent) form was achieved by using 2-photon laser (750 nm) to give a 375 nm photon-equivalence at the focal plane. This allowed for selective activation of regions that have submicron thickness and are $< 0.5\ \mu m^2$. Using the multitrack scanning mode of the Zeiss LSM-710 microscope and the Zeiss LSM-880, red-emitting TMRE was excited with a 1 mW 543 nm helium/neon laser set at 0.3% and emission was recorded through a BP 650–710 nm filter. Activated PA-GFPmt protein was excited using a 25 mW 488 nm argon laser set at 0.2%. Emission was recorded through a BP 500–550 nm filter. Prior work from our laboratory has determined optimal

© 2023 The Authors

concentrations of probes and laser power to avoid photobleaching in our models (Twig *et al*, 2008b), which was utilized in this study to avoid similar artifacts.

Analysis of mitochondrial morphology

At least 3 separate experiments and 30 cells per condition per experiment were collected for HepG2, INS-1, and islet cells. For DLBCL mitochondrial quantification, between 9 and 13 cells of each lymphoma subtype (Pfeiffer, Toledo, Ly4, K422, DHL-2, DHL-4, DHL-6, Ly1, and U2932) were collected. The cells were individualized as areas of interest using FIJI ImageJ software (https://fiji.sc/). Mitochondria within areas of interest were individualized through the CellProfiler cell image analysis software (https://cellprofiler.org/). To minimize background, images were subjected to a median filter. Segmentation of mitochondria was performed by utilizing a global, three-class, otsu-thresholding method and minimizing the weighted-variance to shape. Identified objects were then subjected to automated mitochondrial form factor (the degree of branching [FF; perimeter2/4π*area]), eccentricity, and aspect ratios (the proportional relationship between width and height) were measured. Data are presented as symbols that represent average individual mitochondrion area, form factor, and aspect ratio per cell. Representative images shown were adjusted in brightness and contrast for better visualization.

Immunostaining and super-resolution microscopy of primary β-cells

For immunostaining, primary islets were plated on poly-D-lysine–coated coverslips 72 h prior to imaging. Cells were fixed at 4% vol/vol paraformaldehyde (PFA) for 15 min at room temperature. After washing two times in PBS, cells were incubated in 50 mM NH_4Cl for 10 min followed by a 10 min 20 mM glycine incubation. Cells were permeabilized (2 μl/ml Triton X-100 and 0.5 mg/ml sodium deoxycholate in PBS, pH 7.4) for 15 min at room temperature. Subsequently, cells were blocked with 10% FBS for 1 h at room temperature. Cells were incubated with 1:200 primary antibody of GPR75/Mortalin (Abcam, ab110325) at 4°C overnight. The next day, cells were washed in PBS and incubated with 1:200 primary antibody of TOM20 (Santa Cruz, Tom20 FL-145) at 4°C for overnight. On the last day, cells were incubated with 1:500 Anti-Rabbit Alexa Fluor 488 (Thermo, A11008) or Anti-Mouse Alexa Fluor 568 antibodies (Thermo, A11004) for 1 h at room temperature, and samples were kept in PBS. A Zeiss LSM 880 confocal microscope in Airyscan mode was used for super-resolution imaging, with a 488 nm Argon laser and Zeiss 63×/1.4NA oil immersion objective.

Representative images

Representative images were generated using FIJI (https://fiji.sc/). Images were cropped for detail, separated into respective channels, and the window and level parameters were adjusted identically per channel in all images to emphasize the fluorescent structures in the images without manipulation of raw pixel values. Images were then inserted in PowerPoint software.

For super-resolution microscopy experiments assessing mitochondrial architecture, the aim was to show morphological changes. In the MTG/TMRE, GPR75/Mortalin, and PAGFPmt experiments photo-correction was used to enhance the brightness of all images by increasing it by 40% and increasing the contrast by 40% for illustrative purposes.

Fatty acid tracing studies for assessment of FAO

HepG2 cells, primary hepatocytes, or DLBCLs subjected to the indicated genetic manipulations or treatment with CPT1 activators (C75 or baicalin) were incubated in culture media supplemented with the fatty acid tracers as detailed below. Treatment with CPT1 activators were as follows: C75 (C5490, Sigma-Aldrich) at 80 μM for 3 h or baicalin (572667, Sigma-Aldrich) at 100 μM for 3 h. Isotope tracers were used at the following concentrations: 200 μM BSA-conjugated $U^{13}C_{16}$-palmitate, 50 μM $^{13}C_1$-hexanoate (all from Cambridge Isotope Laboratories, Tewksbury, MA).

After 2 h incubation with tracers, metabolite extraction was carried out as previously described (Mamer *et al*, 2013; Faubert *et al*, 2014; Fu *et al*, 2020). Briefly, HepG2 and primary hepatocytes were gently washed twice on the plates with 1 ml of ice-cold isotonic saline solution (0.9% NaCl), whereas DLBCLs were pelleted in 1.5 ml Eppendorf tube, followed by two washes of ice-cold isotonic saline solution. Metabolites were then extracted in 350 μl of ice-cold 80% methanol solution supplemented with 2 μg/ml norvaline (as an internal control). Cells were sonicated at high intensity with a 10 s on/off cycle for 10 min at 4°C using a BioRuptor (UCD-200 TM, Diagenode). Samples were then pelleted for 10 min at 16,400 rpm at 4°C. The supernatants were transferred into new vials for drying overnight in a vacuum centrifuge (Labconco, Kansas City, MO) at 4°C. Dried extracts were resuspended in 10 mg/ml methoxylamine in pyridine (Sigma Aldrich). After the incubation for 30 min at 37°C, the samples were derivatized with 70 μl of N-methyl-N-tert-butyldimethylsilyltrifluoroacetamide (MTBSTFA, Sigma) for 1 h at 70°C.

Metabolites were analyzed by GC–MS as previously described (Mamer *et al*, 2013; McGuirk *et al*, 2013; Faubert *et al*, 2014; Fu *et al*, 2020). Briefly, samples were run on an Agilent 5977B mass selective detector coupled to a 7890B gas chromatograph (Agilent Technologies, Santa Clara, CA) with a 7693 autosampler and a DB-5MS + DG capillary column (30 m plus 10 m Duraguard® by Agilent Technologies). Data collection was conducted in electron ionization set at 70 eV. 1 μl of the derivatized sample was injected in split less mode at 280°C (inlet temperature) using helium as a carrier gas with a flow rate of 1.5512 ml/min. The quadrupole was set at 150°C with GC/MS interface at 285°C. The oven program for all the metabolite analyses initiated at 60°C held for 1 min, then increased at a rate of 10°C/min until 320°C. Data were mined in full scan mode (1–600 m/z). All metabolites measured (Table EV2) in this study were previously validated by standards with mass spectra and retention times (Mamer *et al*, 2013). Peak area was integrated using MassHunter Quantitative Analysis (Agilent Technologies). Measurement of carbon flux was conducted using previously developed algorithms including natural isotope enrichment correction and mass isotope distributions (MIDs) (Nanchen *et al*, 2007; McGuirk *et al*, 2013; Fu *et al*, 2020).

Gluconeogenesis assays in primary hepatocytes

Gluconeogenesis assays were performed as previously described (Giménez-Cassina *et al*, 2014). Briefly, primary mouse hepatocytes

© 2023 The Authors

were seeded in six-well plates at 6.7×10^5 and maintained in M199 media supplemented with 1% BSA overnight. The medium was then replaced with 1 ml of glucose-free DMEM without phenol red, supplemented with 20 mM sodium lactate and 2 mM sodium pyruvate. After a 6-h incubation, 200 μl of the medium was collected and pelleted at 3,000 rpm for 2 min to remove floating cells or cell debris, then 30 μl of the supernatants was taken to measure glucose using a colorimetric glucose assay kit (GAGO20-1KT, Sigma-Aldrich). The readings were normalized to total protein concentration as measured by BCA (23227, Thermo Fisher Scientific, Roskilde, Denmark).

Insulin secretion assays in primary islets

Insulin secretion and normalization was performed as previously described (Taddeo *et al*, 2020). Palmitate-induced insulin secretion was performed acutely. Islets were incubated in media containing 0.4 mM palmitate in addition to either 3 mM or 15 mM glucose for 30 min. Insulin was measured using the HTRF Insulin Assay (Cisbio Bioassays, Bedford, MA). Total protein was measured using the Bio-Rad Protein Assay (23225, Thermo Fisher Scientific, Rockford, IL, USA). Insulin content is presented as nanograms of insulin per milligram of total protein. Insulin concentration of the samples was calculated from an insulin standard curve (0–20 or 0–100 ng/ml).

Survival assays

DLBCL cell viability was measured by flow cytometry using Annexin V: FITC Apoptosis Detection Kit (BD Bioscience) as we have previously reported (Caro *et al*, 2012; Norberg *et al*, 2017).

CPT1 activity assays in isolated mitochondria

DLBCL cells or primary hepatocytes were resuspended in mitochondria isolation buffer (MIB; 200 mM mannitol, 70 mM sucrose; 1 mM EGTA; 10 mM HEPES, pH 7.4) supplemented with protease inhibitors, and homogenized with 20 strokes of a teflon-glass homogenizer. The nuclei and cell debris were pelleted at 1,000 *g* for 5 min and the supernatant containing crude mitochondria was washed twice with MIB buffer. The resultant pellet including mitochondria-enriched heavy membrane (HM) fraction was resuspended in MIB for subsequent CPT1 enzyme activity assays.

CPT1 activity was measured using a colorimetric assay based on published protocols with minor modifications (Bieber *et al*, 1972; Shin *et al*, 2005; Kim *et al*, 2012). Briefly, 15 μg of mitochondria-enriched heavy membrane fractions was loaded onto 96-well plates in 200 μl of reaction buffer (2 mM DTNB, 116 mM Tris–HCl [pH 8.0], 2.5 mM EDTA, and 0.2% Triton-X 100). The plates were incubated at room temperature for 20 min to eliminate all pre-existing reactive thiol groups, and the reaction was initiated by adding 100 mM palmitoyl-CoA (Sigma-Aldrich) and 1 mM carnitine (Sigma-Aldrich) as CPT1 substrate and cofactor, respectively. After 20 min incubation, release of CoA-SH from palmitoyl-CoA was spectrophotometrically determined as a readout of CPT1 activity at 412 nm using a Versa Max microplate reader (Molecular Devices, USA) in kinetics mode with 10-s intervals for a total assay time of 1 h. Results were normalized to CPT1 expression levels by immunoblot. To exclude CoA-SH-produced independent of CPT1, CoA-SH was calculated by subtracting CoA produced from D-carnitine in the absence of palmitoyl-CoA (Bieber *et al*, 1972).

Western blot analysis

Cell lysates were diluted in Laemmli sample buffer (100 mM Tris–HCl, 2% SDS, 10% glycerol, 0.1% bromophenol blue) containing 5% β-mercaptoethanol. After heating at 95°C, proteins were separated by SDS–PAGE and transferred onto PVDF membranes. Membranes were blocked with 5% nonfat milk or 5% BSA and detection of individual proteins was carried out by blotting with specific primary antibody against MFN2 (ab56889, AbCam, Cambridge, MA, 1:1,000), CPT1 (GTX114337, GeneTex, 1:1,000), VDAC (Abcam, 1:1,000), Phospho-DRP1 Ser637 (4867S, Cell Signaling, 1:1000), β-actin (NB600-501, Novus Biologicals, Littleton, CO 1:1,000), porin (ab61273, AbCam, Cambridge, MA, 1:1,000), and vinculin (V9131, Sigma-Aldrich, 1:10,000). Proteins of interest were detected by chemiluminescence using a secondary peroxidase-linked anti-rabbit (1:10,000) or anti-mouse (1:10,000) and a detection system.

Histology

Whole pancreata were excised, placed in a tissue cassette, and submerged in 4% paraformaldehyde for at least 24 h. Tissues were embedded in paraffin, sectioned (5 μm), and stained with anti-insulin Alexa Fluor 488 (β-cells), anti-glucagon antibody Alexa Fluor 568 (α-cells), and DAPI ex/em 358/461 (nuclei). Images were taken using an inverted Olympus IX71 microscope equipped with a 40× dry objective and color camera (Photometrics CoolSnap HQ).

Islet perifusion

Islets were perifused in a column as described (Cunningham *et al*, 1996). A collection of 50 islets was laid between 2 layers of Cytodex microcarrier beads (Sigma). The column used was 0.4 cm in diameter and 4 cm high and contained in a temperature-controlled environment at 37°C. Perifusion reagents were pumped through the column at 0.3 ml/min using an analog tubing pump (Ismatech REGLO pump, type ISM 827, model 78016-30; Cole-Parmer Instrument Co., Chicago, IL). Islets were perifused with Krebs–Ringer Bicarbonate buffer with added 0.5% BSA for 30 min to allow for equilibrium. Eluted samples were collected at 30-s intervals for 48 min. Basal levels of insulin were determined with 3 mM glucose perifusion for 6 min, response to 15 mM glucose was measured for 32 min and 15 mM glucose with KCl was perifused for 10 min.

Statistical analysis

Data are expressed as means ± SD or as means ± SEM as indicated in figure legends, and were derived from at least three independent experiments or animals per group. Each independent experiment consisted of at least three technical replicates per condition. P values were calculated using GraphPad Prism software by unpaired two-tailed t test, one-way ANOVA with Sidak or Dunnett multiple comparisons test, two-way ANOVA with Tukey's multiple comparisons test, or linear regression. Statistical significance was set at $P < 0.05$.

© 2023 The Authors

Data availability

No data amenable to database deposition were generated in this study.

Expanded View for this article is available online.

Acknowledgements

We thank Karrie Southwell of the Division of Lab Animal Management at Tufts University for her help and guidance in the breeding of animals and Dr. Hsiuchen Chen at California Institute of Technology for her help with animal genotyping. We thank Dr. Gerald W. Dorn II for providing the MFN2 HR-1 peptide Mfn2-371-384 (378 S(p))-AVRGIMD[S(p)] LHMAAR, Dr. Heidi McBride for MFN2 and DN-DRP1 lentiviral constructs, and Dr. György Hajnóczky for MFN1KO HepG2 cells. We are grateful to Drs. Evan Taddeo, Ilan Y. Benador, Irfan Lodhi, Margaret Shipp, Marc Prentki, and Barbara Corkey for helpful discussion and advice. We also thank Dr. Dani Dagan for critical reading of the manuscript and valuable discussion. This work was supported by the US NIH grants R01DK099618-05 (O.S.S.), R01CA232056-01 (O.S.S.), R21AG060456-01 (O.S.S.), R21AG063373-01 (O.S.S.), R01DK078081 (N.N.D.), R01CA219850 (N.N.D.), R35GM138003 (A.S.D.), P30DK063491 (A.S.D.), F31 CA171400 (I.A.S.); Janssen Research and Development, LLC (ICD #M3229099 – 846328); V Foundation for Cancer Research (N.N.D.), the Claudia Adams Barr Program in Innovative Basic Science Research (N.N.D.), MCINN/AEI/FEDER-PID2021-127278NB-I00 (M.L.), South Korea grants NRF-2022R1C1C1007023 (D.W.C.), and NRF-2022R1A4A3024551 (D.W.C.).

Author contributions

Jennifer Ngo: Conceptualization; data curation; formal analysis; validation; investigation; visualization; methodology; writing – original draft; writing – review and editing. **Dong Wook Choi:** Conceptualization; data curation; formal analysis; funding acquisition; validation; investigation; visualization; methodology; writing – original draft; writing – review and editing. **Illana A Stanley:** Data curation; investigation. **Linsey Stiles:** Data curation; formal analysis; investigation. **Anthony J A Molina:** Data curation; formal analysis; investigation. **Pei-Hsuan Chen:** Data curation; investigation. **Ana Lako:** Investigation. **Isabelle Chiao Han Sung:** Investigation. **Rishov Goswami:** Data curation; investigation. **Min-young Kim:** Investigation. **Nathanael Miller:** Data curation; formal analysis; investigation. **Siyouneh Baghdasarian:** Data curation; formal analysis; investigation. **Doyeon Kim-Vasquez:** Investigation. **Anthony E Jones:** Data curation; investigation; methodology. **Brett Roach:** Validation; investigation. **Vincent Gutierrez:** Investigation. **Karel Erion:** Conceptualization; methodology. **Ajit S Divakaruni:** Resources; funding acquisition; methodology; project administration; writing – review and editing. **Marc Liesa:** Supervision; funding acquisition; investigation; project administration; writing – review and editing. **Nika N Danial:** Conceptualization; resources; supervision; funding acquisition; visualization; writing – original draft; project administration; writing – review and editing. **Orian S Shirihai:** Conceptualization; resources; supervision; funding acquisition; investigation; writing – original draft; project administration; writing – review and editing.

Disclosure and competing interests statement

O.S.S is a co-founder and SAB member of Enspire Bio LLC, Senergy-Bio and Capacity-Bio, and when this study was conducted, he was serving as a consultant to LUCA-Science, IMEL, Epirium, Johnson & Johnson, Pfizer, and Stealth Biotherapeutics. M.L. is a co-founder of Enspire Bio LLC.

References

Aoun M, Feillet-Coudray C, Fouret G, Chabi B, Crouzier D, Ferreri C, Chatgilialoglu C, Wrutniak-Cabello C, Cristol JP, Carbonneau MA *et al* (2012) Rat liver mitochondrial membrane characteristics and mitochondrial functions are more profoundly altered by dietary lipid quantity than by dietary lipid quality: effect of different nutritional lipid patterns. *Br J Nutr* 107: 647–659

Bach D, Naon D, Pich S, Soriano FX, Vega N, Rieusset J, Laville M, Guillet C, Boirie Y, Wallberg-Henriksson H *et al* (2005) Expression of Mfn2, the Charcot-Marie-tooth neuropathy type 2A gene, in human skeletal muscle effects of type 2 diabetes, obesity, weight loss, and the regulatory role of tumor necrosis factor α and Interleukin-6. *Diabetes* 54: 2685–2693

Baron AD, Brechtel G, Edelman SV (1989) Effects of free fatty acids and ketone bodies on *in vivo* non-insulin-mediated glucose utilization and production in humans. *Metabolism* 38: 1056–1061

Benador IY, Veliova M, Mahdaviani K, Petcherski A, Wikstrom JD, Assali EA, Acín-Pérez R, Shum M, Oliveira MF, Cinti S *et al* (2018) Mitochondria bound to lipid droplets have unique bioenergetics, composition, and dynamics that support lipid droplet expansion. *Cell Metab* 27: 869–885.e6

Bieber LL, Abraham T, Helmrath T (1972) A rapid spectrophotometric assay for carnitine palmitoyltransferase. *Anal Biochem* 50: 509–518

Boulton DP, Caino MC (2022) Mitochondrial fission and fusion in tumor progression to metastasis. *Front cell Dev Biol* 10: 849962

Broadfield LA, Pane AA, Talebi A, Swinnen JV, Fendt SM (2021) Lipid metabolism in cancer: new perspectives and emerging mechanisms. *Dev Cell* 56: 1363–1393

Caro P, Kishan AU, Norberg E, Stanley IA, Chapuy B, Ficarro SB, Polak K, Tondera D, Gounarides J, Yin H *et al* (2012) Metabolic signatures uncover distinct targets in molecular subsets of diffuse large B cell lymphoma. *Cancer Cell* 22: 547–560

Casellas-Díaz S, Larramona-Arcas R, Riqué-Pujol G, Tena-Morraja P, Müller-Sánchez C, Segarra-Mondejar M, Gavaldà-Navarro A, Villarroya F, Reina M, Martínez-Estrada OM *et al* (2021) Mfn2 localization in the ER is necessary for its bioenergetic function and neuritic development. *EMBO Rep* 22: e51954

Chauhan SS, Toth RK, Jensen CC, Casillas AL, Kashatus DF, Warfel NA (2020) PIM kinases alter mitochondrial dynamics and chemosensitivity in lung cancer. *Oncogene* 39: 2597–2611

Chen H, Detmer SA, Ewald AJ, Griffin EE, Fraser SE, Chan DC (2003) Mitofusins Mfn1 and Mfn2 coordinately regulate mitochondrial fusion and are essential for embryonic development. *J Cell Biol* 160: 189–200

Chen H, Chomyn A, Chan DC (2005) Disruption of fusion results in mitochondrial heterogeneity and dysfunction. *J Biol Chem* 280: 26185–26192

Chen H, McCaffery JM, Chan DC (2007) Mitochondrial fusion protects against neurodegeneration in the cerebellum. *Cell* 130: 548–562

Chen L, Monti S, Juszczynski P, Ouyang J, Chapuy B, Neuberg D, Doench JG, Bogusz AM, Habermann TM, Dogan A *et al* (2013) SYK inhibition modulates distinct PI3K/AKT- dependent survival pathways and cholesterol biosynthesis in diffuse large B cell lymphomas. *Cancer Cell* 23: 826–838

Cribbs JT, Strack S (2009) Functional characterization of phosphorylation sites in dynamin-related protein 1. *Methods Enzymol* 457: 231–253

Cunningham BA, Deeney JT, Bliss CR, Corkey BE, Tornheim K (1996) Glucose-induced oscillatory insulin secretion in perifused rat pancreatic islets and clonal beta-cells (HIT). *Am J Physiol* 271: E702–E710

Dai J, Liang K, Zhao S, Jia W, Liu Y, Wu H, Lv J, Cao C, Chen T, Zhuang S *et al* (2018) Chemoproteomics reveals baicalin activates hepatic CPT1 to

 © 2023 The Authors

ameliorate diet-induced obesity and hepatic steatosis. *Proc Natl Acad Sci USA* 115: E5896 – E5905

Divakaruni AS, Rogers GW, Murphy AN (2014) Measuring mitochondrial function in permeabilized cells using the seahorse XF analyzer or a Clark-type oxygen electrode. *UNIT Curr Protoc Toxicol* 60: 25.2.1 – 25.2.16

Divakaruni AS, Hsieh WY, Minarrieta L, Duong TN, Kim KKO, Desousa BR, Andreyev AY, Bowman CE, Caradonna K, Dranka BP *et al* (2018) Etomoxir inhibits macrophage polarization by disrupting CoA homeostasis. *Cell Metab* 28: 490 – 503.e7

Eisner V, Picard M, Hajnóczky G (2018) Mitochondrial dynamics in adaptive and maladaptive cellular stress responses. *Nat Cell Biol* 20: 755 – 765

Erion KA, Berdan CA, Burritt NE, Corkey BE, Deeney JT (2015) Chronic exposure to excess nutrients left-shifts the concentration dependence of glucose-stimulated insulin secretion in pancreatic β-cells. *J Biol Chem* 290: 16191 – 16201

Faubert B, Vincent EE, Griss T, Samborska B, Izreig S, Svensson RU, Mamer OA, Avizonis D, Shackelford DB, Shaw RJ *et al* (2014) Loss of the tumor suppressor LKB1 promotes metabolic reprogramming of cancer cells via HIF-1α. *Proc Natl Acad Sci USA* 111: 2554 – 2559

Faubert B, Solmonson A, DeBerardinis RJ (2020) Metabolic reprogramming and cancer progression. *Science* 368: eaaw5473

Faye A, Borthwick K, Esnous C, Price NT, Gobin S, Jackson VN, Zammit VA, Girard J, Prip-Buus C (2005) Demonstration of N- and C-terminal domain intramolecular interactions in rat liver carnitine palmitoyltransferase 1 that determine its degree of malonyl-CoA sensitivity. *Biochem J* 387: 67 – 76

Ferrick DA, Neilson A, Beeson C (2008) Advances in measuring cellular bioenergetics using extracellular flux. *Drug Discov Today* 13: 268 – 274

Fex M, Nitert MD, Wierup N, Sundler F, Ling C, Mulder H (2007) Enhanced mitochondrial metabolism may account for the adaptation to insulin resistance in islets from C57BL/6J mice fed a high-fat diet. *Diabetologia* 50: 74 – 83

Franco A, Kitsis RN, Fleischer JA, Gavathiotis E, Kornfeld OS, Gong G, Biris N, Benz A, Qvit N, Donnelly SK *et al* (2016) Correcting mitochondrial fusion by manipulating mitofusin conformations. *Nature* 540: 74 – 79

Frank S, Gaume B, Bergmann-Leitner ES, Leitner WW, Robert EG, Catez F, Smith CL, Youle RJ (2001) The role of dynamin-related protein 1, a mediator of mitochondrial fission, in apoptosis. *Dev Cell* 1: 515 – 525

Friedman JR, Lackner LL, West M, DiBenedetto JR, Nunnari J, Voeltz GK (2011) ER tubules mark sites of mitochondrial division. *Science* 334: 358 – 362

Frigini EN, Barrera EE, Pantano S, Porasso RD (2020) Role of membrane curvature on the activation/deactivation of carnitine palmitoyltransferase 1A: a coarse grain molecular dynamic study. *Biochim Biophys Acta Biomembr* 1862: 183094

Fu A, Alvarez-Perez JC, Avizonis D, Kin T, Ficarro SB, Choi DW, Karakose E, Badur MG, Evans L, Rosselot C *et al* (2020) Glucose-dependent partitioning of arginine to the urea cycle protects β-cells from inflammation. *Nat Metab* 2: 432 – 446

Galloway CA, Lee H, Brookes PS, Yoon Y (2014) Decreasing mitochondrial fission alleviates hepatic steatosis in a murine model of nonalcoholic fatty liver disease. *Am J Physiol Gastrointest Liver Physiol* 307: G632 – G641

Gan KX, Wang C, Chen JH, Zhu CJ, Song GY (2013) Mitofusin-2 ameliorates high-fat diet-induced insulin resistance in liver of rats. *World J Gastroenterol* 19: 1572 – 1581

Gao AW, Cantó C, Houtkooper RH (2014a) Mitochondrial response to nutrient availability and its role in metabolic disease. *EMBO Mol Med* 6: 580 – 589

Gao Y, Li F, Zhang A, Wang L, Tong W, Liu B (2014b) Evaluation of mitochondrial divisions in mouse with type-2 diabetes and effect of glucose-oxidase on mouse islet cells RIN-m5F. *Cell Biol Int* 38: 368 – 373

García-Ruiz I, Solís-Muñoz P, Fernández-Moreira D, Muñoz-Yagüe T, Solís-Herruzo JA (2015) *In vitro* treatment of HepG2 cells with saturated fatty acids reproduces mitochondrial dysfunction found in nonalcoholic steatohepatitis. *Dis Model Mech* 8: 183 – 191

Gasier HG, Dohl J, Suliman HB, Piantadosi CA, Yu T (2020) Skeletal muscle mitochondrial fragmentation and impaired bioenergetics from nutrient overload are prevented by carbon monoxide. *Am J Physiol Cell Physiol* 319: C746 – C756

Giacomello M, Pyakurel A, Glytsou C, Scorrano L (2020) The cell biology of mitochondrial membrane dynamics. *Nat Rev Mol Cell Biol* 21: 204 – 224

Giménez-Cassina A, Garcia-Haro L, Choi CS, Osundiji MA, Lane EA, Huang H, Yildirim MA, Szlyk B, Fisher JK, Polak K *et al* (2014) Regulation of hepatic energy metabolism and gluconeogenesis by BAD. *Cell Metab* 19: 272 – 284

Gomes LC, Di Benedetto G, Scorrano L (2011) During autophagy mitochondria elongate, are spared from degradation and sustain cell viability. *Nat Cell Biol* 13: 589 – 598

Herńandez-Alvarez MI, Thabit H, Burns N, Shah S, Brema I, Hatunic M, Finucane F, Liesa M, Chiellini C, Naon D *et al* (2010) Subjects with early-onset type 2 diabetes show defective activation of the skeletal muscle PGC-1{alpha}/Mitofusin-2 regulatory pathway in response to physical activity. *Diabetes Care* 33: 645 – 651

Herrera-Cruz MS, Simmen T (2017) Over six decades of discovery and characterization of the architecture at mitochondria-associated membranes (MAMs). *Adv Exp Med Biol* 997: 13 – 31

Itoh K, Murata D, Kato T, Yamada T, Araki Y, Saito A, Adachi Y, Igarashi A, Li S, Pletnikov M *et al* (2019) Brain-specific Drp1 regulates postsynaptic endocytosis and dendrite formation independently of mitochondrial division. *Elife* 8: e44739

Kim MK, Kim SH, Yu HS, Park HG, Kang UG, Ahn YM, Kim YS (2012) The effect of clozapine on the AMPK-ACC-CPT1 pathway in the rat frontal cortex. *Int J Neuropsychopharmacol* 15: 907 – 917

Kraus F, Roy K, Pucadyil TJ, Ryan MT (2021) Function and regulation of the divisome for mitochondrial fission. *Nature* 590: 57 – 66

Kulkarni SS, Joffraud M, Boutant M, Ratajczak J, Gao AW, Maclachlan C, Hernandez-Alvarez MI, Raymond F, Metairon S, Descombes P *et al* (2016) Mfn1 deficiency in the liver protects against diet-induced insulin resistance and enhances the hypoglycemic effect of metformin. *Diabetes* 65: 3552 – 3560

Kuznetsov AV, Usson Y, Leverve X, Margreiter R (2004) Subcellular heterogeneity of mitochondrial function and dysfunction: evidence obtained by confocal imaging. *Mol Cell Biochem* 256–257: 359 – 365

Labbé K, Mookerjee S, Le VM, Gibbs E, Lerner C, Nunnari J (2021) The modified mitochondrial outer membrane carrier MTCH2 links mitochondrial fusion to lipogenesis. *J Cell Biol* 220: e202103122

Lam TKT, Carpentier A, Lewis GF, Van de Werve G, Fantus IG, Giacca A (2003a) Mechanisms of the free fatty acid-induced increase in hepatic glucose production. *Am J Physiol Endocrinol Metab* 284, E863 – 273

Lam TKT, Van de Werve G, Giacca A (2003b) Free fatty acids increase basal hepatic glucose production and induce hepatic insulin resistance at different sites. *Am J Physiol Metab* 284: E281 – E290

Lane EA, Choi DW, Garcia-Haro L, Levine ZG, Tedoldi M, Walker S, Danial NN (2019) HCF-1 regulates *de novo* lipogenesis through a nutrient-sensitive complex with ChREBP. *Mol Cell* 75: 357 – 371.e7

Liang J, Yang Y, Bai L, Li F, Li E (2020) DRP1 upregulation promotes pancreatic cancer growth and metastasis through increased aerobic glycolysis. *J Gastroenterol Hepatol* 35: 885 – 895

Liesa M, Shirihai OS (2013) Mitochondrial dynamics in the regulation of nutrient utilization and energy expenditure. *Cell Metab* 17: 491 – 506

© 2023 The Authors

Liesa M, Palacín M, Zorzano A (2009) Mitochondrial dynamics in mammalian health and disease. *Physiol Rev* 89: 799–845

López-Viñas E, Bentebibel A, Gurunathan C, Morillas M, De Arriaga D, Serra D, Asins G, Hegardt FG, Gómez-Puertas P (2007) Definition by functional and structural analysis of two malonyl-CoA sites in carnitine palmitoyltransferase 1A. *J Biol Chem* 282: 18212–18224

Mahdaviani K, Benador IY, Su S, Gharakhanian RA, Stiles L, Trudeau KM, Cardamone M, Enríquez-Zarralanga V, Ritou E, Aprahamian T *et al* (2017) Mfn2 deletion in brown adipose tissue protects from insulin resistance and impairs thermogenesis. *EMBO Rep* 18: 1123–1138

Malhotra A, Dey A, Prasad N, Kenney AM (2016) Sonic hedgehog signaling drives mitochondrial fragmentation by suppressing Mitofusins in cerebellar granule neuron precursors and medulloblastoma. *Mol Cancer Res* 14: 114–124

Mamer O, Gravel SP, Choinière L, Chénard V, St-Pierre J, Avizonis D (2013) The complete targeted profile of the organic acid intermediates of the citric acid cycle using a single stable isotope dilution analysis, sodium borodeuteride reduction and selected ion monitoring GC/MS. *Metabolomics* 9: 1019–1030

Masini M, Martino L, Marselli L, Bugliani M, Boggi U, Filipponi F, Marchetti P, De Tata V (2017) Ultrastructural alterations of pancreatic beta cells in human diabetes mellitus. *Diabetes Metab Res Rev* 33: e2894

McCune SA, Durant PJ, Jenkins PA, Harris RA (1981) Comparative studies on fatty acid synthesis, glycogen metabolism, and gluconeogenesis by hepatocytes isolated from lean and obese Zucker rats. *Metabolism* 30: 1170–1178

McGarry JD, Brown NF (1997) The mitochondrial carnitine palmitoyltransferase system. From concept to molecular analysis. *Eur J Biochem* 244: 1–14

McGuirk S, Gravel S-P, Deblois G, Papadopoli DJ, Faubert B, Wegner A, Hiller K, Avizonis D, Akavia UD, Jones RG *et al* (2013) PGC-1α supports glutamine metabolism in breast cancer. *Cancer Metab* 1: 22

Merrins MJ, Corkey BE, Kibbey RG, Prentki M (2022) Metabolic cycles and signals for insulin secretion. *Cell Metab* 34: 947–968

Molina AJA, Shirihai OS (2009) Monitoring mitochondrial dynamics with photoactivatable [corrected] green fluorescent protein. *Methods Enzymol* 457: 289–304

Molina AJA, Wikstrom JD, Stiles L, Las G, Mohamed H, Elorza A, Walzer G, Twig G, Katz S, Corkey BE *et al* (2009) Mitochondrial networking protects β-cells from nutrient-induced apoptosis. *Diabetes* 58: 2303–2315

Nagdas S, Kashatus JA, Nascimento A, Hussain SS, Trainor RE, Pollock SR, Adair SJ, Michaels AD, Sesaki H, Stelow EB *et al* (2019) Drp1 promotes KRas-driven metabolic changes to drive pancreatic tumor growth. *Cell Rep* 28: 1845–1859.e5

Nanchen A, Fuhrer T, Sauer U (2007) Determination of metabolic flux ratios from 13C-experiments and gas chromatography-mass spectrometry data: protocol and principles. *Methods Mol Biol* 358: 177–197

Ngo J, Osto C, Villalobos F, Shirihai OS (2021) Mitochondrial heterogeneity in metabolic diseases. *Biology (Basel)* 10: 927

Nicot C, Napal L, Relat J, González S, Llebaria A, Woldegiorgis G, Marrero PF, Haro D (2004) C75 activates malonyl-CoA sensitive and insensitive components of the CPT system. *Biochem Biophys Res Commun* 325: 660–664

Nolan CJ, Leahy JL, Delghingaro-Augusto V, Moibi J, Soni K, Peyot M-LL, Fortier M, Guay C, Lamontagne J, Barbeau A *et al* (2006a) Beta cell compensation for insulin resistance in Zucker fatty rats: increased lipolysis and fatty acid signalling. *Diabetologia* 49: 2120–2130

Nolan CJ, Madiraju MSR, Delghingaro-Augusto V, Peyot M-L, Prentki M (2006b) Fatty acid signaling in the beta-cell and insulin secretion. *Diabetes* 55: S16–S23

Norberg E, Lako A, Chen PH, Stanley IA, Zhou F, Ficarro SB, Chapuy B, Chen L, Rodig S, Shin D *et al* (2017) Differential contribution of the mitochondrial translation pathway to the survival of diffuse large B-cell lymphoma subsets. *Cell Death Differ* 24: 251–262

Rao JN, Warren GZL, Estolt-Povedano S, Zammit VA, Ulmer TS (2011) An environment-dependent structural switch underlies the regulation of carnitine palmitoyltransferase 1A. *J Biol Chem* 286: 42545–42554

Raud B, Roy DG, Divakaruni AS, Tarasenko TN, Franke R, Ma EH, Samborska B, Hsieh WY, Wong AH, Stüve P *et al* (2018) Etomoxir actions on regulatory and memory T cells are independent of Cpt1a-mediated fatty acid oxidation. *Cell Metab* 28: 504–515.e7

Reddy JK, Hashimoto T (2001) Peroxisomal β-oxidation and peroxisome proliferator-activated receptor α: an adaptive metabolic system. *Annu Rev Nutr* 21: 193–230

Rocha AG, Franco A, Krezel AM, Rumsey JM, Alberti JM, Knight WC, Biris N, Zacharioudakis E, Janetka JW, Baloh RH *et al* (2018) MFN2 agonists reverse mitochondrial defects in preclinical models of Charcot-Marie-tooth disease type 2A. *Science* 360: 336–341

Rui L (2014) Energy metabolism in the liver. *Compr Physiol* 4: 177–197

Satapati S, Kucejova B, Duarte JAG, Fletcher JA, Reynolds L, Sunny NE, He T, Arya Nair L, Livingston K, Fu X *et al* (2015) Mitochondrial metabolism mediates oxidative stress and inflammation in fatty liver. *J Clin Invest* 125: 4447–4462

Sebastián D, Hernández-Alvarez MI, Segalés J, Sorianello E, Muñoz JP, Sala D, Waget A, Liesa M, Paz JC, Gopalacharyulu P *et al* (2012) Mitofusin 2 (Mfn2) links mitochondrial and endoplasmic reticulum function with insulin signaling and is essential for normal glucose homeostasis. *Proc Natl Acad Sci USA* 109: 5523–5528

Sebastián D, Palacín M, Zorzano A (2017) Mitochondrial dynamics: coupling mitochondrial fitness with healthy aging. *Trends Mol Med* 23: 201–215

Serasinghe MN, Wieder SY, Renault TT, Elkholi R, Asciolla JJ, Yao JL, Jabado O, Hoehn K, Kageyama Y, Sesaki H *et al* (2015) Mitochondrial division is requisite to RAS-induced transformation and targeted by oncogenic MAPK pathway inhibitors. *Mol Cell* 57: 521–536

Shenouda SM, Widlansky ME, Chen K, Xu G, Holbrook M, Tabit CE, Hamburg NM, Frame AA, Caiano TL, Kluge MA *et al* (2011) Altered mitochondrial dynamics contributes to endothelial dysfunction in diabetes mellitus. *Circulation* 124: 444–453

Shin ES, Cho SY, Lee EH, Lee SJ, Chang IS, Lee TR (2005) Positive regulation of hepatic carnitine palmitoyl transferase 1A (CPT1A) activities by soy isoflavones and L–carnitine. *Eur J Nutr* 453: 159–164

Sisalli MJ, Ianniello G, Savoia C, Cuomo O, Annunziato L, Scorziello A (2020) Knocking-out the Siah2 E3 ubiquitin ligase prevents mitochondrial NCX3 degradation, regulates mitochondrial fission and fusion, and restores mitochondrial function in hypoxic neurons. *Cell Commun Signal* 18: 42

Song JE, Alves TC, Stutz B, Šestan-Peša M, Kilian N, Jin S, Diano S, Kibbey RG, Horvath TL (2021) Mitochondrial fission governed by Drp1 regulates exogenous fatty acid usage and storage in Hela cells. *Metabolites* 11: 322

Sprenger HG, Langer T (2019) The good and the bad of mitochondrial breakups. *Trends Cell Biol* 29: 888–900

Steffen J, Ngo J, Wang SP, Williams K, Kramer HF, Ho G, Rodriguez C, Yekkala K, Amuzie C, Bialecki R *et al* (2022) The mitochondrial fission protein Drp1 in liver is required to mitigate NASH and prevents the activation of the mitochondrial ISR. *Mol Metab* 64: 101566

Stiles L, Shirihai OS (2012) Mitochondrial dynamics and morphology in beta-cells. *Best Pract Res Clin Endocrinol Metab* 26: 725–738

Supale S, Li N, Brun T, Maechler P (2012) Mitochondrial dysfunction in pancreatic β cells. *Trends Endocrinol Metab* 23: 477–487

 © 2023 The Authors

Taddeo EP, Stiles L, Sereda S, Ritou E, Wolf DM, Abdullah M, Swanson Z, Wilhelm J, Bellin M, McDonald P *et al* (2018) Individual islet respirometry reveals functional diversity within the islet population of mice and human donors. *Mol Metab* 16: 150–159

Taddeo EP, Alsabeeh N, Baghdasarian S, Wikstrom JD, Ritou E, Sereda S, Erion K, Li J, Stiles L, Abdulla M *et al* (2020) Mitochondrial proton leak regulated by cyclophilin D elevates insulin secretion in islets at nonstimulatory glucose levels. *Diabetes* 69: 131–145

Thupari JN, Landree LE, Ronnett GV, Kuhajda FP (2002) C75 increases peripheral energy utilization and fatty acid oxidation in diet-induced obesity. *Proc Natl Acad Sci USA* 99: 9498–9502

Tondera D, Grandemange S, Jourdain A, Karbowski M, Mattenberger Y, Herzig S, Da Cruz S, Clerc P, Raschke I, Merkwirth C *et al* (2009) SLP-2 is required for stress-induced mitochondrial hyperfusion. *EMBO J* 28: 1589–1600

Trotta AP, Chipuk JE (2017) Mitochondrial dynamics as regulators of cancer biology. *Cell Mol Life Sci* 74: 1999–2017

Twig G, Elorza A, Molina AJA, Mohamed H, Wikstrom JD, Walzer G, Stiles L, Haigh SE, Katz S, Las G *et al* (2008a) Fission and selective fusion govern mitochondrial segregation and elimination by autophagy. *EMBO J* 27: 433–446

Twig G, Hyde B, Shirihai OS (2008b) Mitochondrial fusion, fission and autophagy as a quality control axis: the bioenergetic view. *Biochim Biophys Acta Bioenerg* 1777: 1092–1097

Ugarte-Uribe B, Prévost C, Das KK, Bassereau P, Garciá-Sáez AJ (2018) Drp1 polymerization stabilizes curved tubular membranes similar to those of constricted mitochondria. *J Cell Sci* 132: jcs208603

Valera-Alberni M, Joffraud M, Miro-Blanch J, Capellades J, Junza A, Dayon L, Núñez Galindo A, Sanchez-Garcia JL, Valsesia A, Cercillieux A *et al* (2021) Crosstalk between Drp1 phosphorylation sites during mitochondrial remodeling and their impact on metabolic adaptation. *Cell Rep* 36: 109565

Vander Heiden MG, DeBerardinis RJ (2017) Understanding the intersections between metabolism and cancer biology. *Cell* 168: 657–669

Violante S, Achetib N, van Roermund CWT, Hagen J, Dodatko T, Vaz FM, Waterham HR, Chen H, Baes M, Yu C *et al* (2019) Peroxisomes can oxidize medium- and long-chain fatty acids through a pathway involving ABCD3 and HSD17B4. *FASEB J* 33: 4355–4364

Wang L, Ishihara T, Ibayashi Y, Tatsushima K, Setoyama D, Hanada Y, Takeichi Y, Sakamoto S, Yokota S, Mihara K *et al* (2015) Disruption of mitochondrial fission in the liver protects mice from diet-induced obesity and metabolic deterioration. *Diabetologia* 58: 2371–2380

Wikstrom JD, Sereda SB, Stiles L, Elorza A, Allister EM, Neilson A, Ferrick DA, Wheeler MB, Shirihai OS (2012) A novel high-throughput assay for islet respiration reveals uncoupling of rodent and human islets. *PLoS One* 7: e33023

Xu X, Krumm C, So JS, Bare CJ, Holman C, Gromada J, Cohen DE, Lee AH (2018) Preemptive activation of the integrated stress response protects mice from diet-induced obesity and insulin resistance by fibroblast growth factor 21 induction. *Hepatology* 68: 2167–2181

Yang K, Doan MT, Stiles L, Divakaruni AS (2021) Measuring CPT-1-mediated respiration in permeabilized cells and isolated mitochondria. *STAR Protoc* 2: 100687

Zammit VA, Corstorphine CG, Kolodziej MP, Fraser F (1998) Lipid molecular order in liver mitochondrial outer membranes, and sensitivity of carnitine palmitoyltransferase I to malonyl-CoA. *Lipids* 33: 371–376

License: This is an open access article under the terms of the Creative Commons Attribution License, which permits use, distribution and reproduction in any medium, provided the original work is properly cited.

Expanded View Figures

Figure EV1. Genetic perturbation of mitochondrial morphology has selective effects on mitochondrial substrate oxidation.

A Quantification of phosphorylating ADP and maximal respiration induced by palmitoyl-CoA in permeabilized HepG2 cells. $n = 5$ independent biological replicates for siCtrl and $n = 3$ independent biological replicates with six technical repeats for all other perturbations. Data represent means ± SEM, *t*-test. *$P < 0.05$.

B Succinate-induced respiration in permeabilized HepG2 cells. $n = 3$ biological replicates with six technical repeats. Data represent means ± SEM, *t*-test. *$P < 0.05$.

C Representative images of MFN1 KO HepG2 cells and quantification of mitochondrial length. Scale bar: 10 μm. Data represent means ± SEM, $n = 3$, *t*-test. *$P < 0.05$.

D–F Quantification of CPT1 protein expression in HepG2 cells following expression of DN-DRP1 (D), excess nutrient treatment (E), or mi*MFN2* (F).

 © 2023 The Authors

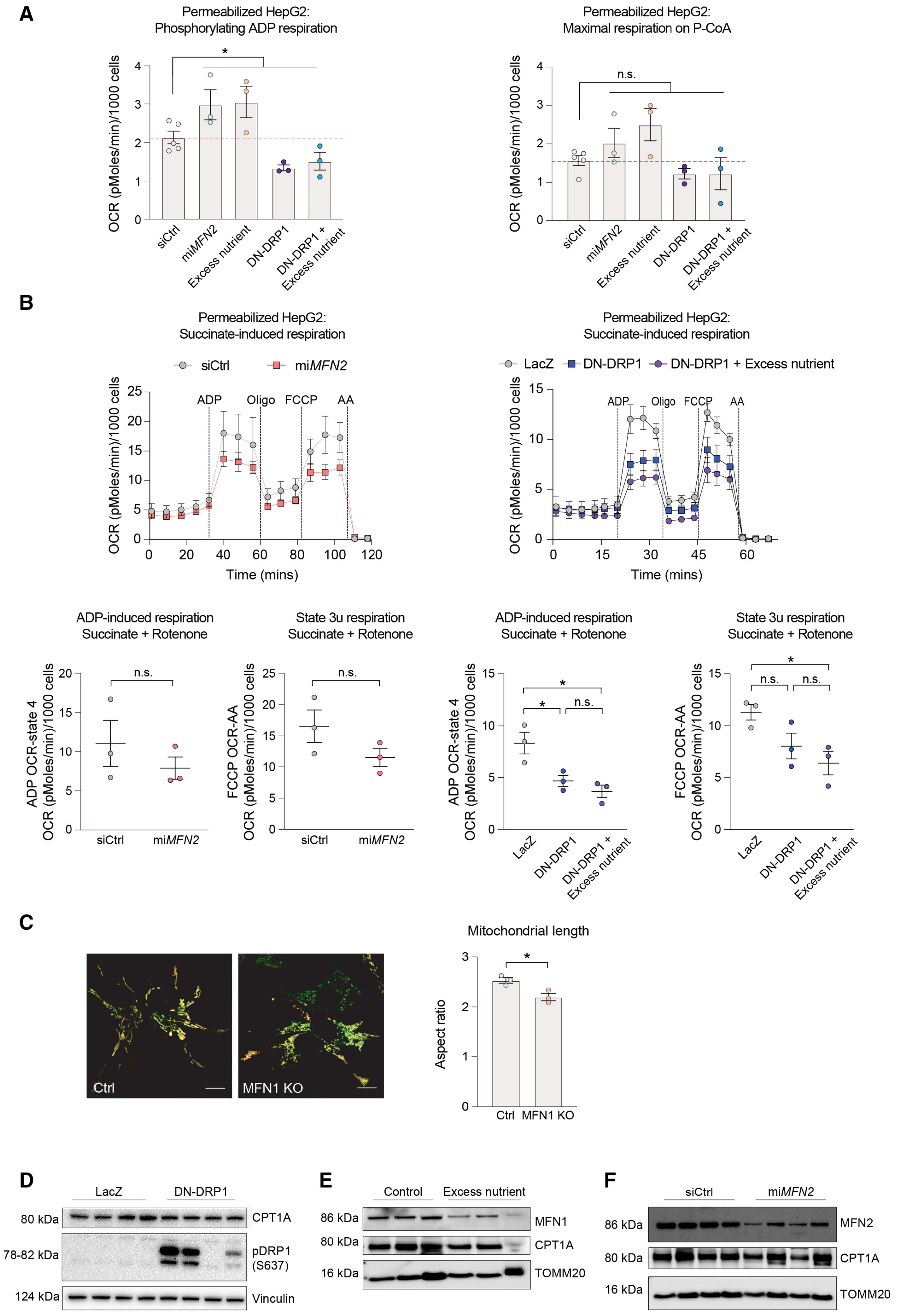
A
Permeabilized HepG2: Phosphorylating ADP respiration
OCR (pMoles/min)/1000 cells
siCtrl
miMFN2
Excess nutrient
DN-DRP1
DN-DRP1 + Excess nutrient
*
Permeabilized HepG2: Maximal respiration on P-CoA
n.s.
B
Permeabilized HepG2: Succinate-induced respiration
siCtrl
miMFN2
ADP
Oligo
FCCP
AA
Time (mins)
LacZ
DN-DRP1
DN-DRP1 + Excess nutrient
ADP-induced respiration Succinate + Rotenone
ADP OCR-state 4 OCR (pMoles/min)/1000 cells
State 3u respiration Succinate + Rotenone
FCCP OCR-AA OCR (pMoles/min)/1000 cells
n.s.
*
C
Ctrl
MFN1 KO
Mitochondrial length
Aspect ratio
D
LacZ
DN-DRP1
80 kDa CPT1A
78-82 kDa pDRP1 (S637)
124 kDa Vinculin
E
Control
Excess nutrient
86 kDa MFN1
80 kDa CPT1A
16 kDa TOMM20
F
siCtrl
miMFN2
86 kDa MFN2
80 kDa CPT1A
16 kDa TOMM20

Figure EV1.

Figure EV2. Characterization of MFN2 expression in islets from obese rodent models and generation of β-Mfn2KO mice.

A Representative western blot showing MFN2 protein levels in islets derived from $n = 4$ male C57BL/6J mice fed on CHOW or HFD for 12 weeks. Porin serves as loading control. Immunoblots were quantified by ImageJ. Data represent means ± SEM *t*-test. *$P < 0.05$.

B MFN2 protein levels in islets derived from 8-week-old Zucker Lean (ZL), Zucker Fatty (ZF), Zucker Diabetic Lean (ZDL), and Zucker Diabetic Fatty (ZDF) rats. The ZF rat is a model of human obesity displaying phenotypes of hyperlipidemia and hypertension. The ZDF rat models diabetes, displaying hyperglycemia, hyperlipidemia, and hypertension although the ZDF rat strain are less obese than the ZF, they are more insulin resistant. The ZDL rat model is hyperglycemic but not hyperlipidemic. Islet protein lysates were analyzed from $n = 3$ rats per group and immunoblots were quantified using ImageJ. Data represent means ± SEM *t*-test. **$P < 0.01$. n.s, nonsignificant.

C Validation of *Mfn2* deletion in β-Mfn2KO islets by western blot analysis compared to control LoxP islets with β-actin serving as loading control. Any residual MFN2 protein in islet lysates is attributed to non-β-cell types in β-Mfn2KO islets.

D Representative images of immunohistochemical analyses of pancreatic sections from LoxP control and β-Mfn2KO mice, where β- and α-cells are stained with anti-insulin (green) and anti-glucagon (red) antibodies, respectively. Nuclei are stained with DAPI (blue). A total of 45 sections (LoxP control $n = 21$ and β-Mfn2KO mice $n = 24$) were similarly analyzed and images were subsequently quantified for ratios of β- to α-cells in the two genotypes, *t*-test. n.s, nonsignificant. Scale bar: 75 μm.

E Insulin content in LoxP control and β-Mfn2KO islets derived from $n = 4$ mice per group; technical replicates of six islets from each animal are shown. Data represent means ± SEM, *t*-test. n.s, nonsignificant.

F Quantification of dynamic insulin secretion in islet perifusion assays using LoxP control and β-Mfn2KO islets derived from $n = 4$ mice per group. β-Mfn2KO islets show a slow rising first phase secretion that remains significantly higher during the exposure to high glucose, whereas the normal response of an islet at high glucose is a sharp peak that eventually decreases to basal levels. Subsequent exposure to 40 mM KCl caused a similar excursion in insulin secretion in both genetic groups, although β-Mfn2KO islets continued to secrete at a higher rate, overall confirming the changes in insulin secretory behavior seen in static incubation assays in Fig 2G. Data represent means ± SD, AUC denotes area under the curve.

 © 2023 The Authors

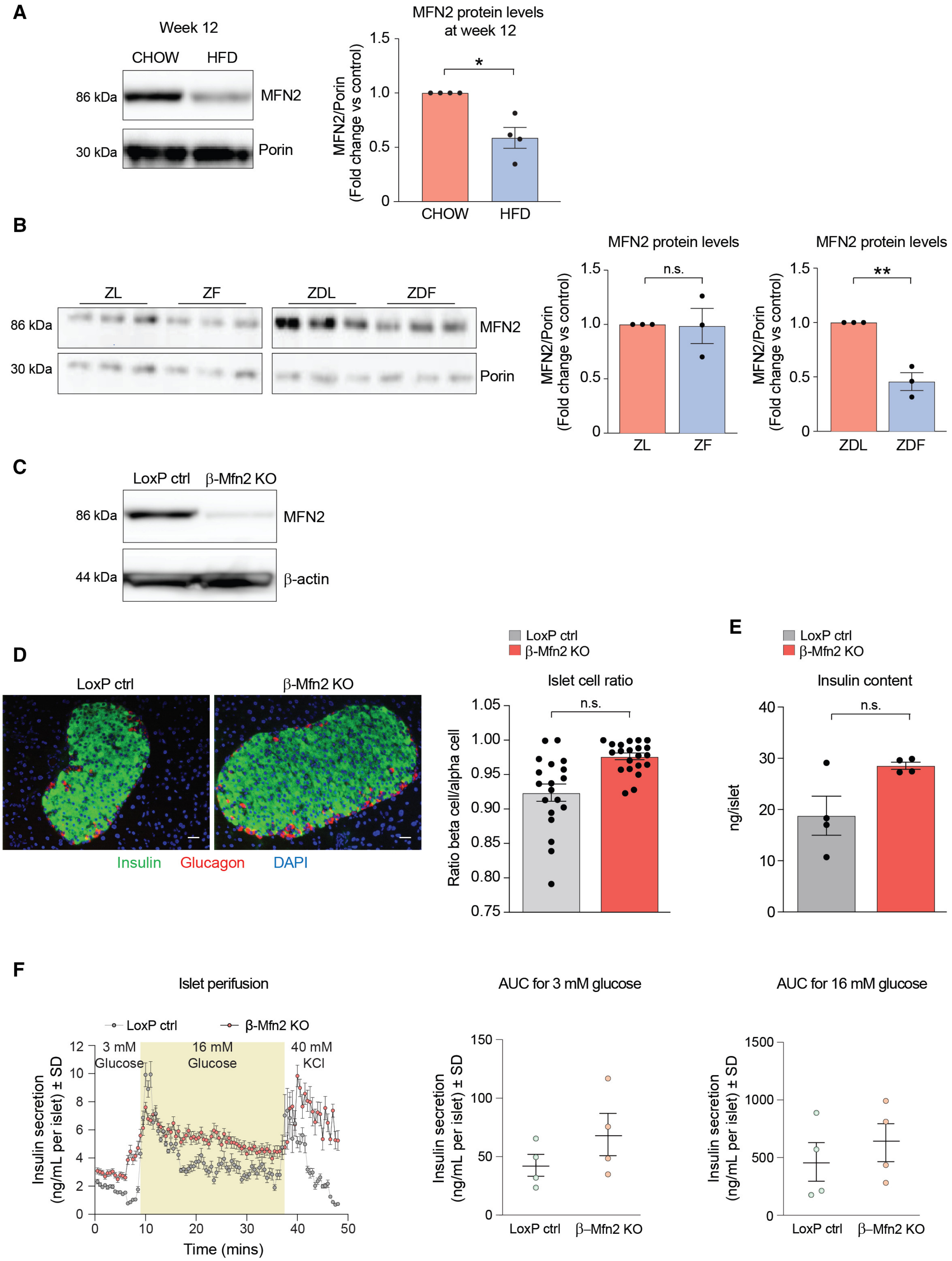
A
Week 12
CHOW HFD
86 kDa MFN2
30 kDa Porin
MFN2 protein levels at week 12
MFN2/Porin (Fold change vs control)
CHOW HFD
B
ZL ZF ZDL ZDF
86 kDa MFN2
30 kDa Porin
MFN2 protein levels
n.s.
ZL ZF
MFN2 protein levels
**
ZDL ZDF
C
LoxP ctrl β-Mfn2 KO
86 kDa MFN2
44 kDa β-actin
D
LoxP ctrl β-Mfn2 KO
Insulin Glucagon DAPI
LoxP ctrl
β-Mfn2 KO
Islet cell ratio
Ratio beta cell/alpha cell
E
LoxP ctrl
β-Mfn2 KO
Insulin content
ng/islet
F
Islet perifusion
LoxP ctrl β-Mfn2 KO
3 mM Glucose
16 mM Glucose
40 mM KCl
Insulin secretion (ng/mL per islet) ± SD
Time (mins)
AUC for 3 mM glucose
AUC for 16 mM glucose
LoxP ctrl β–Mfn2 KO

Figure EV2.

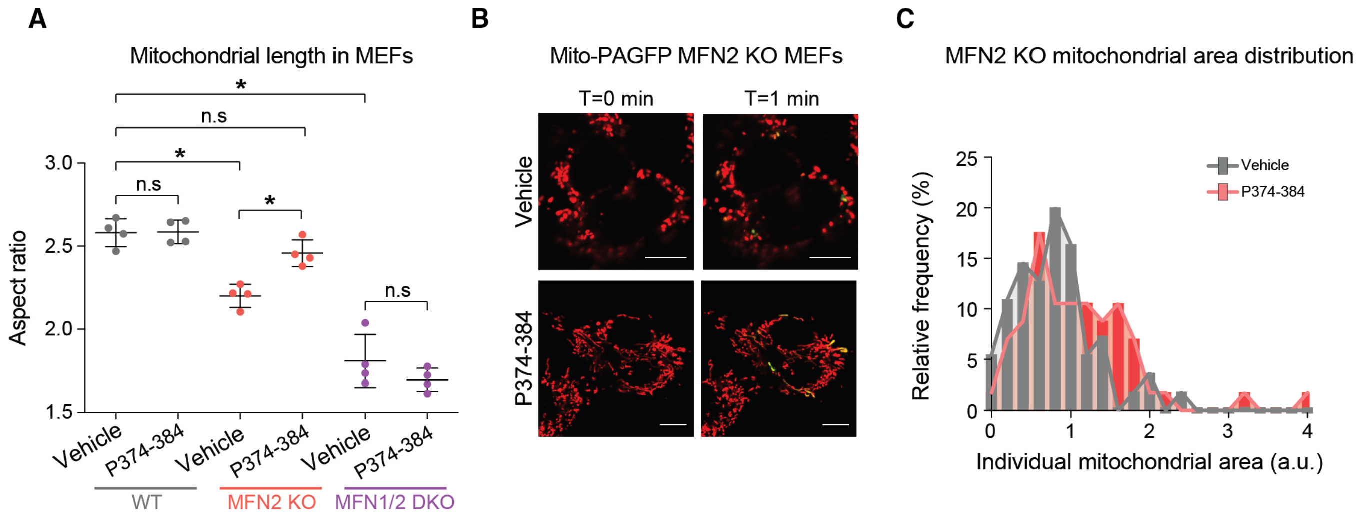

Figure EV3. Validation of the MFN2 agonist peptide MFN2-TAT-P374-384.

A Quantification of mitochondrial length in response to P374-384 treatment in mouse embryonic fibroblasts (MEFs) from the indicated genotypes. Data represent means ± SEM, $n = 4$ independent experiments with 30–50 cells analyzed per experiment, *t*-test. $^{*}P < 0.05$, n.s, nonsignificant.

B Representative confocal images of mitochondria in MFN2 KO MEFs treated with vehicle or P374-384 and labeled with TMRE (red) and mito-PAGFP (green). Enhanced fusion was identified by 2-photon-mediated photoconversion of PAGFP. Scale bar: 10 μm.

C Quantification of mitochondrial area in MFN2 KO MEFs treated with vehicle (gray) or P374-384 (red). Data are from $n = 4$ independent experiments with 30–50 cells analyzed per experiment.

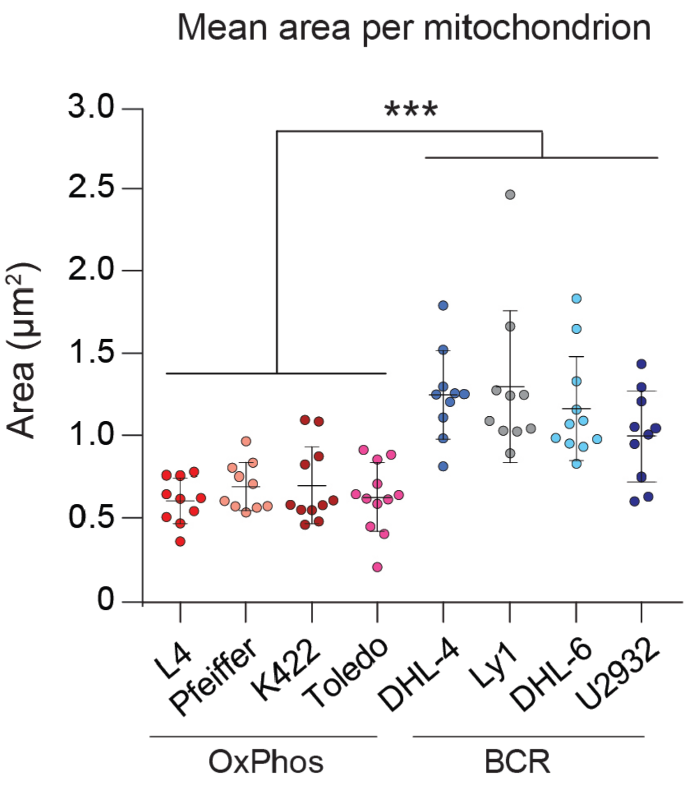

Figure EV4. Net increase in mitochondrial fragmentation in FAO prone OxPhos-DLBCLs compared with BCR-DLBCLs.

Average area of individual mitochondria in an expanded set of OxPhos- (red) and BCR-DLBCL (blue) cell lines labeled as in Fig 3A. Data represent means ± SEM with $n = 10$ cells analyzed per cell line, unpaired *t*-test with Welch's correction. $^{***}P < 0.001$.

 © 2023 The Authors

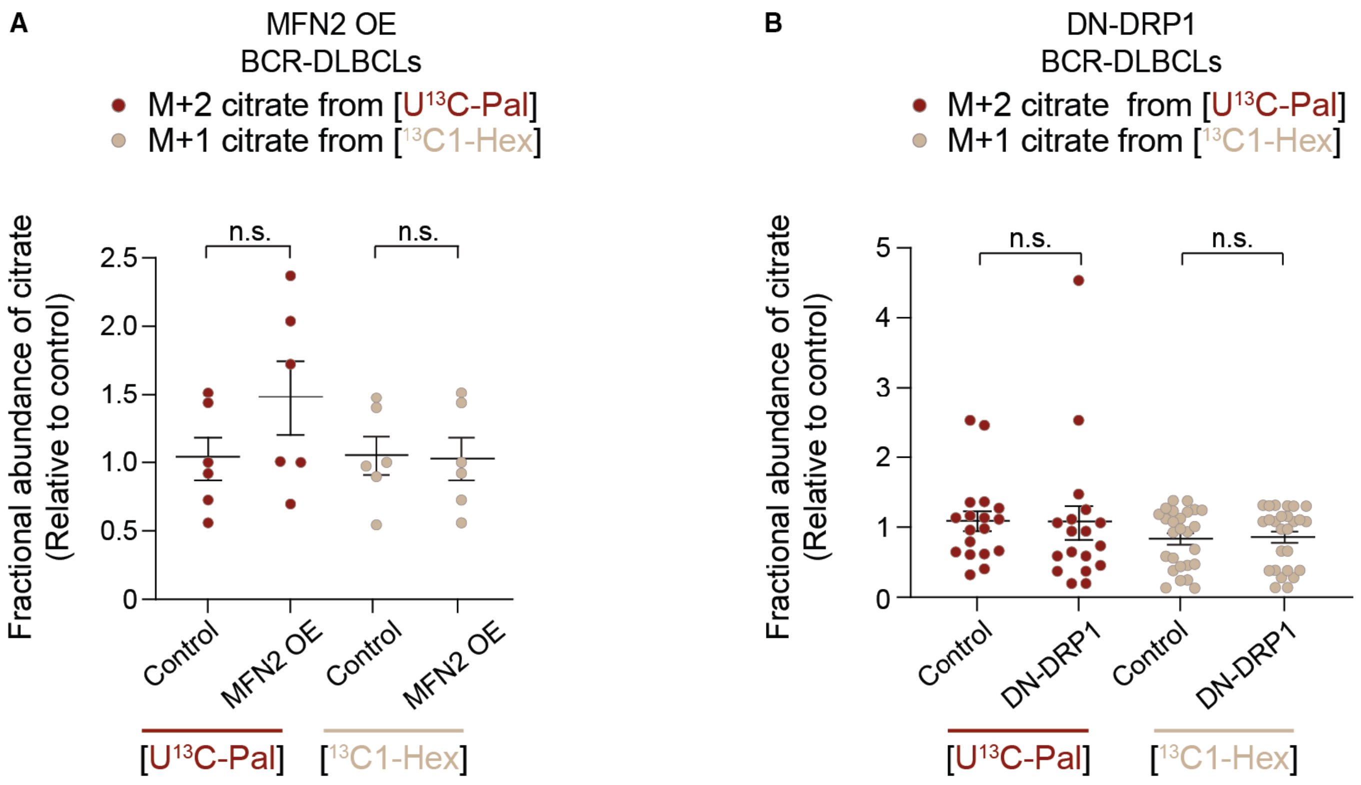

Figure EV5. Forced mitochondrial elongation in BCR-DLBCLs does not alter FAO.

A, B Fractional abundance of m + 2 citrate from [U-$^{13}C_{16}$] palmitate (LCFAO) compared with m + 1 citrate from [$^{13}C_1$] hexanoate (SCFAO) following forced mitochondrial elongation by MFN OE (A) or DN-DRP1 (B) in BCR-DLBCLs. Data represent means ± SEM and are cumulative data points from multiple individual experiments using independent cell lines as follows: n = 3–4 individual experiments in (A) using two independent BCR-DLBCL cell lines, n = 5 individual experiments in (B) using four independent BCR-DLBCL cell lines, t-test. n.s, nonsignificant. The [U-$^{13}C_{16}$] palmitate arm of these experiments is the same as shown in Fig 3F and G.

© 2023 The Authors

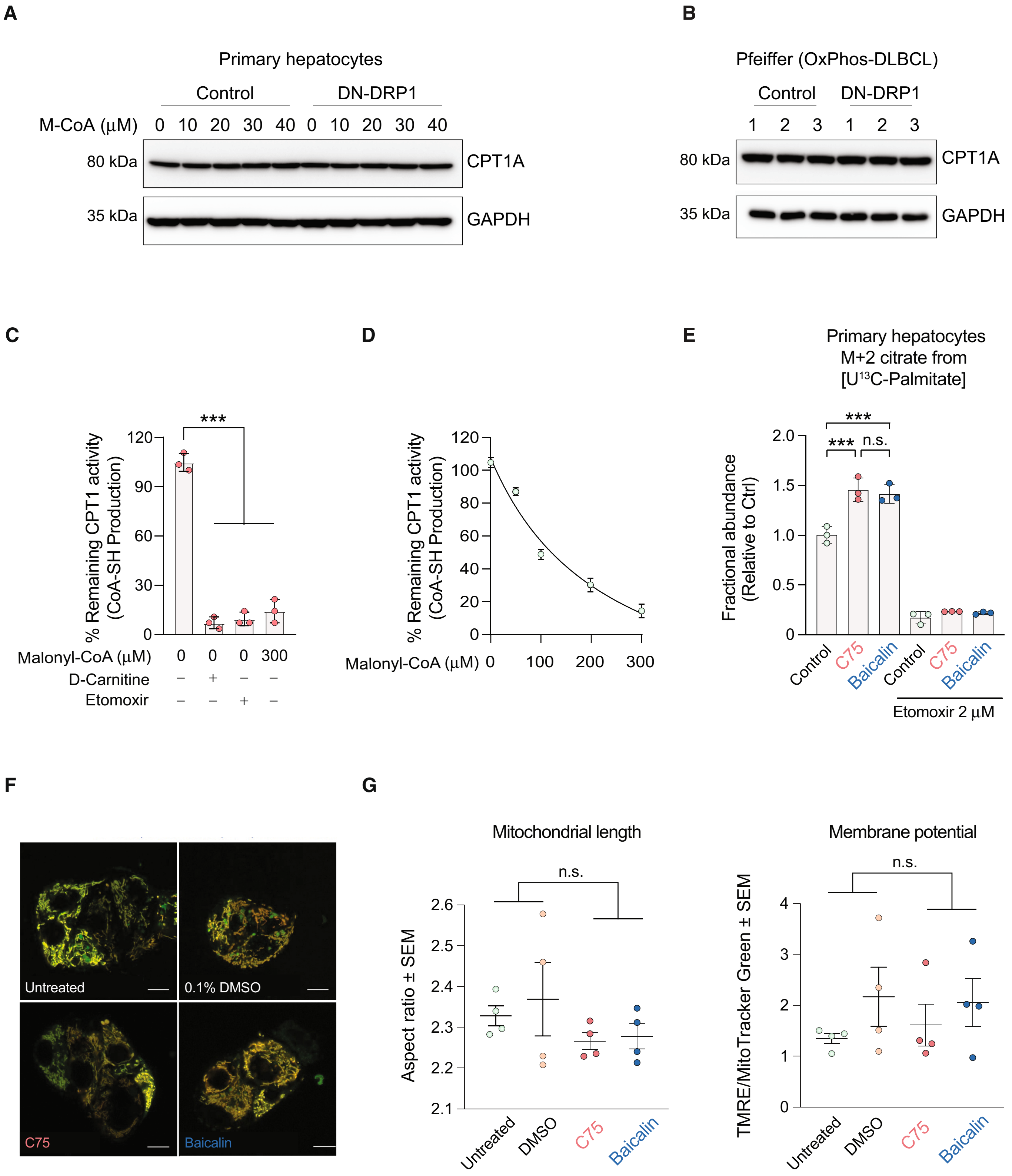

© 2023 The Authors

Figure EV6. Validation of CPT1 activity and expression.

A, B Quantification of CPT1 protein expression in primary hepatocytes (A) and the OxPhos-DLBCL cell line Pfeiffer (B) following expression of DN-DRP1 and varying concentrations of malonyl-CoA.

C CPT1 activity in the absence or presence of 2 μM etomoxir, 0 μM palmitoyl-CoA, and 0 and 300 μM M-CoA measured in mitochondria-enriched heavy membrane fractions isolated from control primary hepatocytes. Enzyme activity was normalized to CPT1 protein levels in individual experiments. Data represent means ± SD, $n = 3$ individual experiments from primary hepatocytes, *t*-test. ***$P < 0.001$.

D Full titration curve of CPT1 activity in the presence of increasing malonyl-CoA (M-CoA) concentrations measured in mitochondria-enriched heavy membrane fractions isolated from control primary mouse hepatocytes. Enzyme activity was normalized to CPT1 protein levels in individual experiments. Data represent means ± SD, $n = 3$.

E Fractional abundance of m + 2 citrate from [U-$^{13}C_{16}$] palmitate in primary hepatocytes treated with C75 and baicalin in the absence or presence of 2 μM etomoxir. Data represent means ± SD, $n = 3$ individual experiments from primary hepatocytes, *t*-test. ***$P < 0.001$.

F, G Representative confocal images (F) and quantification (G) of mitochondrial morphology and membrane potential of mitochondria in HepG2 cells treated with vehicle, C75, or baicalin and labeled with TMRE (red) and MitoTracker Green (green). Data represent means ± SEM, $n = 4$ independent biological replicates, 20 cells imaged per independent experiment, *t*-test. Scale bar: 10 μm.

© 2023 The Authors
