# Supplementary material for: Mitochondrial morphology controls fatty acid utilization by changing CPT1 sensitivity to malonyl‐CoA
Source: EMBO J. 2023 Mar 14;42(11):e111901. doi: 10.15252/embj.2022111901 (PMC10233380; doi:10.15252/embj.2022111901)
Supplement: Supplementary file 3 — Table EV2 [file EMBJ-42-e111901-s003.pdf]

Figure 1E

Samples	siCtrl			mi*MFN2*		
Citrate M+2 (%)	16.7353	4.72748	21.1455	21.8973	5.75999	24.5577

Figure 1F

Samples	Ctrl			MFN1 KO			Ctrl+Eto			MFN1 KO+Eto		
Citrate M+2 (%)	16.9913	16.595	16.3045	18.1227	20.7772	21.0787	3.2363	2.5327	1.8654	1.9463	2.797	3.0757

Figure 1G

Samples	Ctrl			MFN1 OE		
Citrate M+2 (%)	38.4347	39.0319	37.54	29.3822	27.2629	27.0902

Figure 1H

Samples	Ctrl			si*DRP1*		
Citrate M+2 (%)	43.603	44.275	45.65	39.9	39.729	38.093

Figure 1I

Samples	siCtrl			DN-DRP1		
Citrate M+2 (%)	31.288	31.328	31.674	26.246	26.554	24.592

Figure 3F

U13C-Pal	OxPhos-DLBCL											BCR-DLBCL												
Samples	Peiffer			Peiffer + MFN2			Toledo			Toledo + MFN2		DHL4			DHL4 + MFN2			DHL6			DHL6 + MFN2			
Citrate M+2 (%)	15.732	14.614	14.375	12.928	12.526	12.418	3.114	2.8498	3.4552	1.7113	1.5117	2.8525	2.0703	1.5948	2.6243	4.3198	4.1	0.4657	0.9498	1.1038	0.4695	0.8026	0.3249	

1-13C Hex	OxPhos-DLBCL											BCR-DLBCL												
Samples	Peiffer			Peiffer + MFN2			Toledo			Toledo + MFN2			DHL4			DHL4 + MFN2			DHL6			DHL6 + MFN2		
Citrate M+1 (%)	7.1892	6.6779	6.5684	7.0277	6.5217	6.4245	7.0303	6.5793	7.9769	7.0304	6.4339	7.8007	5.0327	4.9047	2.7422	4.5124	7.4276	7.0487	3.4921	2.5342	1.9524	3.2171	5.2884	5.0193

Figure 4D

Samples	Control			DN-DRP1			Control			DN-DRP1		
Citrate M+2 (%)	31.288	31.328	31.674	26.246	26.554	24.592	1.3251	1.5824	1.1691	1.2443	1.4679	1.5037
Citrate M+1 (%)	4.3838	4.2946	4.4784	4.5035	4.7682	6.7234	23.261	24.326	23.762	22.905	23.552	24.458

Figure 4E

	U13C-Pal						1-13C Hex					
Samples	siCtrl			si*DRP1*			siCtrl			si*DRP1*		
Citrate M+2 (%)	42.891	41.945	42.341	37.598	38.933	37.693	43.603	44.275	45.65	39.9	39.729	38.093
Citrate M+1 (%)	4.0204	4.1555	4.4782	3.5165	3.7774	4.5108	2.4843	2.601	2.2957	2.3204	2.7166	2.8439

Figure 5E

**Exp 1, n=3**												
**Samples**	**Ctrl**			**DN-DRP1**			**DN-DRP1 + C75**			**Dn-DRP1 + Baicalin**		
**Citrate M+2 (%)**	**12.1804**	**12.843**	**12.9153**	**8.85565**	**9.67297**	**8.39016**	**10.3307**	**11.6931**	**9.81423**	**8.47467**	**7.83055**	**7.70989**
**Exp 2, n=3**												
**Samples**	**Ctrl**			**DN-DRP1**			**DN-DRP1 + C75**			**DN-DRP1 + Baicalin**		
Citrate M+2 (%)	15.538	15.5193	15.3946	9.63024	9.37033	9.30202	14.9464	15.3512	15.4783	9.78151	9.66176	9.87879

Figure 5F

Samples	Ctrl			DN-DRP1			DN-DRP1 + C75			DN-DRP1N + Baicalin		
Citrate M+2 (%)	12.1021	15.1021	16.8456	6.52899	8.03769	8.61533	10.8119	12.8449	12.8949	8.12429	8.94534	5.98583

Figure 4C, S4B Palmitate (the following experiments were performed on different days and months using cells thawed from different frozen batches over the years)

	OxPhos-DLBCL								BCR-DLBCL							
	**L4**		**Pfeiffer**		**Toledo**		**K4**		**D4**		**Ly1**		**U29**		**D6**	
Citrate M+2 (%)	Con	Drp1 DN	Con	Drp1 DN	Con	Drp1 DN	Con	Drp1 DN	Con	Drp1 DN	Con	Drp1 DN	Con	Drp1 DN	Con	Drp1 DN
**Exp 1**			1.2277	1.0306	2.8509	1.4686			0.6993	0.9093	0.6996					
**Exp 2**	5.3501	2.1752			0.9723	0.6296			0.6054	0.1232	0.7185		0.3757	0.7724		
		2.0006							0.4893							
**Exp 3**	0.6262	0	0.4923	0.3032	0.3032	0.1784			0.6054	0.1232	0.6996	0.6575	0.7856	0.3629		
	0.5786		0.7358						0.4893		0.7185		0.8414			
**Exp 4**	0.529	0.2731									0.8356	0.5811			0.4101	0.2305
	0.4997										0.1985				0.2501	
**Exp 5**	0.6588	0.4651			1.2864	0.7927			0.5911	0.6872					0.3815	0.4319
		0				0				0.2412					0.4	
**Exp 6**			11.808	7.9786	8.4543	4.6563			1.5161	1.5579						
				7.6129						2.7226						

Figure 4C, S4B Hexanoate (the following experiments were performed on different days and n

	OxPhos-DLBCL								BCR-DLBCL							
	**L4**		**Pfeiffer**		**Toledo**		**K4**		**D4**		**Ly1**		**U29**		**D6**	
Citrate M+2 (%)	Con	Drp1 DN	Con	Drp1 DN	Con	Drp1 DN	Con	Drp1 DN	Con	Drp1 DN	Con	Drp1 DN	Con	Drp1 DN	Con	Drp1 DN
**Exp 1**	2.8924	4.6923	11.702	14.376					5.7299	6.507	3.0904	3.2208	5.602	8.9898		
									8.8717		3.1677		10.58			
**Exp 2**			26.026	25.145					26.001	25.107	10.239	15.328	29.069	25.103		
									27.55		13.612		28.298			
**Exp 3**					34.215	35.497	26.27	31.875			21.722	25.646			23.441	30.537
					34.067		29.416				22.647				28.862	
**Exp 4**					33.697	33.652	22.832	20.749			28.633	30.335			29.541	30.19
					36.23		22.179				32.009				32.085	

Expanded View Table 2. Absolute values of 13C incorporation from [U-13C16] palmitate into citrate from primary hepatocytes and DLBCL. Absolute values of tracing experiments prior to normalization. Each experiment is an individual biological replicate.
