# Supplementary material for: Mitochondrial morphology controls fatty acid utilization by changing CPT1 sensitivity to malonyl‐CoA
Source: EMBO J. 2023 Mar 14;42(11):e111901. doi: 10.15252/embj.2022111901 (PMC10233380; doi:10.15252/embj.2022111901)
Supplement: Supplementary file 5 [file EMBJ-42-e111901-s005.pdf]

THE EMBO JOURNAL

Mitochondrial morphology controls fatty acid utilization by changing CPT1 sensitivity to malonyl-CoA

Jennifer Ngo, Dong Wook Choi, Illana A. Stanley, Linsey Stiles, Anthony Molina, Pei-Hsuan Chen, Ana Lako, Isabelle Chiao Han Sung, Rishov Goswami, Min-young Kim, Nathanael Miller, Siyouneh Baghdasarian, Doyeon Kim-Vasquez, Anthony E. Jones, Brett Roach, Vincent Gutierrez, Karel Erion, Ajit S. Divakaruni, Marc Liesa, Nika N. Danial, Orian S. Shirihai
DOI: 10.15252/embj.2022111901

Corresponding author: Orian Shirihai (oshirihai@mednet.ucla.edu) , Nika Danial (Nika_Danial@dfci.harvard.edu)

Review Timeline:

Submission Date:	29th Jun 22
Editorial Decision:	2nd Aug 22
Revision Received:	25th Nov 22
Editorial Decision:	20th Dec 22
Revision Received:	17th Jan 23
Accepted:	2nd Feb 23

Editor: Daniel Klimmeck

Transaction Report:

(Note: With the exception of the correction of typographical or spelling errors that could be a source of ambiguity, letters and reports are not edited. Depending on transfer agreements, referee reports obtained elsewhere may or may not be included in this compilation. Referee reports are anonymous unless the Referee chooses to sign their reports.)

1st Editorial Decision **2nd Aug 2022**

Dear Orian,

Thank you for the submission of your manuscript (EMBOJ-2022-111901) to The EMBO Journal. Please accept my apologies for the delay with the peer-review of your work due to protracted referee input and detailed discussions in the team. Your study has been sent to three referees with expertise in cellular metabolism and mitochondrial biology, and we have received reports from all of them, which I enclose below.

As you will see, the referees acknowledge the potential interest and novelty of your results, although they also express a number of major issues that will have to be conclusively addressed before they can be supportive of publication of your manuscript in The EMBO Journal. In more detail, referee #3 states that the claims on causal linkage between mitochondrial fragmentation and FAO are not sufficiently supported in his/her view, and requests complementary experiments to consolidate your claims (ref#3, pt.1). Further, this expert points to unclear aspects of the differential malonyl-CoA sensitivity data presented (ref#3, pt.2). Reviewer #1 points to additional experiments required to corroborate a general Mfn-dependence of FAO (ref#1, see also ref#2). Further, referee #2 requests specific consideration of the role of peroxisomes for mito-dynamics induced FAO. Finally, all three referees list a number of additional minor issues related to data presentation, complementary controls and discussion of the findings that would have to be addressed to achieve the level of robustness needed for The EMBO Journal.

I judge the comments of the referees to be generally reasonable and given their overall interest, we are in principle happy to invite you to revise your manuscript experimentally to address the referees' comments. I should add that it is EMBO Journal policy to allow only a single round of revision, and acceptance of your manuscript will therefore depend on the completeness of your responses in this revised version. Specifically, the concerns of referee #3 refer to core claims of the current work and should thus be rigorously addressed.

In light of the extensive experimentation requested by the reviewers, I would appreciate if you could contact me during the next weeks via e.g. a video call to discuss your perspective on the comments and potential plan for revisions.

When preparing your letter of response to the referees' comments, please bear in mind that this will form part of the Review Process File, and will therefore be available online to the community. For more details on our Transparent Editorial Process, please visit our website: https://www.embopress.org/page/journal/14602075/authorguide#transparentprocess

As you know, we generally allow three months as standard revision time. As a matter of policy, competing manuscripts published during this period will not negatively impact on our assessment of the conceptual advance presented by your study. However, we request that you contact the editor as soon as possible upon publication of any related work, to discuss how to proceed. Should you foresee a problem in meeting this three-month deadline, please let us know in advance and we may be able to grant an extension.

When submitting your revised manuscript, please carefully review the instructions below.

Thank you for the opportunity to consider your work for publication.
I look forward to your revision.

Best regards,

Daniel

Daniel Klimmeck, PhD
Senior Editor
The EMBO Journal

Instruction for the preparation of your revised manuscript:

1) a .docx formatted version of the manuscript text (including legends for main figures, EV figures and tables). Please make sure that the changes are highlighted to be clearly visible.

2) individual production quality figure files as .eps, .tif, .jpg (one file per figure).

3) a .docx formatted letter INCLUDING the reviewers' reports and your detailed point-by-point response to their comments. As part of the EMBO Press transparent editorial process, the point-by-point response is part of the Review Process File (RPF), which will be published alongside your paper.

4) a complete author checklist, which you can download from our author guidelines (https://wol-prod-cdn.literatumonline.com/pb-assets/embo-site/Author Checklist%20-%20EMBO%20J-1561436015657.xlsx). Please insert information in the checklist that is also reflected in the manuscript. The completed author checklist will also be part of the RPF.

5) Please note that all corresponding authors are required to supply an ORCID ID for their name upon submission of a revised manuscript.

6) It is mandatory to include a 'Data Availability' section after the Materials and Methods. Before submitting your revision, primary datasets produced in this study need to be deposited in an appropriate public database, and the accession numbers and database listed under 'Data Availability'. Please remember to provide a reviewer password if the datasets are not yet public (see https://www.embopress.org/page/journal/14602075/authorguide#datadeposition).
In case you have no data that requires deposition in a public database, please state so in this section. Note that the Data Availability Section is restricted to new primary data that are part of this study.
*** Note - All links should resolve to a page where the data can be accessed. ***

7) Our journal encourages inclusion of *data citations in the reference list* to directly cite datasets that were re-used and obtained from public databases. Data citations in the article text are distinct from normal bibliographical citations and should directly link to the database records from which the data can be accessed. In the main text, data citations are formatted as follows: "Data ref: Smith et al, 2001" or "Data ref: NCBI Sequence Read Archive PRJNA342805, 2017". In the Reference list, data citations must be labeled with "[DATASET]". A data reference must provide the database name, accession number/identifiers and a resolvable link to the landing page from which the data can be accessed at the end of the reference. Further instructions are available at .

8) We would also encourage you to include the source data for figure panels that show essential data. Numerical data can be provided as individual .xls or .csv files (including a tab describing the data). For 'blots' or microscopy, uncropped images should be submitted (using a zip archive or a single pdf per main figure if multiple images need to be supplied for one panel). Additional information on source data and instruction on how to label the files are available at .

9) We replaced Supplementary Information with Expanded View (EV) Figures and Tables that are collapsible/expandable online (see examples in https://www.embopress.org/doi/10.15252/embj.201695874). A maximum of 5 EV Figures can be typeset. EV Figures should be cited as 'Figure EV1, Figure EV2" etc. in the text and their respective legends should be included in the main text after the legends of regular figures.

- For the figures that you do NOT wish to display as Expanded View figures, they should be bundled together with their legends in a single PDF file called *Appendix*, which should start with a short Table of Content. Appendix figures should be referred to in the main text as: "Appendix Figure S1, Appendix Figure S2" etc. See detailed instructions regarding expanded view here: .

- Additional Tables/Datasets should be labelled and referred to as Table EV1, Dataset EV1, etc. Legends have to be provided in a separate tab in case of .xls files. Alternatively, the legend can be supplied as a separate text file (README) and zipped together with the Table/Dataset file.

10) When assembling figures, please refer to our figure preparation guideline in order to ensure proper formatting and readability in print as well as on screen:
http://bit.ly/EMBOPressFigurePreparationGuideline

Please remember: Digital image enhancement is acceptable practice, as long as it accurately represents the original data and conforms to community standards. If a figure has been subjected to significant electronic manipulation, this must be noted in the figure legend or in the 'Materials and Methods' section. The editors reserve the right to request original versions of figures and the original images that were used to assemble the figure.

11) For data quantification: please specify the name of the statistical test used to generate error bars and P values, the number (n) of independent experiments (specify technical or biological replicates) underlying each data point and the test used to calculate p-values in each figure legend. The figure legends should contain a basic description of n, P and the test applied. Graphs must include a description of the bars and the error bars (s.d., s.e.m.).

Further information is available in our Guide For Authors: https://www.embopress.org/page/journal/14602075/authorguide

We realize that it is difficult to revise to a specific deadline. In the interest of protecting the conceptual advance provided by the work, we recommend a revision within 3 months (31st Oct 2022). Please discuss the revision progress ahead of this time with the editor if you require more time to complete the revisions. :

Referee #1:

Ngo et al has studied the effect of mitochondrial elongation and fragmentation on fatty acid oxidation (FAO). They present data with genetic perturbations of both Mfn2 and Drp1 in several cell types, which support that elongation and shortening of mitochondria is associated with decreased and increased FAO, respectively. This phenotype is also connected with hepatic gluconeogenesis, insulin secretion and cancer cell metabolism. Evidence is provided that mitochondrial elongation enhances the MalonylCoA-mediated inhibition of CPT1. The study is important and interesting. I think the Authors have to fill some gaps to make the ms available for EMBO J.
Fig1A-C: To exclude that the FAO was not constrained by an oxphos defect in some genetic perturbations, including the DN-Drp1, showing the OCR with succinate would be helpful
The size of the FAO responses in the time courses of Fig1B does not seem to match with the FAO numbers listed in C. Please explain how the values in C were calculated

Fig2 Insulin secretion gluconeogenesis are several steps downstream of FAO. Showing the results is useful but these are not the strongest evidence for the mitochondrial shape-FAO link.
Fig2G Isn't the ceiling of insulin production reached by 15mM glucose alone?

Fig 1,3,4,5 many bar charts show normalized values. It would be important to show the absolute values that were used to normalize and to better explain how the normalization was done.
In Fig4 the absolute values for Pal and Hex have to be stated
Fig5A Please show the absolute values of CPT1 expression or maximal activity for each genetic condition.
Fig5EF please clarify if mitochondrial morphology was altered by 3h of treatment with C75 and Baicalin.

Although the Authors mention the multiple functions of Mfn2, which limit the interpretation of Mfn2 perturbations in terms of mitochondrial elongation but they don't test in any experiments Mfn1 which is central to mitochondrial fusion in several of the paradigms used here.

The discussion should include Tondera et al 2009 EMBO J, Gomes et al 2011 NCB, Chen et al 2005 JCB especially those published results which seem to diverge from the present suggestions. For example in Gomes et al 2011 fasting that likely includes increased FAO was associated with mitochondrial elongation in the liver.

There are several typos, the text requires a careful revision.

Referee #2:

The manuscript by Ngo and colleagues addresses the role of mitochondrial dynamics in the metabolic fuel choice. This is a very novel question, that has broad implications for cellular and systemic metabolism. The manuscript is well written, and the experiments are carried out in a logical manner. The conclusions that the authors make are well substantiated by the data. The data is presented clearly (albeit it is not always clear how many biological replicates were used).
I have two criticisms. My one major criticism is that on a paper that discusses the use of long-chain fatty acids as metabolic fuel, there is no mention of peroxisomes, which are necessary for the initial steps of beta-oxidation of these FAs. Furthermore, several proteins that regulate mitochondrial dynamics also regulate peroxisomal dynamics. In fairness, most of the conclusions by the authors are in models lacking MFN2 (which is, to the current knowledge, not involved in peroxisomal dynamics), and showing mitochondrial fragmentation as a consequence of lower mitochondrial fusion. It would be important to know if the manipulations on mitochondrial fission/fusion have repercussions on peroxisomes, possibly affecting FA oxidation. The other criticism is that MFN2 has been associated with mitochondria-ER contact sites, so it would be valuable to have one experiment in which MFN1 (instead of MFN2) is decreased, to show that the effect is not dependent on the MFN2 effect on mito-ER contacts.
The discussion could be shortened a bit for simplicity.
In page 5 of the pdf (there's no page numbers), in the section "Mitochondrial fragmentation stimulates gluconeogenesis in primary hepatocytes", lines 8-9 of this section, the sentence "Forced mitochondrial fragmentation ... via siDRP1 or DN-DRP1" - I believe that this would cause hyperfusion, not fragmentation, please correct accordingly.

Nuno Raimundo

Referee #3:

The manuscript proposes a mechanistic link between mitochondrial fragmentation and increased fatty acid oxidation, which would be due to decreased inhibition of CPT1 by malonyl-CoA.

1. I must object to the interpretation of the experiments of Figure 1B, which is the core of the study. The palmitoyl-CoA supported, etomoxir-sensitive oxygen consumption rate stimulated by ADP is the same for elongated and fragmented mitochondria. The difference is rather due to a higher rate of respiration in the presence of etomoxir. The key point, however, is that FCCP-stimulated respiration is basically the same, or even higher for fragmented mitochondria (check the area under the curves after addition of FCCP). I suspect that the effect of fragmentation is actually on the rate of ATP synthesis, which would also explain the reduced gluconeogenesis reported in panels H-J. A control using substrates of the Krebs cycle could have been extremely useful in this respect, and I am surprised that it has not been performed (or provided).

2. The difference in sensitivity to malonyl-CoA is not convincing. I recommend that the authors read the original method, which was developed by Bieber et al in 1972, Anal. Biochem. 50, 509-518 rather than relying on second-hand descriptions and missing the proper references. For example, there is no CPT1 assay in the quoted Shin, SY et al 2006 paper, the correct reference is to another Shin ..... Shin ES et al. (2006). Positive regulation of hepatic carnitine palmitoyl transferase 1A (CPT1A) activities by soy isoflavones and L-carnitine. European Journal of Nutrition 45, 159-164. What I would like to see is a full titration with malonyl-CoA (CoASH can also be generated by hydrolases and this is the only way to make sure that activity of CPT1 is being measured) normalized for total protein content. They state that results were normalized to CPT1 expression levels by immunoblots, which are never shown. What is the variability of expression like? How was the quantification carried out?

3. C75 was introduced as a chemically stable inhibitor of mammalian fatty acid synthase in 2000 by Kuhajda et al., Synthesis and antitumor activity of an inhibitor of fatty acid synthase, Proc. Natl. Acad. Sci. USA 97 (2000) 3450-3454 and later shown to activate CPT, Nicot et al (2004) BBRC 325, 660-664. Therefore there is no mention of it in the indicated reference by Bremer et al 1985.

4. About baicailin, in the reference list there is no Dai et al (2019), which is mentioned in the last paragraph or the results. If this was meant to cite Dai et al (2019) Baicalin protects human retinal pigment epithelial cell lines against high glucose-induced cell injury by up-regulation of microRNA-145, Exp Mol Pathol 106, 123-130, this paper has been retracted.

5. I could not find any evidence in the literature that baicalin acts on CPT1. Rather, it may act by suppressing NFkB and p38 MAPK, Wu et al. (2018) Baicalin alleviates atherosclerosis by relieving oxidative stress and inflammatory responses via inactivating the NF-κB and p38 MAPK signaling pathways, Biomed Pharmacother 97, 1673-1679. Thus, it may not be surprising (or informative) that baicalin had no effects on fatty acid oxidation and this point and all the speculations on mechanism appear untenable.

Referee #1:

Ngo et al has studied the effect of mitochondrial elongation and fragmentation on fatty acid oxidation (FAO). They present data with genetic perturbations of both Mfn2 and Drp1 in several cell types, which support that elongation and shortening of mitochondria is associated with decreased and increased FAO, respectively. This phenotype is also connected with hepatic gluconeogenesis, insulin secretion and cancer cell metabolism. Evidence is provided that mitochondrial elongation enhances the MalonylCoA-mediated inhibition of CPT1. The study is important and interesting. I think the Authors have to fill some gaps to make the ms available for EMBO J.

Fig1A-C: To exclude that the FAO was not constrained by an oxphos defect in some genetic perturbations, including the DN-Drp1, showing the OCR with succinate would be helpful

We thank the reviewer for this comment. Expanded View Figure 1B in the revised manuscript in the revised version includes measurements of OCR with succinate in siCtrl, mi*MFN2*, and DN-DRP1 HepG2 cells, which are also shown below in **Figure R1.1** for ease of reference**.** Analysis of succinate-driven respiration demonstrates respiratory rates that are all above the values of FA-driven respiration, confirming that the respiratory chain function is not limiting FA-driven respiration at all conditions tested.

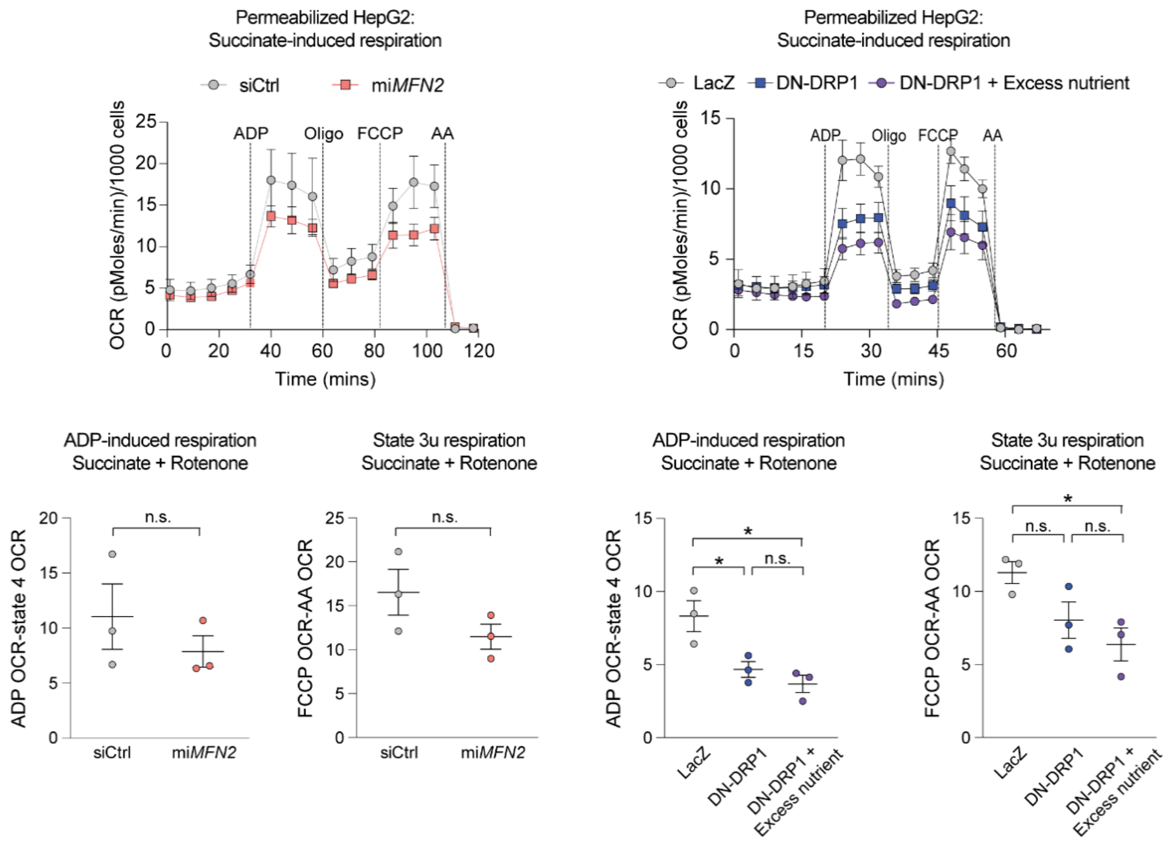

Figure R1.1. *Succinate-induced respiration in permeabilized* siCtrl, mi*MFN2*, and DN-DRP1 *HepG2 cells. Traces and data are aggregate of 3 biological replicates with 6 technical repeats.*

Figure R1.2B below further demonstrates that FAO is not constrained by general defect in OXPHOS. Indeed, mi*MFN2* increased FAO and thus the decrease in succinate oxidation induced by mi*MFN2* is not reflecting a general constraint on OXPHOS.

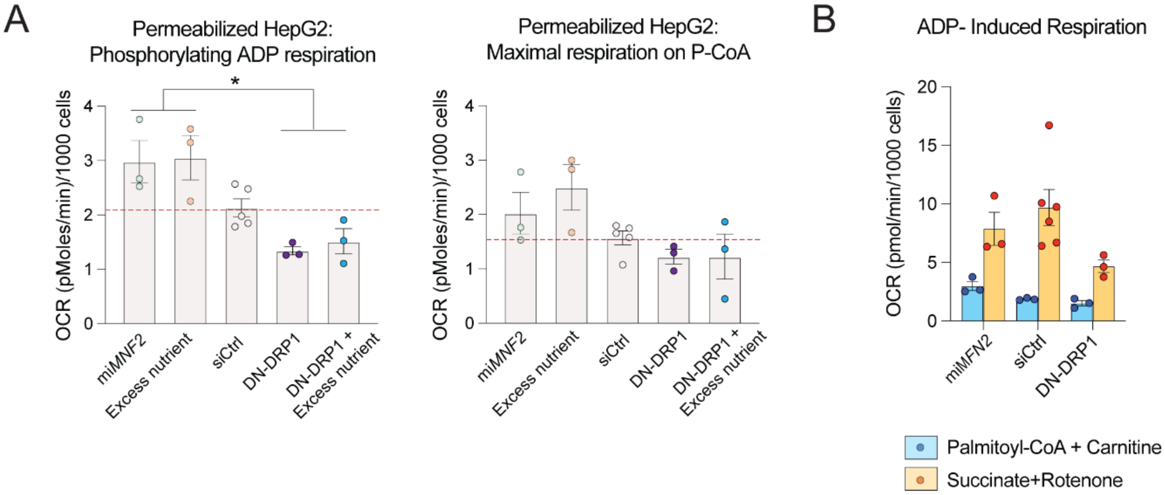

Figure R1.2. *(A) Phosphorylating ADP (state 3) and Maximal OCR values induced by palmitoyl-CoA. Conditions that lead to increased mitochondrial fragmentation maintain higher phosphorylating ADP OCR and Maximal OCR versus conditions favoring mitochondrial elongation. (B) Phosphorylating ADP respiration in response to palmitoyl-CoA or succinate.*

The size of the FAO responses in the time courses of Fig1B does not seem to match with the FAO numbers listed in C. Please explain how the values in C were calculated

Thank you for pointing this out and apologies for the lack of clarity on this. The individual FAO responses included in Figure1B represent the mean values of 3 biological replicates. Each data point on the graph is the averaged value from 6 technical repeats in one biological replicate. Values shown in Figure 1C are the aggregate of state 3 respiration (ADP phosphorylation) values of n=3 biological replicates of corresponding conditions. These individual values are now provided in Expanded View Table 1 of the revised manuscript and also shown below as **Table R1.1** for ease of reference**.** We have also revised the legend for Figure 1 for clarity.

Treatment Group	Aspect Ratio; n=3 (Fig 1B)	Etomoxir-Sensitive OCR; n=3 (Fig 1B)	Average Aspect Ratio Values (Fig 1C)	Average Etomoxir-Sensitive OCR Values (Fig 1C)
Excess nutrient	1.896	3.583	2.183	3.056
	2.345	3.333		
	2.308	2.250		
mi*MFN2*	2.382	3.758	2.371	2.983
	2.390	2.666		
	2.340	2.524		
siCtrl	2.743	1.786	2.656	1.871
	2.654	1.978		
	2.571	1.849		
DN-DRP1	2.816	1.110	2.906	1.516
	2.955	1.910		
	2.947	1.528		
DN-DRP1+ Excess nutrient	2.659	1.262	2.735	1.344
	2.751	1.495		
	2.795	1.275		

Table R1.1. *Mean values of mitochondrial aspect ratio and etomoxir-sensitive respiration from 3 biological replicates (column 1 and 2). Column 3 and 4 are mean values of the aggregate of 3 biological replicates presented in column 1 and 2.*

Fig2 Insulin secretion gluconeogenesis are several steps downstream of FAO. Showing the results is useful but these are not the strongest evidence for the mitochondrial shape-FAO link.

We thank the reviewer for giving us the opportunity to clarify this issue. The question we aimed to address by including these parameters is whether the effects we have observed on FAO are physiologically significant, or are they fully compensated for by the cells. This is especially interesting when the effect is not a complete abrogation of FAO, but rather partial. Does it translate into a physiologically meaningful outcome? To address this question, we examined processes that are known to be dependent on FAO in the hepatocyte and the β-cells. Insulin secretion in β-cells and gluconeogenesis in hepatocytes are two physiological relevant, functional outcomes of FAO in these cell types. As such, these readouts serve as critical tests whether the mitochondrial shape-FAO link we report has specific physiological consequences despite being several steps downstream of FAO. Our findings that genetic manipulations, which lead to increased mitochondrial fragmentation, are also required for biological functions that are normally driven by FAO in each of these cell types indicate the physiological relevance of this link. We feel this is an important strength of the manuscript.

We have rephrased the following text as clarification to the discussion section on page 14 line 440-443:

"The finding that ~~mitochondrial fragmentation drives FAO and regulates~~ these diverse cellular phenotypes are altered as a consequence of the actions of mitochondrial morphology on fatty

acid utilization supports a central role for mitochondrial morphology as a basic biologic mechanism controlling FAO that was not fully appreciated."

Fig2G Isn't the ceiling of insulin production reached by 15mM glucose alone?

We thank the reviewer for this comment. Indeed, 15 mM glucose can stimulate insulin secretion at its maximal rates. We did not expect any changes to maximal insulin secretion. Our goal was to examine the effect on basal insulin secretion (3 mM glucose), a parameter that has been shown to be associated with FA utilization. Previous studies by Marc Prentki and Barbara Corkey describe that early on during the development of diabetes, islets start secreting insulin in response to fatty acids, even at low glucose concentrations (Erion *et al*, 2015; Merrins *et al*, 2022). This phenomenon of FA-stimulated secretion at low glucose concentration was previously hypothesized to be connected to CPT1 changes, but the mechanism remained elusive. The mechanism we reveal in this manuscript can directly explain how islets become FA-sensitive during the development of obesity in the pre-diabetic period, when MFN2 levels in the islet decline.

Fig 1,3,4,5 many bar charts show normalized values. It would be important to show the absolute values that were used to normalize and to better explain how the normalization was done.

In Fig4 the absolute values for Pal and Hex have to be stated.

We appreciate this point. We have included the requested absolute values and the values utilized for normalization in Expanded View Table 2 as a form of excel sheet and below as **Table R1.2** for ease of reference.

Figure 1E

Samples	siCtrl			mi*MFN2*		
Citrate M+2 (%)	16.7353	4.72748	21.1455	21.8973	5.75999	24.5577

Figure 1F

Samples	Ctrl			MFN1 KO			Ctrl+Eto			MFN1 KO+Eto		
Citrate M+2 (%)	16.9913	16.595	16.3045	18.1227	20.7772	21.0787	3.2363	2.5327	1.8654	1.9463	2.797	3.0757

Figure 1G

Samples	Ctrl			MFN1 OE		
Citrate M+2 (%)	38.4347	39.0319	37.54	29.3822	27.2629	27.0902

Figure 1H

Samples	Ctrl			si*DRP1*		
Citrate M+2 (%)	43.603	44.275	45.65	39.9	39.729	38.093

Figure 1I

Samples	siCtrl			DN-DRP1		
Citrate M+2 (%)	31.288	31.328	31.674	26.246	26.554	24.592

Figure 3F

U13C-Pal	OxPhos-DLBCL											BCR-DLBCL											
Samples	Peiffer			Peiffer + MFN2			Toledo			Toledo + MFN2		DHL4			DHL4 + MFN2			DHL6			DHL6 + MFN2		
Citrate M+2 (%)	15.732	14.614	14.375	12.928	12.526	12.418	3.114	2.8498	3.4552	1.7113	1.5117	2.8525	2.0703	1.5948	2.6243	4.3198	4.1	0.4657	0.9498	1.1038	0.4695	0.8026	0.3249	
1-13C Hex	OxPhos-DLBCL											BCR-DLBCL											
Samples	Peiffer			Peiffer + MFN2			Toledo			Toledo + MFN2			DHL4			DHL4 + MFN2			DHL6			DHL6 + MFN2		
Citrate M+1 (%)	7.1892	6.6779	6.5684	7.0277	6.5217	6.4245	7.0303	6.5793	7.9769	7.0304	6.4339	7.8007	5.0327	4.9047	2.7422	4.5124	7.4276	7.0487	3.4921	2.5342	1.9524	3.2171	5.2884	5.0193

Figure 4D

Samples	Control			DN-DRP1			Control			DN-DRP1		
Citrate M+2 (%)	31.288	31.328	31.674	26.246	26.554	24.592	1.3251	1.5824	1.1691	1.2443	1.4679	1.5037
Citrate M+1 (%)	4.3838	4.2946	4.4784	4.5035	4.7682	6.7234	23.261	24.326	23.762	22.905	23.552	24.458

Figure 4E

	U13C-Pal						1-13C Hex					
Samples	siCtrl			si*DRP1*			siCtrl			si*DRP1*		
Citrate M+2 (%)	42.891	41.945	42.341	37.598	38.933	37.693	43.603	44.275	45.65	39.9	39.729	38.093
Citrate M+1 (%)	4.0204	4.1555	4.4782	3.5165	3.7774	4.5108	2.4843	2.601	2.2957	2.3204	2.7166	2.8439

Figure 5E

**Exp 1, n=3**												
**Samples**	Ctrl			DN-DRP1			DN-DRP1 + C75			Dn-DRP1 + Baicalin		
**Citrate M+2 (%)**	12.1804	12.843	12.9153	8.85565	9.67297	8.39016	10.3307	11.6931	9.81423	8.47467	7.83055	7.70989
**Exp 2, n=3**												
**Samples**	Ctrl		DN-DRP1			DN-DRP1 + C75			DN-DRP1 + Baicalin			
Citrate M+2 (%)	15.538	15.5193	15.3946	9.63024	9.37033	9.30202	14.9464	15.3512	15.4783	9.78151	9.66176	9.87879

Figure 5F

Samples	Ctrl			DN-DRP1			DN-DRP1 + C75			DN-DRP1N + Baicalin		

Citrate M+2 (%)	12.1021	15.1021	16.8456	6.52899	8.03769	8.61533	10.8119	12.8449	12.8949	8.12429	8.94534	5.98583

Figure 4C, S4B Palmitate (the following experiments were performed on different days and months using cells thawed from different frozen batches over the years)

	OxPhos-DLBCL								BCR-DLBCL							
	L4		Pfeiffer		Toledo		K4		D4		Ly1		U29		D6	
Citrate M+2 (%)	Con	Drp1 DN	Con	Drp1 DN	Con	Drp1 DN	Con	Drp1 DN	Con	Drp1 DN	Con	Drp1 DN	Con	Drp1 DN	Con	Drp1 DN
**Exp 1**			1.2277	1.0306	2.8509	1.4686			0.6993	0.9093	0.6996					
**Exp 2**	5.3501	2.1752			0.9723	0.6296			0.6054	0.1232	0.7185		0.3757	0.7724		
		2.0006							0.4893							
**Exp 3**	0.6262	0	0.4923	0.3032	0.3032	0.1784			0.6054	0.1232	0.6996	0.6575	0.7856	0.3629		
	0.5786		0.7358						0.4893		0.7185		0.8414			
**Exp 4**	0.529	0.2731									0.8356	0.5811			0.4101	0.2305
	0.4997										0.1985				0.2501	
**Exp 5**	0.6588	0.4651			1.2864	0.7927			0.5911	0.6872					0.3815	0.4319
		0				0				0.2412					0.4	
**Exp 6**			11.808	7.9786	8.4543	4.6563			1.5161	1.5579						
				7.6129						2.7226						

Figure 4C, S4B Hexanoate (the following experiments were performed on different days an

	OxPhos-DLBCL								BCR-DLBCL							
	L4		Pfeiffer		Toledo		K4		D4		Ly1		U29		D6	
Citrate M+2 (%)	Con	Drp1 DN	Con	Drp1 DN	Con	Drp1 DN	Con	Drp1 DN	Con	Drp1 DN	Con	Drp1 DN	Con	Drp1 DN	Con	Drp1 DN
**Exp 1**	2.8924	4.6923	11.702	14.376					5.7299	6.507	3.0904	3.2208	5.602	8.9898		
									8.8717		3.1677		10.58			
**Exp 2**			26.026	25.145					26.001	25.107	10.239	15.328	29.069	25.103		
									27.55		13.612		28.298			
**Exp 3**					34.215	35.497	26.27	31.875			21.722	25.646			23.441	30.537
					34.067		29.416				22.647				28.862	
**Exp 4**					33.697	33.652	22.832	20.749			28.633	30.335			29.541	30.19
					36.23		22.179				32.009				32.085	

Table R1.2. *Absolute values utilized for normalization*.

Fig5A Please show the absolute values of CPT1 expression or maximal activity for each genetic condition.

We have included the absolute values of CPT1 expression and immunoblots of CPT1 for each genetic condition in Expanded View Figures 1C-E and Expanded View Figures 6A-B of the revised manuscript and also provided below as **Figure R1.3** for ease of reference.

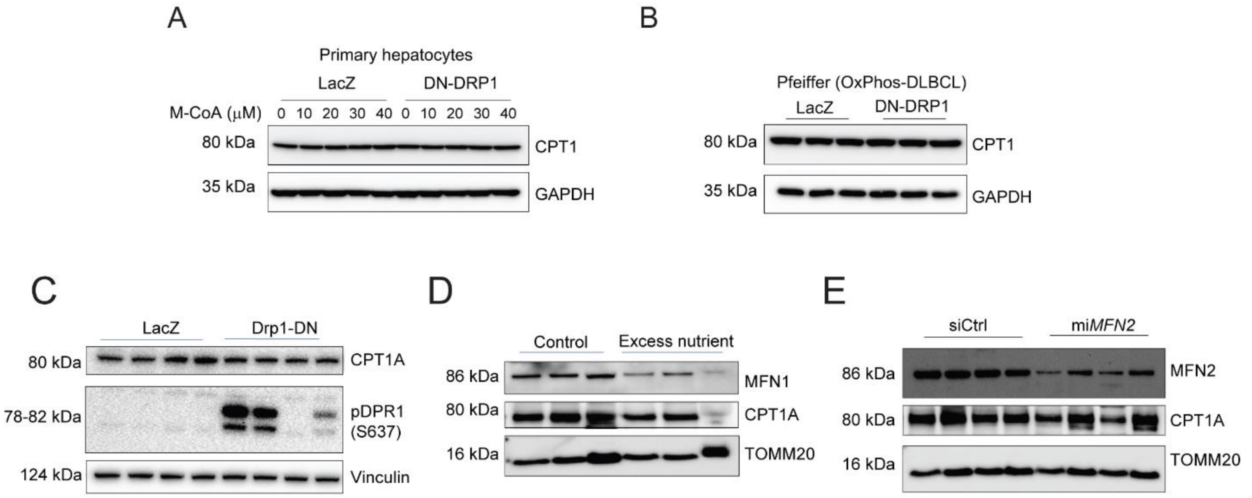

Figure R1.3*. Genetic or nutrient-induced perturbations of mitochondrial morphology do not alter CPT1 protein levels.*

Fig5EF please clarify if mitochondrial morphology was altered by 3h of treatment with C75 and Baicalin.

We have addressed this point with new data, which is now included in Expanded View Figure 6F-G and also provided below in **Figure R1.4** for ease of reference. Briefly, mitochondrial morphology is not altered following 3h treatment of HepG2 cells with 80 μM of C75 or 100 μM of Baicalin.

We have now included the following statement on page 12 line 380-382: “We also performed imaging studies to ensure mitochondrial morphology or membrane potential was not altered by C75 and baicalin (Expanded View Figures 6F-G).”

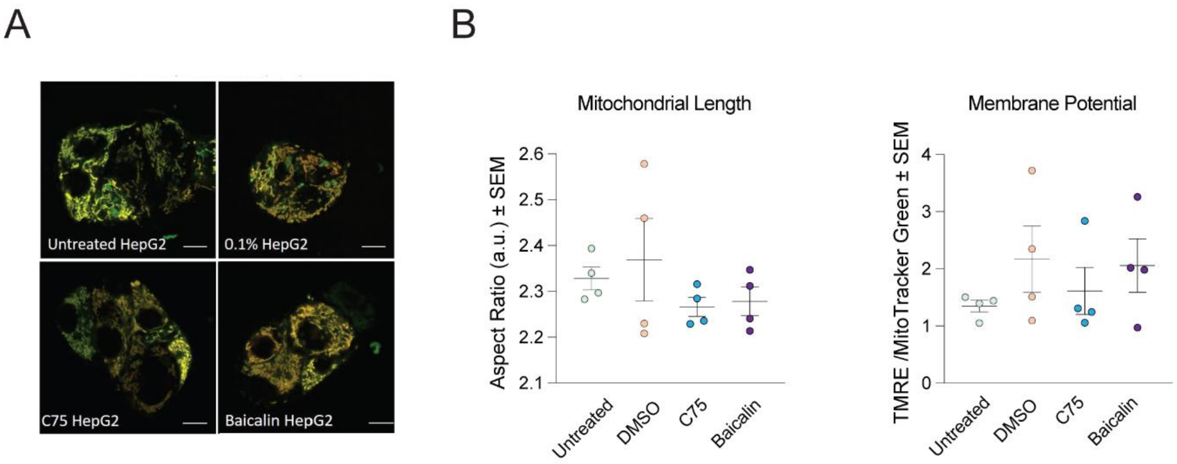

Figure R1.4. *C75 or baicalin do not alter mitochondrial morphology. Representative confocal images of mitochondria (A) and quantification of mitochondrial aspect ratio and membrane potential (B) shown for HepG2 cells following 3h of treatment with 80 μM of C75 or 100 μM of Baicalin. Cumulative data from 4 biological replicates are shown, each with 40 cells per condition.*

Although the Authors mention the multiple functions of Mfn2, which limit the interpretation of Mfn2 perturbations in terms of mitochondrial elongation but they don't test in any experiments Mfn1 which is central to mitochondrial fusion in several of the paradigms used here.

We thank the reviewer for this point, which we have addressed with new data shown in Figures 1F, 1G, 1L and Expanded View Figure 1F of the revised manuscript and also shown below as **Figure R1.5** for ease of reference. *MFN1* overexpression (*MFN1* OE) in primary hepatocytes reduces FAO and gluconeogenesis (**Figure R1.5A-B**). Additionally, *MFN1* KO in HepG2 cells confirmed both mitochondrial fragmentation and increases in FAO (**Figure R1.5C-D**). The complementary results from both MFN2 and MFN1 manipulations further support our findings that mitochondrial FAO is influenced by mitochondrial morphology.

We have also included the following statement on page 6 line 187-190: "Primary hepatocytes subjected to MFN1 or 2 overexpression showed a significant diminution of the gluconeogenic response to lactate and pyruvate (Figures 1L-M). This is consistent with diminished FAO under conditions where mitochondrial architecture is hyper-elongated (Figures 1G-I and Expanded View Table 2)."

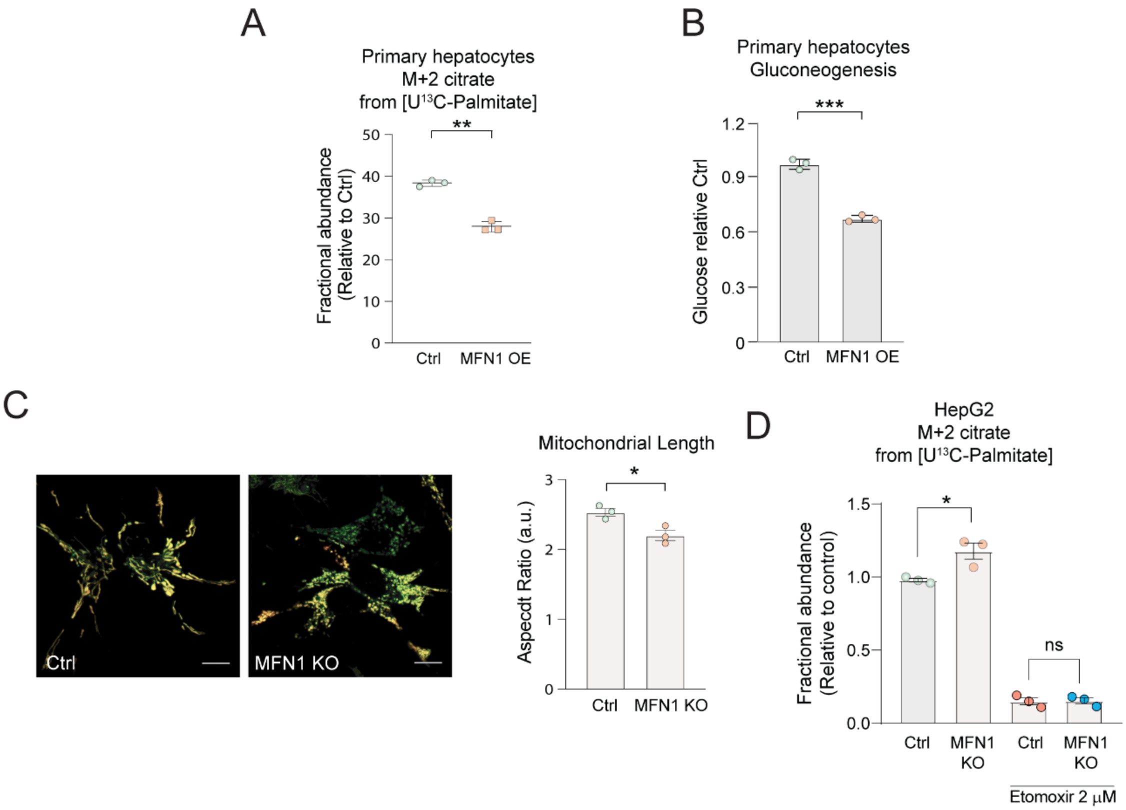

Figure R1.5. *MFN1 overexpression in primary hepatocytes reduces palmitate incorporation into the TCA cycle (A) and reduces gluconeogenesis (B). MFN1 KO in HepG2 cells induces mitochondrial fragmentation (C) and increases palmitate incorporation into the TCA cycle (D).*

The discussion should include Tondera et al 2009 EMBO J, Gomes et al 2011 NCB, Chen et al 2005 JCB especially those published results which seem to diverge from the present suggestions. For example in Gomes et al 2011 fasting that likely includes increased FAO was associated with mitochondrial elongation in the liver.

We thank the reviewer for this comment. We have added these references and described how their findings both align to and diverge from our findings in the discussion section.

There are several typos, the text requires a careful revision.

We have corrected the typos.

Referee #2:

The manuscript by Ngo and colleagues addresses the role of mitochondrial dynamics in the metabolic fuel choice. This is a very novel question, that has broad implications for cellular and systemic metabolism. The manuscript is well written, and the experiments are carried out in a logical manner. The conclusions that the authors make are well substantiated by the data. The data is presented clearly (albeit it is not always clear how many biological replicates were used).

We thank the reviewer and appreciate their positive evaluation of our work. We have revised the manuscript to clarify biological replicates as requested.

I have two criticisms. My one major criticism is that on a paper that discusses the use of long-chain fatty acids as metabolic fuel, there is no mention of peroxisomes, which are necessary for the initial steps of beta-oxidation of these FAs. Furthermore, several proteins that regulate mitochondrial dynamics also regulate peroxisomal dynamics. In fairness, most of the conclusions by the authors are in models lacking MFN2 (which is, to the current knowledge, not involved in peroxisomal dynamics), and showing mitochondrial fragmentation as a consequence of lower mitochondrial fusion. It would be important to know if the manipulations on mitochondrial fission/fusion have repercussions on peroxisomes, possibly affecting FA oxidation.

We thank the reviewer for bringing up the important point about peroxisomes. Peroxisomes oxidize very long chain (>C20) fatty acids, dicarboxylic fatty acids, and 2-methyl-branched fatty acids (Reddy & Hashimoto, 2001). Notably, peroxisomal β-oxidation is not regulated by malonyl-CoA. Considering that fatty acid chain length dictates, in part, mitochondrial vs peroxisomal FAO, we used hexanoate (6 carbons) and palmitate or palmitoyl-CoA (16 carbons) in our assays. These fatty acids are not typically oxidized by peroxisomes, but Violante *et al*. reported that peroxisomes can oxidize palmitate when mitochondrial FAO is inhibited (Violante *et al*, 2019). This would predict increased OCR in the presence of etomoxir if peroxisomes had such an effect in our experimental system, however, we do not see an increase in OCR. Additionally, in the event peroxisome may contribute to mitochondrial FAO, these contributions would be observed in the non-mitochondrial portion of OCR in seahorse assays, but we have subtracted non-mitochondrial OCR from the values we report here.

To further address this comment, we have included the following statement on page 13 line 423-427 of the revised manuscript: “Throughout the above respirometry assays, we excluded a contribution from peroxisomes by measuring the portion of OCR that is sensitive to etomoxir and thus reporting on mitochondrial FAO. This is especially relevant because peroxisomes can also utilize acyl-carnitines as substrates (Violante *et al*, 2019; Reddy & Hashimoto, 2001), which would otherwise confound interpretation of these assays.”

The other criticism is that MFN2 has been associated with mitochondria-ER contact sites, so it would be valuable to have one experiment in which MFN1 (instead of MFN2) is decreased, to show that the effect is not dependent on the MFN2 effect on mito-ER contacts.

We thank the reviewer for this point, which is now addressed with new data. We show that MFN1 overexression (MFN1 OE) in primary hepatocytes reduces FAO and gluconeogenesis. Conversely, loss of MFN1 in HepG2 cells increases FAO. These data are included in Figures 1F, 1G, and 1L of the revised manuscript and are also shown below as **Figure R2.1** for ease of reference. The complementary results from both MFN2 and MFN1 manipulations further support our findings that mitochondrial FAO is influenced by mitochondrial morphology.

We have also included the following statement on page 6 line 187-190: “Primary hepatocytes subjected to MFN1 or 2 overexpression showed a significant diminution of the gluconeogenic response to lactate and pyruvate (Figures 1L-M). This is consistent with diminished FAO under conditions where mitochondrial architecture is hyper-elongated (Figures 1G-I and Expanded View Table 2).”

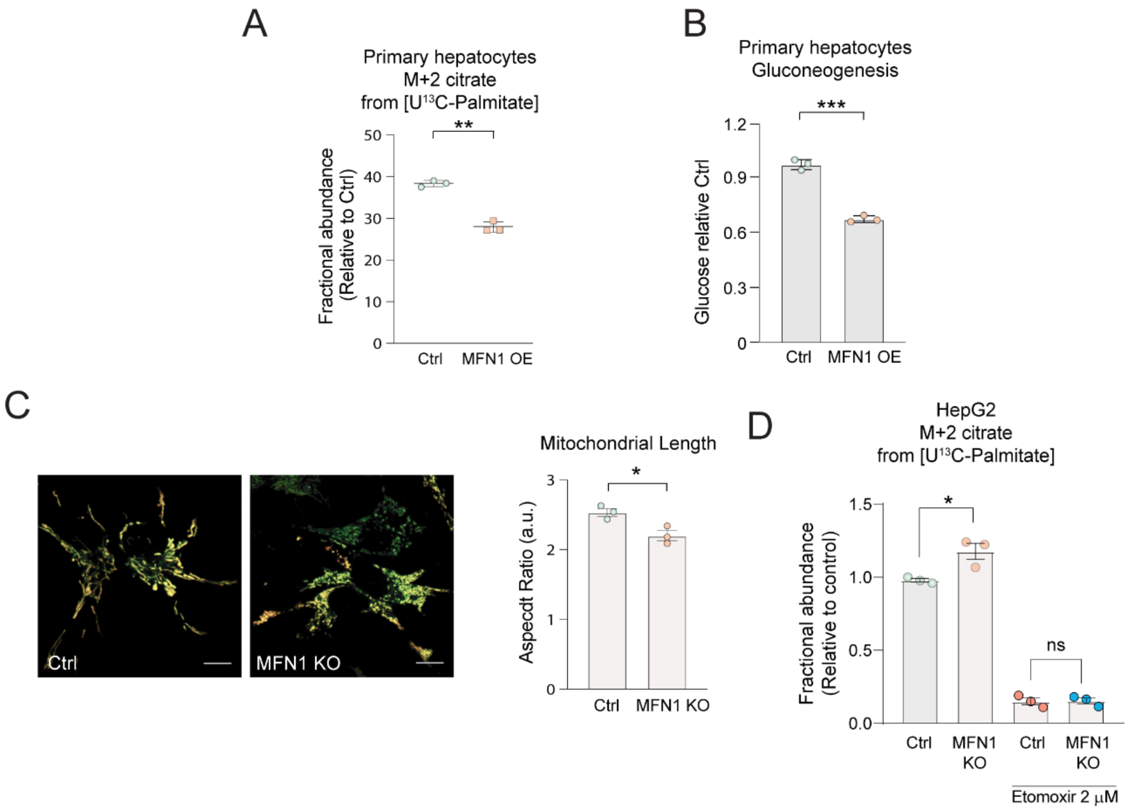

Figure R2.1. *MFN1 overexpression in primary hepatocytes reduces palmitate incorporation into the TCA cycle (A) and reduces gluconeogenesis (B). MFN1 KO in HepG2 cells induces mitochondrial fragmentation (C) and increases palmitate incorporation into the TCA cycle (D).*

The discussion could be shortened a bit for simplicity.

We have revised the discussion to improve clarity, while incorporating additional points that were raised by all reviewers.

In page 5 of the pdf (there's no page numbers), in the section "Mitochondrial fragmentation stimulates gluconeogenesis in primary hepatocytes", lines 8-9 of this section, the sentence "Forced mitochondrial fragmentation ... via siDRP1 or DN-DRP1" - I believe that this would cause hyperfusion, not fragmentation, please correct accordingly.

We thank the reviewer for pointing this out and have corrected the sentence accordingly.

Referee #3:

The manuscript proposes a mechanistic link between mitochondrial fragmentation and increased fatty acid oxidation, which would be due to decreased inhibition of CPT1 by malonyl-CoA.

I must object to the interpretation of the experiments of Figure 1B, which is the core of the study. The palmitoyl-CoA supported, etomoxir-sensitive oxygen consumption rate stimulated by ADP is the same for elongated and fragmented mitochondria. The difference is rather due to a higher rate of respiration in the presence of etomoxir. The key point, however, is that FCCP-stimulated respiration is basically the same, or even higher for fragmented mitochondria (check the area under the curves after addition of FCCP). I suspect that the effect of fragmentation is actually on the rate of ATP synthesis, which would also explain the reduced gluconeogenesis reported in panels H-J.

We thank the reviewer for this point. We would like to clarify that the values presented are not a measurement of area under the curve. State 3 respiration was assessed by the difference of substrate-induced OCR minus OCR in the presence of FAO-inhibitor using the following equation:

Phosphorylating respiration on Palmitoyl-CoA = (Palmitoyl-CoA + Carnitine + ADP OCR) – (Palmitoyl-CoA + Carnitine + Etomoxir + ADP OCR)

Etomoxir inhibits fatty acid oxidation by irreversibly inhibiting CPT1a. The experimental group denoted as ‘vehicle’ does not have saturating levels of fuel to oxidize and contains only endogenous substrates. Throughout the respirometry assay, as fuel is actively consumed, we would expect OCR in the presence of etomoxir to be comparable to that in vehicle condition.

To address the reviewer’s comment, we measured maximal respiration stimulated by the substrate palmitoyl-CoA + carnitine **(**please see **Figure R3.1A below)**.

The equation utilized as referenced (Yang *et al*, 2021) is:

Maximal OCR on Palmitoyl-CoA = MAX OCR (FCCP) – Antimycin A OCR.

These values are also provided in Expanded View Table 1 of the revised manuscript and also shown below as **Table R3.1** for ease of reference.

We would like to explain our choice to show state 3 respiration rather than maximal respiration. We rationalize that substrate specific assessment of maximal respiration may not appropriately reflect the physiological effects of mitochondrial architecture on FAO as Ruas *et al*. has shown maximal respiration/uncoupled rates may be underestimated because oligomycin changes spare respiratory capacity (Ruas *et al*). Thus, state 3 respiration (*phosphorylating respiration on Palmitoyl-CoA*) we provide is a more reliable readout than state 3 uncoupled respiration (*Maximal OCR on Palmitoyl-CoA*).

Lastly, we would like to clarify that permeabilization is more of a reductionist experimental system in which physiological regulations are not recapitulated. We use this system to initially assess the association between mitochondrial morphology and FAO, which we then complemented with independent assays in intact cells using fatty acid tracing studies. The following data have now been included in Expanded View Figure 1A -B of the revised manuscript.

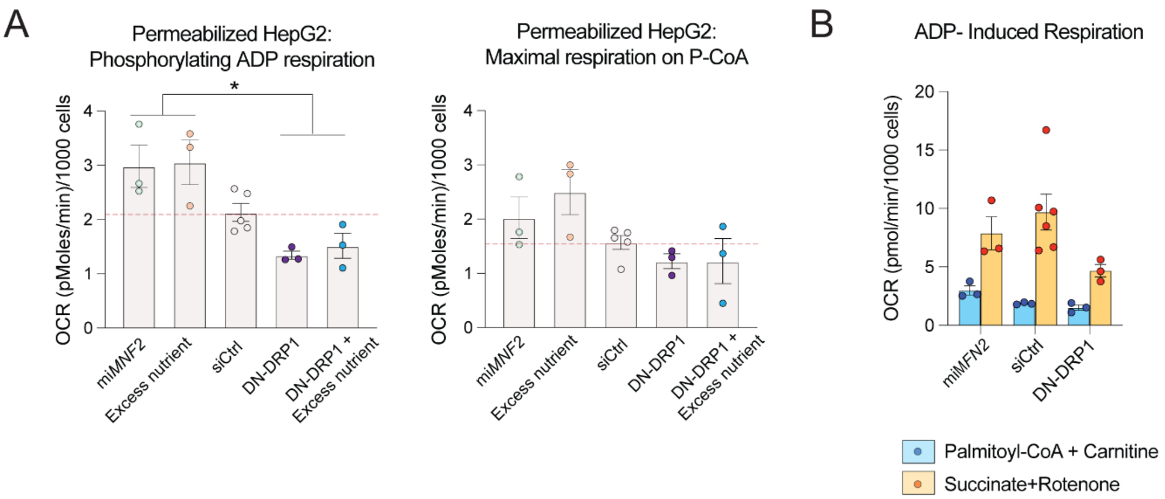

Figure R3.1. *(A) Phosphorylating ADP (state 3) and Maximal OCR values. Conditions that lead to increased mitochondrial fragmentation maintain higher phosphorylating ADP OCR and maximal OCR versus conditions favoring mitochondrial elongation. (B) Phosphorylating ADP respiration in response to palmitoyl-CoA or succinate.*

Treatment Group	Etomoxir Sensitive OCR (Fig1B)	Maximal Etomoxir Sensitive OCR (Fig1B)	State 3 S+R	State3u S+R	Avg Etomoxir Sensitive OCR	Avg Maximal Etomoxir Sensitive OCR	Avg State 3 S+R	Avg State3u S+R
mi*MFN2*	3.757	1.531	10.697	13.918	2.98	2.024	7.860	11.480
	2.666	1.7612	6.572	11.523				
	2.524	2.780	6.337	8.999				
siCtrl	1.786	1.748	6.689	12.123	1.87	1.570	11.040	16.530
	1.978	1.579	16.702	21.149				
	1.849	1.075	9.742	16.327				
		1.654	6.414	9.796				
		1.794	8.485	11.901				
			10.074	12.181				
DN-DRP1	1.109	1.365	5.618	10.339	1.51	1.226	4.670	8.003
	1.909	1.866	4.632	7.703				
	1.528	0.447	3.765	6.052				

Table R3.1. *Mean values of mitochondrial respiration induced by palmitoyl-CoA (column 1 and 2) and succinate (column 3 and 4) from 3 biological replicates. Column 5-8 are mean values of the aggregated 3 biological replicates presented in column 1 -4.*

A control using substrates of the Krebs cycle could have been extremely useful in this respect, and I am surprised that it has not been performed (or provided).

We thank the reviewer for the comment. We have included data on ADP–induced respiration and FCCP-stimulated respiration in response to succinate in Expanded View Figure 1B of the revised manuscript, which are also provided below as **Figure R3.2** for ease of reference. Succinate-driven respiratory rates are all above the values of FA-driven respiration, confirming that the respiratory chain function is not limiting FA-driven respiration at all conditions tested. Indeed, mi*MFN2* increased FAO and thus the decrease in succinate oxidation induced by mi*MFN2* is not reflecting a general constraint on OXPHOS. and Expanded View Figure 6E.

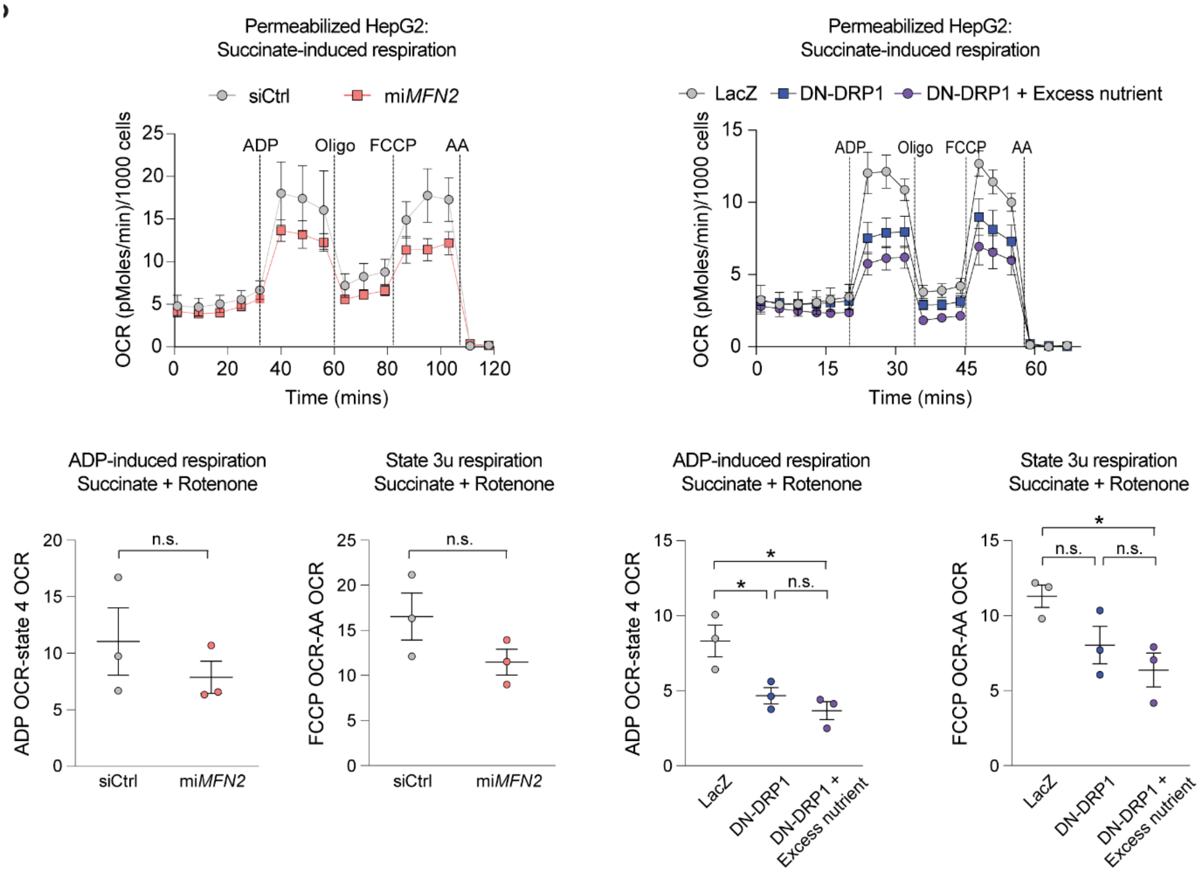

Figure R3.2. *Succinate-induced respiration in permeabilized* siCtrl, mi*MFN2*, and DN-DRP1 *permeabilized HepG2 cells. Traces and data are aggregate of 3 biological replicates with 6 technical repeats.*

The difference in sensitivity to malonyl-CoA is not convincing. I recommend that the authors read the original method, which was developed by Bieber et al in 1972, Anal. Biochem. 50, 509-518 rather than relying on second-hand descriptions and missing the proper references. For example, there is no CPT1 assay in the quoted Shin, SY et al 2006 paper, the correct reference is to another Shin ..... Shin ES et al. (2006). Positive regulation of hepatic carnitine palmitoyl transferase 1A (CPT1A) activities by soy isoflavones and L-carnitine. European Journal of Nutrition 45, 159-164.

The reviewer is raising an important point, which we have addressed with new data in Expanded View Figure 6C of the revised manuscript that are additionally provided below as **Figure R3.3A** for ease of reference. As the reviewer correctly indicated, CoA produced in the presence of D-carnitine alone or in the presence of etomoxir should be subtracted as background CPT1-independent hydrolytic activity.

We have corrected the citation as indicated by the reviewer (Shin *et al*, 2005), and further included the following sentence in the methods section to clarify our approach: "To exclude CoA-SH produced independent of CPT1, CoA-SH was calculated by subtracting CoA produced from D-carnitine in the absence of palmitoyl-CoA."

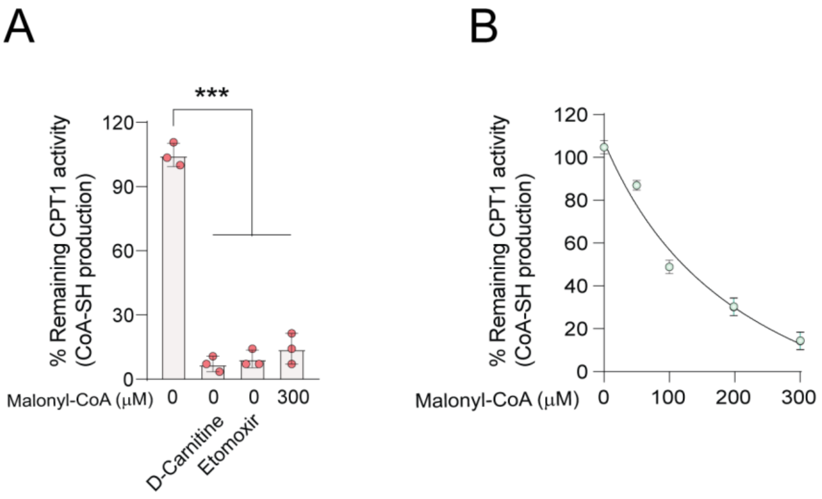

Figure R3.3. *(A) Determining the levels of CPT1-independent CoA release for background calculations. CoA produced in the presence of D-carnitine alone or in the presence of etomoxir is considered background CPT1-independent hydrolytic activity. To calculate the actual CPT1 activity, the value of CPT1-independent CoA release is then subtracted from the total CoA release measurement. (B) Titration curve for CoA-SH produced in CPT1 assays.*

What I would like to see is a full titration with malonyl-CoA (CoASH can also be generated by hydrolases and this is the only way to make sure that activity of CPT1 is being measured) normalized for total protein content.

The requested titrations have now been included in Expanded View Figure 6D in the revised manuscript and additionally provided below as **Figure R3.3B** for ease of reference. We initially focused on 0-40 µM Malonyl-CoA concentrations in CPT1 activity assays as higher concentrations would be supra-physiological (Chien *et al*, 2000; Saha *et al*, 1999), but have now included higher concentrations in the titration experiments. Moreover, comparable levels of CoA-SH present between etomoxir and D-carnitine, ensures that these assays were not confounded by CoA-SH produced via non-CPT1 type of mechanisms **(Figure R3.3A)**. To clarify this, we have now included the following statement on page 25 line 822-824: "To exclude CoA-SH produced independent of CPT1, CoA-SH was calculated by subtracting CoA produced from D-carnitine in the absence of palmitoyl-CoA."

They state that results were normalized to CPT1 expression levels by immunoblots, which are never shown. What is the variability of expression like? How was the quantification carried out?

The requested immunoblots of CPT1 and the corresponding values utilized for normalization have now been included in Expanded View Figure 6A-B and further include below as **Figure R3.4** for ease of reference. No differences were observed in CPT1 protein content between genetic perturbations.

Overall, these data indicate that enhanced FAO in response to genetic or nutrient conditions that increased mitochondrial fragmentation cannot be attributed to changes in CPT1 expression.

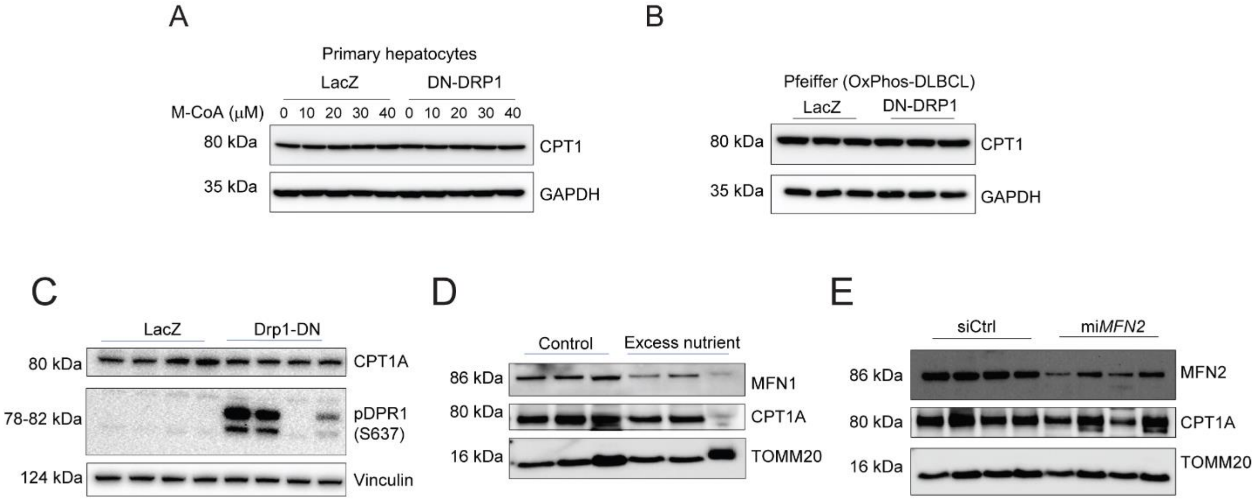

Figure R3.4*. Genetic or nutrient-induced perturbations of mitochondrial morphology do not alter CPT1 protein levels.*

C75 was introduced as a chemically stable inhibitor of mammalian fatty acid synthase in 2000 by Kuhajda et al., Synthesis and antitumor activity of an inhibitor of fatty acid synthase, Proc. Natl. Acad. Sci. USA 97 (2000) 3450-3454 and later shown to activate CPT, Nicot et al (2004) BBRC 325, 660-664. Therefore, there is no mention of it in the indicated reference by Bremer et al 1985.

Thank you for this point, we have corrected this citation error (please see pg12 line 373 of the revised manuscript). We have now also included the Nicot *et al*. and Thupari *et al*. references (Nicot *et al*, 2004; Thupari *et al*, 2002).

About baicailin, in the reference list there is no Dai et al (2019), which is mentioned in the last paragraph or the results. If this was meant to cite Dai et al (2019) Baicalin protects human retinal pigment epithelial cell lines against high glucose-induced cell injury by up-regulation of microRNA-145, Exp Mol Pathol 106, 123-130, this paper has been retracted.

We thank the reviewer for this point, the correct reference (Dai *et al*, 2018) has now been included.

I could not find any evidence in the literature that baicalin acts on CPT1. Rather, it may act by suppressing NFkB and p38 MAPK, Wu et al. (2018) Baicalin alleviates atherosclerosis by relieving oxidative stress and inflammatory responses via inactivating the NF-κB and p38 MAPK signaling pathways, Biomed Pharmacother 97, 1673-1679. Thus, it may not be surprising (or informative) that baicalin had no effects on fatty acid oxidation and this point and all the speculations on mechanism appear untenable.

We thank the reviewer for this valuable comment, which we have addressed both with new data and further clarification of the original data. We have added references showing that baicalin acts on CPT1. Specifically, Figure 3 in Dai *et al* 2018 (also copied here) shows that 100 µM baicalin result in stimulation of CPT1A activity in isolated mitochondria extracts (Dai *et al*, 2018).

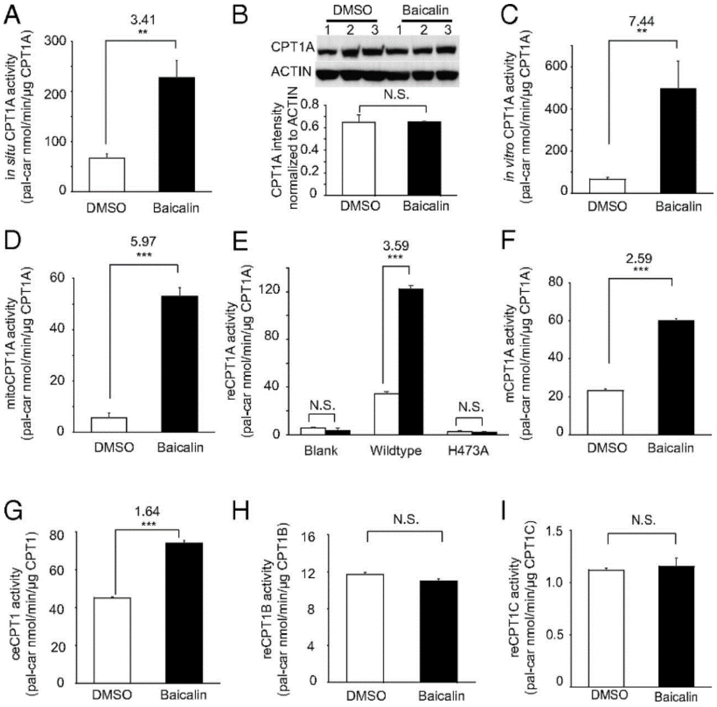

Figure 3 from Dai *et al*., 2018 showing baicalin activates CPT1A. 100 µM baicalin activates CPT1A in HeLa cells, E. coli, M. musculus, and C. elegans.

To further address the comment that baicalin works through NF-κB and p38 MAPK signaling, we note the following:

<u>*First*</u>, if baicalin were to mediate its effect through NF-κB and p38 MAPK signaling, then baicalin treatment would have no effects on palmitoyl-CoA-induced respiration in permeabilized cells because permeabilization removes the contribution of cellular signaling. However, this is not the case as in cells that were treated with baicalin and subsequently permeabilized, increase in respiration induced by palmitoyl-CoA was still observed **(Figure R3.5A)**. This is consistent with baicalin's capacity to activate CPT1.

<u>*Second*</u>, we now include new data in the manuscript showing C75 and baicalin act on CPT1 in Expanded View Figure 6E, which is also provided below in **Figure R3.5B** for ease of reference. Both baicalin and C75 increase ^{13}C incorporation from [U-$^{13}C_{16}$] palmitate into citrate, a readout of FAO employed throughout the paper. Specificity of these activators has also been confirmed using the CPT1 inhibitor etomoxir treatment, which drastically decreased the ^{13}C incorporation into citrate (**Figure R3.5B**).

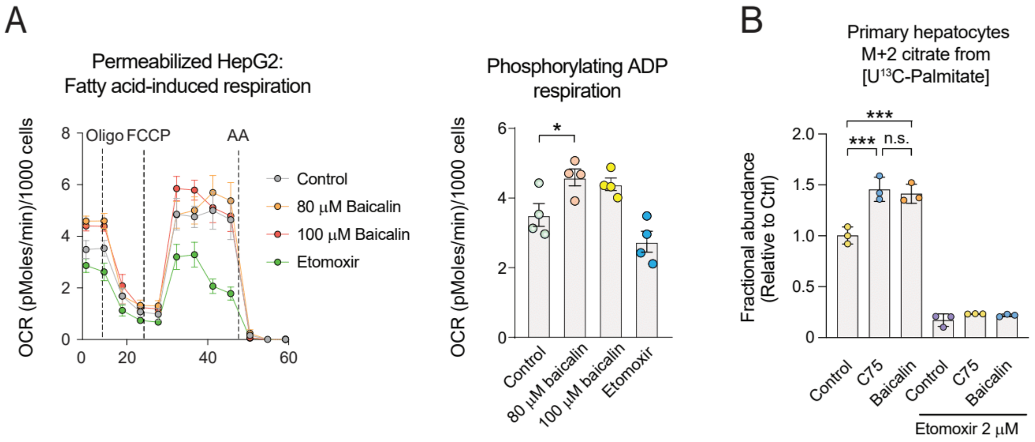

Figure R3.5. *(A) Baicalin increases OCR in response to palmitoyl-CoA in permeabilized HepG2 cells. (B) C75 or baicalin treatment is sufficient to increase palmitate incorporation into citrate in primary hepatocytes.*

References

Chien D, Dean D, Saha AK, Flatt JP & Ruderman NB (2000) Malonyl-CoA content and fatty acid oxidation in rat muscle and liver in vivo. *Am J Physiol Endocrinol Metab* 279

Dai J, Liang K, Zhao S, Jia W, Liu Y, Wu H, Lv J, Cao C, Chen T, Zhuang S, *et al* (2018) Chemoproteomics reveals baicalin activates hepatic CPT1 to ameliorate diet-induced obesity and hepatic steatosis. *Proc Natl Acad Sci U S A* 115: E5896–E5905

Erion KA, Berdan CA, Burritt NE, Corkey BE & Deeney JT (2015) Chronic Exposure to Excess Nutrients Left-shifts the Concentration Dependence of Glucose-stimulated Insulin Secretion in Pancreatic β-Cells. *J Biol Chem* 290: 16191–16201

Merrins MJ, Corkey BE, Kibbey RG & Prentki M (2022) Metabolic cycles and signals for insulin secretion. *Cell Metab* 34: 947–968

Nicot C, Napal L, Relat J, González S, Llebaria A, Woldegiorgis G, Marrero PF & Haro D (2004) C75 activates malonyl-CoA sensitive and insensitive components of the CPT system. *Biochem Biophys Res Commun* 325: 660–664

Reddy JK & Hashimoto T (2001) PEROXISOMAL β-OXIDATION AND PEROXISOME PROLIFERATOR-ACTIVATED RECEPTOR α: An Adaptive Metabolic System.

Ruas JS, Siqueira-Santos ES, Rodrigues-Silva E & Castilho RF High glycolytic activity of tumor cells leads to underestimation of electron transport system capacity when mitochondrial ATP synthase is inhibited OPEN.

Saha AK, Laybutt DR, Dean D, Vavvas D, Sebokova E, Ellis B, Klimes I, Kraegen EW, Shafrir E & Ruderman NB (1999) Cytosolic citrate and malonyl-CoA regulation in rat muscle in vivo. *Am J Physiol* 276

Shin ES, Cho SY, Lee EH, Lee SJ, Chang IS & Lee TR (2005) Positive regulation of hepatic carnitine palmitoyl transferase 1A (CPT1A) activities by soy isoflavones and L–carnitine. *Eur J Nutr 2005 453* 45: 159–164

Thupari JN, Landree LE, Ronnett G V. & Kuhajda FP (2002) C75 increases peripheral energy utilization and fatty acid oxidation in diet-induced obesity. *Proc Natl Acad Sci U S A* 99: 9498–9502

Violante S, Achetib N, van Roermund CWT, Hagen J, Dodatko T, Vaz FM, Waterham HR, Chen H, Baes M, Yu C, *et al* (2019) Peroxisomes can oxidize medium- and long-chain fatty acids through a pathway involving ABCD3 and HSD17B4. *FASEB J* 33: 4355–4364

Yang K, Doan MT, Stiles L & Divakaruni AS (2021) Measuring CPT-1-mediated respiration in permeabilized cells and isolated mitochondria. *STAR Protoc* 2

Dear Orian, dear Nika,

Thank you for submitting your revised manuscript (EMBOJ-2022-111901R) to The EMBO Journal. Your amended study was sent back to the three referees for their re-evaluation, and we have received comments from all of them, which I enclose below. As you will see, the experts stated that the work has been substantially improved by the revisions and they are now broadly in favour of publication.

Thus, we are pleased to inform you that your manuscript has been accepted in principle for publication in The EMBO Journal.

We now need you to take care of a number of minor issues related to formatting and data annotation as detailed below, which should be addressed at re-submission.

Please contact me at any time if you have additional questions related to below points.

As you might remember from previous experience, every paper at the EMBO Journal now includes a 'Synopsis', displayed on the html and freely accessible to all readers. The synopsis includes a 'model' figure as well as 2-5 one-short-sentence bullet points that summarize the article. I would appreciate if you could provide this figure and the bullet points.

Thank you for giving us the chance to consider your manuscript for The EMBO Journal. I look forward to your final revision.

Again, please contact me at any time if you need any help or have further questions.

Best regards,

Daniel

Daniel Klimmeck PhD
Senior Editor
The EMBO Journal

****

Formatting changes required for the revised version of the manuscript:

>> Please provide figures as individual, high-resolution image files. Figure EV6F: ensure C75 label is not cutting off the figure.

>> Adjust the nomenclature of the six EV figures to "Figure EV1, EV2" etc. Correct all callouts in text and legends accordingly.

>> Adjust the title of the 'Conflict of Interests' section to 'Disclosure and Competing Interests Statement'.

>> Author Contributions: Please remove the author contributions information from the manuscript text. Note that CRediT has replaced the traditional author contributions section as of now because it offers a systematic machine-readable author contributions format that allows for more effective research assessment. and use the free text boxes beneath each contributing author's name to add specific details on the author's contribution.
More information is available in our guide to authors.
https://www.embopress.org/page/journal/14602075/authorguide

>> Please a 'Dataset availability section' at the end of the Material & Methods part, stating 'No data amenable to database deposition were generated in this study'.

>> Dataset EV Legends: 2 EV Tables need to be uploaded as separate files and their legends removed from the manuscript and added to the corresponding file.

>> Consider additional changes and comments from our production team as indicated by the .doc file enclosed and leave changes in track mode.

****

Further information is available in our Guide For Authors: https://www.embopress.org/page/journal/14602075/authorguide

We realize that it is difficult to revise to a specific deadline. In the interest of protecting the conceptual advance provided by the work, we recommend a revision within 3 months (20th Mar 2023). Please discuss the revision progress ahead of this time with the editor if you require more time to complete the revisions.

Referee #1:

The Authors have adequately addressed my concerns.

Referee #2:

The authors addressed my concerns, particularly with the inclusion of an additional experiment. The data supports the conclusions of the manuscript, which are novel and important.

Referee #3:

The Authors have resolved all my points of concern and I have no objections to publication of this manuscript

The authors addressed the remaining editorial issues.

Dear Orian, dear Nika,

Thank you for submitting the revised version of your manuscript. I have now evaluated your amended manuscript and concluded that the remaining minor concerns have been sufficiently addressed.

Thus, I am pleased to inform you that your manuscript has been accepted for publication in the EMBO Journal.

Please note that it is EMBO Journal policy for the transcript of the editorial process (containing referee reports and your response letter) to be published as an online supplement to each paper. I would thus like to ask for your consent on keeping the additional referee figures included in this file.

Also, in case you might NOT want the transparent process file published at all, you will also need to inform us via email immediately. More information is available here:
https://www.embopress.org/page/journal/14602075/authorguide#transparentprocess

----

Please note that in order to be able to start the production process, our publisher will need and contact you shortly regarding the page charge authorisation and licence to publish forms.

Authors of accepted peer-reviewed original research articles may choose to pay a fee in order for their published article to be made freely accessible to all online immediately upon publication. The EMBO Open fee is fixed at $6,100 USD / £4,950 GBP / €5,500 EUR (+ VAT where applicable).

We offer two licenses for Open Access papers, CC-BY and CC-BY-NC-ND.
For more information on these licenses, please visit:
http://creativecommons.org/licenses/by-nc-nd/3.0/deed.en_US

Should you be planning a Press Release on your article, please get in contact with embojournal@wiley.com as early as possible, in order to coordinate publication and release dates.

On a different note, I would like to alert you that EMBO Press is currently developing a new format for a video-synopsis of work published with us, which essentially is a short, author-generated film explaining the core findings in hand drawings, and, as we believe, can be very useful to increase visibility of the work. This has proven to offer a nice opportunity for exposure i.p. for the first author(s) of the study. Please see the following link for representative examples and their integration into the article web page:
https://www.embopress.org/video_synopses
https://www.embopress.org/doi/full/10.15252/embj.2019103932

Please let me know, should you be interested to engage in commissioning a similar video synopsis for your work. According operation instructions are available and intuitive.

If you have any questions, please do not hesitate to call or email the Editorial Office.

Thank you again for this contribution to The EMBO Journal and congratulations on a successful publication! Please consider us again in the future for your most exciting work.

Best regards,

Daniel

Daniel Klimmeck, PhD
Senior Editor
The EMBO Journal
EMBO
Postfach 1022-40

Meyerhofstrasse 1
D-69117 Heidelberg
contact@embojournal.org
Submit at: http://emboj.msubmit.net

EMBO Press Author Checklist

Corresponding Author Name: Orian Shirihai	
Journal Submitted to: EMBO Journal	
Manuscript Number: EMBOJ-2022-111901R	

USEFUL LINKS FOR COMPLETING THIS FORM
The EMBO Journal - Author Guidelines
EMBO Reports - Author Guidelines
Molecular Systems Biology - Author Guidelines
EMBO Molecular Medicine - Author Guidelines

Reporting Checklist for Life Science Articles (updated January 2022)
This checklist is adapted from Materials Design Analysis Reporting (MDAR) Checklist for Authors. MDAR establishes a minimum set of requirements in transparent reporting in the life sciences (see Statement of Task: 10.31222/osf.io/9sm4x). Please follow the journal's guidelines in preparing your manuscript.
Please note that a copy of this checklist will be published alongside your article.

Abridged guidelines for figures
1. Data
The data shown in figures should satisfy the following conditions:
- the data were obtained and processed according to the field's best practice and are presented to reflect the results of the experiments in an accurate and unbiased manner.
- ideally, figure panels should include only measurements that are directly comparable to each other and obtained with the same assay.
- plots include clearly labeled error bars for independent experiments and sample sizes. Unless justified, error bars should not be shown for technical replicates.
- if n<5, the individual data points from each experiment should be plotted. Any statistical test employed should be justified.
- Source Data should be included to report the data underlying figures according to the guidelines set out in the authorship guidelines on Data Presentation.

2. Captions
Each figure caption should contain the following information, for each panel where they are relevant:
- a specification of the experimental system investigated (eg cell line, species name).
- the assay(s) and method(s) used to carry out the reported observations and measurements.
- an explicit mention of the biological and chemical entity(ies) that are being measured.
- an explicit mention of the biological and chemical entity(ies) that are altered/varied/perturbed in a controlled manner.
- the exact sample size (n) for each experimental group/condition, given as a number, not a range;
- a description of the sample collection allowing the reader to understand whether the samples represent technical or biological replicates (including how many animals, litters, cultures, etc.).
- a statement of how many times the experiment shown was independently replicated in the laboratory.
- definitions of statistical methods and measures:
  - common tests, such as t-test (please specify whether paired vs. unpaired), simple χ2 tests, Wilcoxon and Mann-Whitney tests, can be unambiguously identified by name only, but more complex techniques should be described in the methods section;
  - are tests one-sided or two-sided?
  - are there adjustments for multiple comparisons?
  - exact statistical test results, e.g., P values = x but not P values < x;
  - definition of 'center values' as median or average;
  - definition of error bars as s.d. or s.e.m.

Please complete ALL of the questions below.
Select "Not Applicable" only when the requested information is not relevant for your study.

Materials

Newly Created Materials	Information included in the manuscript?	In which section is the information available? (Reagents and Tools Table, Materials and Methods, Figures, Data Availability Section)
New materials and reagents need to be available; do any restrictions apply?	Not Applicable	

Antibodies	Information included in the manuscript?	In which section is the information available? (Reagents and Tools Table, Materials and Methods, Figures, Data Availability Section)
For **antibodies** provide the following information:<br>- Commercial antibodies: RRID (if possible) or supplier name, catalogue number and or/clone number<br>- Non-commercial: RRID or citation	Yes	Materials and Methods

DNA and RNA sequences	Information included in the manuscript?	In which section is the information available? (Reagents and Tools Table, Materials and Methods, Figures, Data Availability Section)
**Short novel DNA or RNA including primers, probes**: provide the sequences.	Not Applicable	

Cell materials	Information included in the manuscript?	In which section is the information available? (Reagents and Tools Table, Materials and Methods, Figures, Data Availability Section)
**Cell lines:** Provide species information, strain. Provide accession number in repository **OR** supplier name, catalog number, clone number, and/**OR** RRID.	Yes	Materials and Methods
**Primary cultures:** Provide species, strain, sex of origin, genetic modification status.	Yes	Materials and Methods
Report if the cell lines were recently **authenticated** (e.g., by STR profiling) and tested for mycoplasma contamination.	Yes	Materials and Methods

Experimental animals	Information included in the manuscript?	In which section is the information available? (Reagents and Tools Table, Materials and Methods, Figures, Data Availability Section)
**Laboratory animals or Model organisms:** Provide species, strain, sex, age, genetic modification status. Provide accession number in repository **OR** supplier name, catalog number, clone number, **OR** RRID.	Yes	Materials and Methods
**Animal observed in or captured from the field:** Provide species, sex, and age where possible.	Not Applicable	
Please detail **housing and husbandry conditions**.	Yes	Materials and Methods

Plants and microbes	Information included in the manuscript?	In which section is the information available? (Reagents and Tools Table, Materials and Methods, Figures, Data Availability Section)
**Plants:** provide species and strain, ecotype and cultivar where relevant, unique accession number if available, and source (including location for collected wild specimens).	Not Applicable	
**Microbes:** provide species and strain, unique accession number if available, and source.	Not Applicable	

Human research participants	Information included in the manuscript?	In which section is the information available? (Reagents and Tools Table, Materials and Methods, Figures, Data Availability Section)
If collected and within the bounds of privacy constraints report on age, sex and gender or ethnicity for all study participants.	Not Applicable	

Core facilities	Information included in the manuscript?	In which section is the information available? (Reagents and Tools Table, Materials and Methods, Figures, Data Availability Section)
If your work benefited from core facilities, was their service mentioned in the acknowledgments section?	Yes	Acknowledgements section

Design

Study protocol	Information included in the manuscript?	In which section is the information available? (Reagents and Tools Table, Materials and Methods, Figures, Data Availability Section)
If study protocol has been **pre-registered, provide DOI in the manuscript**. For clinical trials, provide the trial registration number **OR** cite DOI.	Not Applicable	
Report the **clinical trial registration number** (at ClinicalTrials.gov or equivalent), where applicable.	Not Applicable	

Laboratory protocol	Information included in the manuscript?	In which section is the information available? (Reagents and Tools Table, Materials and Methods, Figures, Data Availability Section)
Provide DOI OR other citation details if **external detailed step-by-step protocols** are available.	Not Applicable	

Experimental study design and statistics	Information included in the manuscript?	In which section is the information available? (Reagents and Tools Table, Materials and Methods, Figures, Data Availability Section)
Include a statement about **sample size** estimate even if no statistical methods were used.	Not Applicable	
Were any steps taken to minimize the effects of subjective bias when allocating animals/samples to treatment (e.g. **randomization procedure**)? If yes, have they been described?	Not Applicable	
Include a statement about **blinding** even if no blinding was done.	Not Applicable	
Describe **inclusion/exclusion criteria** if samples or animals were excluded from the analysis. Were the criteria pre-established?<br><br>If sample or data points were omitted from analysis, report if this was due to attrition or intentional exclusion and provide justification.	Not Applicable	
For every figure, are **statistical tests** justified as appropriate? Do the data meet the assumptions of the tests (e.g., normal distribution)? Describe any methods used to assess it. Is there an estimate of variation within each group of data? Is the variance similar between the groups that are being statistically compared?	Yes	Materials and Methods, Figures

Sample definition and in-laboratory replication	Information included in the manuscript?	In which section is the information available? (Reagents and Tools Table, Materials and Methods, Figures, Data Availability Section)
In the figure legends: state number of times the experiment was **replicated** in laboratory.	Yes	Figures
In the figure legends: define whether data describe **technical or biological replicates**.	Yes	Figures

Ethics

Ethics	Information included in the manuscript?	In which section is the information available? (Reagents and Tools Table, Materials and Methods, Figures, Data Availability Section)
Studies involving **human participants**: State details of **authority granting ethics approval** (IRB or equivalent committee(s), provide reference number for approval.	Not Applicable	
Studies involving **human participants**: Include a statement confirming that **informed consent** was obtained from all subjects and that the experiments conformed to the principles set out in the WMA Declaration of Helsinki and the Department of Health and Human Services Belmont Report.	Not Applicable	
Studies involving **human participants:** For publication of **patient photos**, include a statement confirming that consent to publish was obtained.	Not Applicable	
Studies involving experimental **animals**: State details of **authority granting ethics approval** (IRB or equivalent committee(s), provide reference number for approval. Include a statement of compliance with ethical regulations.	Yes	Materials and Methods
Studies involving **specimen and field samples:** State if relevant **permits** obtained, provide details of authority approving study; if none were required, explain why.	Not Applicable	

Dual Use Research of Concern (DURC)	Information included in the manuscript?	In which section is the information available? (Reagents and Tools Table, Materials and Methods, Figures, Data Availability Section)
Could your study fall under dual use research restrictions? Please check biosecurity documents and list of **select agents and toxins** (CDC): https://www.selectagents.gov/sat/list.htm	Not Applicable	
If you used a select agent, is the security level of the lab appropriate and reported in the manuscript?	Not Applicable	
If a study is subject to dual use research of concern regulations, is the name of the **authority granting approval and reference number** for the regulatory approval provided in the manuscript?	Not Applicable	

Reporting

The MDAR framework recommends adoption of discipline-specific guidelines, established and endorsed through community initiatives. Journals have their own policy about requiring specific guidelines and recommendations to complement MDAR.

Adherence to community standards	Information included in the manuscript?	In which section is the information available? (Reagents and Tools Table, Materials and Methods, Figures, Data Availability Section)
State if relevant guidelines or checklists (e.g., **ICMJE, MIBBI, ARRIVE, PRISMA**) have been followed or provided.	Not Applicable	
For **tumor marker prognostic studies,** we recommend that you follow the **REMARK** reporting guidelines (see link list at top right). See author guidelines, under 'Reporting Guidelines'. Please confirm you have followed these guidelines.	Not Applicable	
For **phase II and III randomized controlled trials**, please refer to the **CONSORT** flow diagram (see link list at top right) and submit the CONSORT checklist (see link list at top right) with your submission. See author guidelines, under 'Reporting Guidelines'. Please confirm you have submitted this list.	Not Applicable	

Data Availability

Data availability	Information included in the manuscript?	In which section is the information available? (Reagents and Tools Table, Materials and Methods, Figures, Data Availability Section)
Have **primary datasets** been deposited according to the journal's guidelines (see 'Data Deposition' section) and the respective accession numbers provided in the Data Availability Section?	Not Applicable	
Were **human clinical and genomic datasets** deposited in a public access-controlled repository in accordance to ethical obligations to the patients and to the applicable consent agreement?	Not Applicable	
Are **computational models** that are central and integral to a study available without restrictions in a machine-readable form? Were the relevant accession numbers or links provided?	Not Applicable	
If publicly available data were reused, provide the respective **data citations in the reference list**.	Not Applicable	
